# Alkene insertion reactivity of a *o*-carboranyl-substituted 9-borafluorene†

**DOI:** 10.1039/d2sc02750j

**Published:** 2022-06-02

**Authors:** Tobias Bischof, Xueying Guo, Ivo Krummenacher, Lukas Beßler, Zhenyang Lin, Maik Finze, Holger Braunschweig

**Affiliations:** Institute for Inorganic Chemistry, Julius-Maximilians-Universität Würzburg Am Hubland 97074 Würzburg Germany maik.finze@uni-wuerzburg.de h.braunschweig@uni-wuerzburg.de; Institute for Sustainable Chemistry & Catalysis with Boron, Julius-Maximilians-Universität Würzburg Am Hubland 97074 Würzburg Germany; Department of Chemistry, The Hong Kong University of Science and Technology Clear Water Bay Kowloon Hong Kong P. R. China chzlin@ust.hk

## Abstract

The synthesis of 9-borafluorene with an electron-withdrawing *o*-carboranyl substituent and its reactions with a series of alkenes are described. The *o*-carboranyl substituent is bonded *via* one of the cluster carbon atoms to the boron atom of the 9-borafluorene moiety. In all cases, the reactions afford partly saturated analogs of borepins (*i.e.* 6,7-dihydroborepins) by unprecedented alkene insertion into the endocyclic B–C bond of the borole ring. Comparative studies with 9-bromo-9-borafluorene illustrate the superior insertion reactivity of the carboranyl-substituted derivative. A suite of experimental and computational techniques disclose the unique properties of the 9-borafluorene and provide insight into how the 9-carboranyl substituent affects its chemical reactivity.

## Abstract 2

A 9-carboranyl-substituted 9-borafluorene is reported, which is capable of undergoing efficient ring expansion to 6,7-dihydroborepins by a previously unknown alkene insertion.

## Graphical abstract

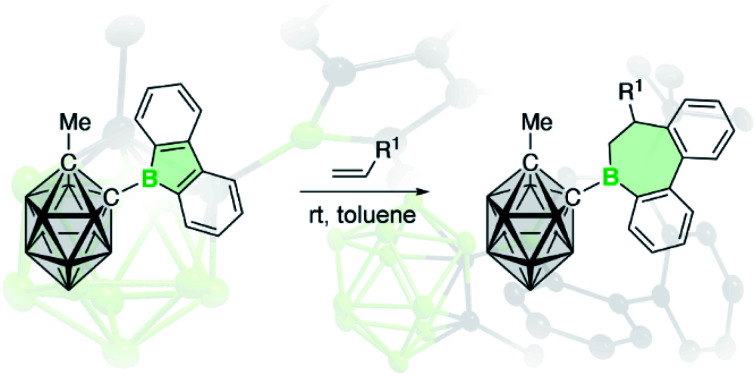

## Introduction

The unique properties of boroles – and particularly their annulated derivatives – are a subject of intense contemporary interest.^1–3^ With the fusion of aromatic ring systems, many of the electronic and thermodynamic properties of boroles are significantly altered from those of the single antiaromatic BC_4_ borole ring.^3^ Annulated boroles such as 9-borafluorenes^3*a*^ offer a potential compromise between stability and desirable properties such as electron-accepting abilities, which could help boroles realize their potential in optical and electronic applications.^3^

Importantly, 9-borafluorenes are still amenable to characteristic modes of borole reactivity, including the propensity to participate in insertion reactions with a wide range of unsaturated molecules.^3*a*^ These reactions can afford a diverse array of conjugated boracycles with properties that are mainly imparted by the three-coordinate boron atom.^4^ Among the more prominent examples are reactions with organic azides to give 9,10-azaboraphenanthrenes, or dibenzofused 1,2-azaborinines,^5,6^ and acetylenes to give seven-membered borepins.^7^ The latter organoborane products can be readily transformed into phenanthrenes, demonstrating their utility in carbon–carbon bond-forming reactions.^7^ The alkyne insertions were shown to be heavily influenced by the boron substituent, with the most efficient insertion reactions being reported for the most Lewis acidic 9-borafluorenes.^7*b*^ Considering that the reaction is initiated by a nucleophilic attack of the substrate on the borole boron atom, this relationship is not surprising and further suggests that the introduction of electron-withdrawing substituents might make 9-borafluorenes more reactive toward the insertion of unsaturated substrates. With this intent, we introduced the strongly electron-accepting *ortho*-dicarba-*closo*-dodecaboranyl substituent bonded *via* a cluster carbon atom on 9-borafluorene. The *ortho*-dicarba-*closo*-dodecaboranyl substituent has been previously shown to increase the Lewis acidity of boranes.^8^ Due to a number of analogies with aromatic hydrocarbons, carboranes have found widespread use in chemistry, pharmaceutical and materials science research.^9^ When attached *via* the carbon atom, both their inductive electron-withdrawing and stabilizing effect on the lowest unoccupied molecular orbital (LUMO) through delocalization should increase the reactivity of the 9-borafluorene.^8,9^

**Scheme 1:**
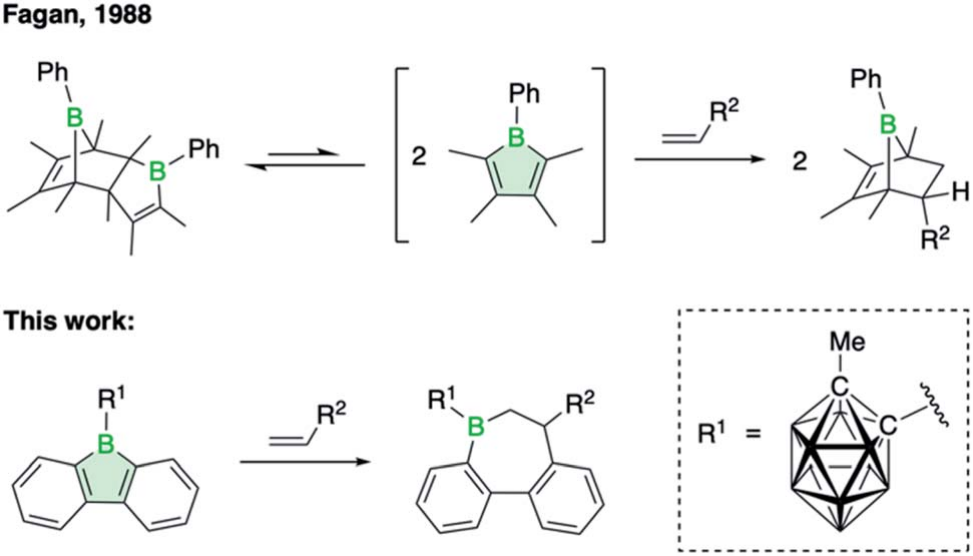
Reactions of boroles with alkenes to give Diels–Alder adducts (top)^12^ and 1,2-insertion products (bottom). All unlabeled vertices of the icosahedral *ortho*-dicarba-*closo*-dodecaborane are BH groups.

Herein, we show that the introduction of a 9-carboranyl group on the 9-borafluorene indeed improves its tendency to undergo insertion reactions, enabling the formation of two-carbon ring expansion products with alkene substrates (see Scheme 1). Unlike alkynes,^1,2*d*,7,10^ alkenes are not known to participate in insertion reactions with boroles. Instead, bicyclic Diels–Alder adducts were observed to form *via* [4 + 2]-cycloaddition with the diene π-system of monocyclic boroles.^11–13^ The first examples of such transformations, dating back to work of Fagan and coworkers, are illustrated in Scheme 1.^12^ Aside from Diels–Alder reactions with a range of terminal and internal alkenes to yield substituted 7-boranorbornenes, the sterically unhindered 1-phenyl-2,3,4,5-tetramethylborole was shown to undergo a spontaneous Diels–Alder dimerization. The unprecedented alkene insertion into endocyclic borole B–C bonds described herein is not unique to the carboranyl-substituted 9-borafluorene but, as we will show, also occurs at elevated temperatures for 9-bromo-9-borafluorene. To examine the reactivity-enhancing effect of the carboranyl substituent, we employed a variety of experimental and computational techniques specifically related to the electronic influence of the boron substituents. Investigations into the mechanism of alkene insertion by density functional theory (DFT) calculations provide further insights into the trends that guide the transformation.

## Results and discussion

### Synthesis and characterization of the borafluorene

**Fig. 1:**
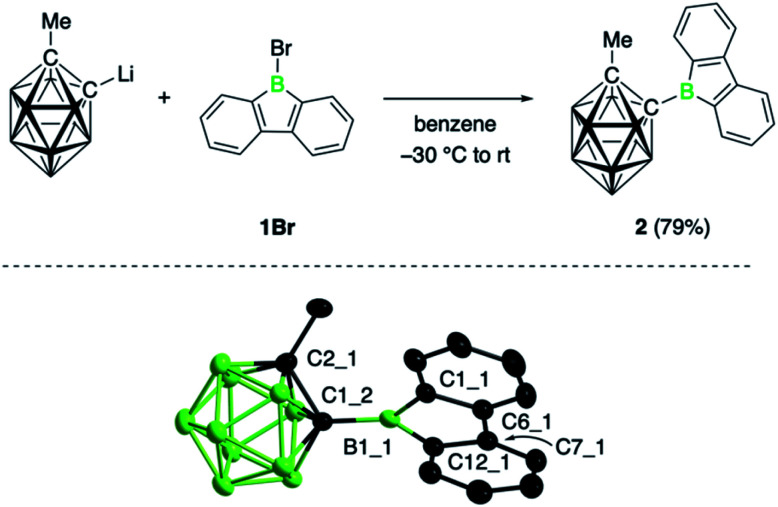
Synthesis and molecular structure of 2. Thermal ellipsoids are drawn at 50% probability. Hydrogen atoms are omitted for clarity. Selected bond distances (Å) and angles (°): B1_1–C1_2 1.595(3), B1_1–C1_1 1.567(3), B1_1–C12_1 1.574(3), C1_2–C2_2 1.666(3); C1_1–B1_1–C12_1 105.2(2).

The target compound was synthesized by salt elimination of the known 9-bromo-9-borafluorene (1Br)^14^ and the 2-lithiated 1-methyl-substituted *ortho*-dicarba-*closo*-dodecaborane, as shown in Fig. 1.^15^ Borafluorene 2 was obtained as an air- and moisture sensitive orange solid in 79% yield and exhibited an ^11^B NMR signal at 65.9 ppm, unchanged from the bromo derivative (1Br, *δ* = 65.9 ppm).^14^ Slow evaporation of a benzene solution of 2 yielded crystalline material suitable for single-crystal X-ray diffraction studies. Its structure along with selected bond distances is shown in Fig. 1; refinement statistics are summarized in the ESI.† The molecular structure of 2 shows the expected planar structure of the borafluorene unit, with B–C and C–C bonds in the borole ring that are comparable to other 9-borafluorene derivatives^16^ and a boron atom that adopts an almost perfectly trigonal planar geometry; the sum of bond angles at B1_1 is 359.9(3)° (Fig. 1). Consequently, the bonding in the borole core is not visibly altered by the cluster substituent. The effect of the borafluorene unit on the bonding situation in the carborane unit is more difficult to assess, as analogous 2-methyl-*ortho*-dicarbaboranes bearing a boryl substituent in the 1-position are rare and only a dimesitylboryl-substituted derivative has been previously crystallographically characterized.^17^ Compared to this species, the C–C (1.666(3) Å) and C–B *exo*-bonds (1.595(3) Å) in 2 are slightly shorter. Moreover, the C–C bond of the carboranyl substituent in 2 is oriented at an angle of 68.6° to the plane of the borafluorene unit such that one hydrogen atom of the methyl group points to the borole boron atom (H⋯B 2.796(3) Å).

To learn more about the effect of the carboranyl substituent on the properties of 2, we determined its reduction potential as well as its Lewis acidity by the Gutmann–Beckett method.^18^ The cyclic voltammogram of 2 in CH_2_Cl_2_ solution shows an irreversible reduction wave at *E*_pc_ = −1.63 V (*vs.* Cp_2_Fe^0/+^), likely corresponding to the formation of the borole-centered radical anion by one-electron reduction.^16*a*^ The first reduction occurs more easily than in 1Br (*E*_pc_ = −2.21 V; onset at −1.84 V) and the 9-phenyl-substituted 9-borafluorene (1Ph, *E*_pc_ = −1.87 V), indicating a more electron-deficient boron center in 2. Gas phase computations at the M062X/6-31G** level of theory^19,20^ are consistent with the electrochemical data in that borafluorene 2 has the lowest lying LUMO in the series (2 (−2.06 eV) > 1Br (−1.57 eV) > 1Ph (−1.39 eV); see Fig. S70†). Quantitative assessment of 2 by the Gutmann–Beckett method in CD_2_Cl_2_ solution indicates that its Lewis acidity, with an acceptor number (AN) of 83.5, is comparable to that of 1Br (AN = 83.8, see Table S1†).^7*b*^ Despite its powerful electrophilicity, the coordination ability of 2 thus seems to be somewhat diminished by the steric bulk of the carboranyl substituent.^9*e*^ This conclusion is reinforced by the relative fluoride ion affinities (FIAs),^21^ as determined computationally at the M062X/6-31G** level of theory.^19,20^ A comparison of a range of borafluorenes with different substituents shows that borafluorene 2 has the highest fluoride ion affinity (−46.0 kcal mol^−1^) and is thus a stronger Lewis acid than 1Br (−31.8 kcal mol^−1^) and 1Ph (−22.2 kcal mol; see ESI†).

As expected from its electronic properties, compound 2 was found to readily form adducts with Lewis bases. Addition of 4-dimethylaminopyridine (DMAP), pyridine, and acetone led to the clean formation of the corresponding Lewis acid–base adducts, as revealed by the characteristic shifts of the ^11^B NMR resonance to lower frequencies (*δ* = −1.6, 0.3 and 8.8 ppm, respectively). Adduct formation of 2 was also observed with the weak donor THF (2-thf, *δ* = 8.7 ppm), in line with other 9-borafluorenes with sterically accessible boron atoms.^3*a*,22^ The complete characterization data of these adducts, including X-ray crystallographic details, are provided in the ESI.†

Through DFT calculations, we sought to identify further effects of the carboranyl substituent on the reactivity of 2. To probe possible changes in the cyclic delocalization of the central BC_4_ ring, we determined its nucleus-independent chemical shift (NICS) value (see Fig. S70†).^23^ A comparison of the bromo-(NICS(1)_zz_ = 6.04 ppm)^24^ and phenyl-substituted derivatives (NICS(1)_zz_ = 6.02 ppm) indicates that the borole ring in 2 (NICS(1)_zz_ = 7.17 ppm) is slightly more antiaromatic. The higher antiaromaticity is again an indication that 2 might display increased reactivity towards insertion, because the antiaromaticity of the BC_4_ ring is lost in the process. A natural population analysis (NPA) of the charge distribution in the three 9-borafluorenes, indicating that the borole boron atom in 2 carries the most positive charge (+0.996 *vs.* +0.890 (1Ph) and +0.651 (1Br)), points to the same conclusion.

To summarize, the 9-carboranyl substituent in borafluorene 2 imparts strong electrophilicity at the boron acceptor site, while its relatively large size does not impede adduct formation or significantly reduce the Lewis acidity. Given the more electrophilic character of 2 compared to the 9-bromo or 9-phenyl derivatives, an even higher reactivity towards unsaturated substrates may be anticipated.

### Reactions of 2 with alkenes

**Scheme 2:**
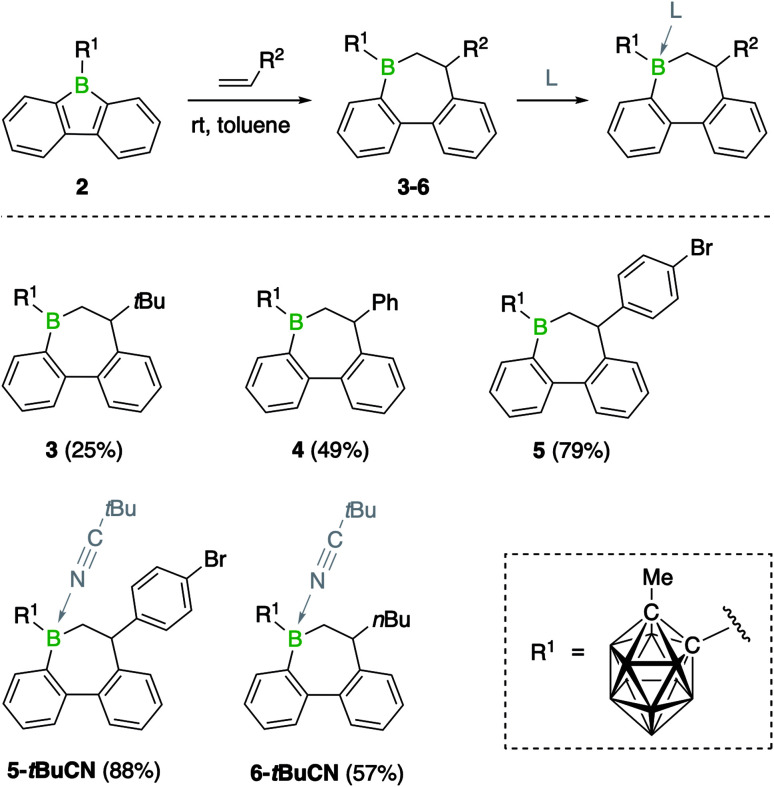
Alkene insertion reactivity of 2.

Previous studies have shown that boroles and their polycyclic derivatives alike are able to undergo insertion reactions with a variety of unsaturated substrates.^2*d*,3*a*^ In view of the fact that the nature of the boron substituent has a major impact on their insertion reactivity,^7*b*^ we were eager to study the reactivity of 9-borafluorene 2. Due to the increased electrophilicity of the borole boron atom brought about by introduction of the carboranyl group, we envisaged that 2 might undergo insertion reactions with less reactive substrates such as alkenes. Indeed, the reaction of borafluorene 2 with an excess of 1-hexene proceeded to completion within 2 h at room temperature, as shown by the shift of the ^11^B NMR resonance to higher frequencies (*δ* = 77.8 ppm). After precipitation from the reaction solution and removal of excess alkene, product 6 was obtained as a colorless oil which was, however, not analytically pure. Isolation in pure form was complicated by the presence of small amounts of impurities from decomposition of the carborane cluster, prompting us to isolate the product as Lewis-acid base adduct. Addition of pivalonitrile (*t*BuCN) to the reaction mixture led to clean formation of 6-*t*BuCN, which was obtained as a colorless solid in 57% yield (Scheme 2). A range of other monosubstituted olefins, including 3,3-dimethylbut-1-ene (neohexene), styrene, and 4-bromostyrene, reacted in a similar manner, albeit with slower reaction rates (Scheme 2). This observation can be attributed to both steric as well as electronic effects of the alkene substituents. While the slower reaction rate observed for the neohexene substrate is likely the result of increased steric bulk, a reduced electron density at the carbon atoms of the double bond due to the electron-withdrawing *para*-bromophenyl group might be the reason in the case of 4-bromostyrene. Despite these effects, complete conversion to the insertion products was achieved in all cases at room temperature. In addition, we noted that using a stoichiometric ratio of reactants, instead of an approximately 10-fold excess of the alkenes, resulted in longer reaction times.§§Products of double insertion were not observed, even at temperatures of 60 °C.

**Fig. 2:**
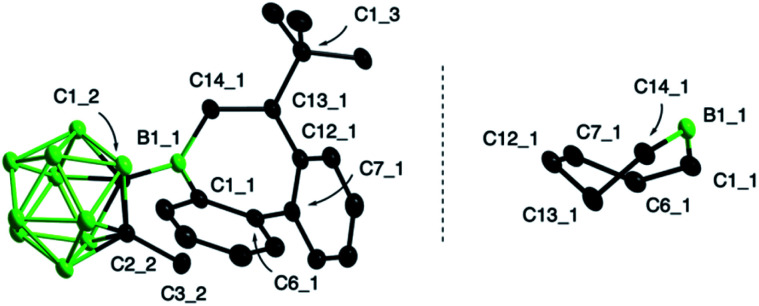
Molecular structure of 3 (left) and view showing the conformation of the borepin ring (right) with displacement parameters at 50% probability level. Hydrogen atoms are omitted for clarity. Selected distances (Å) and angles (°): B1_1–C1_1 1.555(2), B1_1–C14_1 1.567(2), C1_2–C2_2 1.671(2); C1_1–B1_1–C14_1 119.6(1).

As summarized in Scheme 2, the products were either obtained in free form or as adducts with pivalonitrile. After purification by precipitation and several washings with hexane, the insertion products were isolated in yields between 25% and 88%. Full characterization data of the compounds, confirming their identity as 1,2-insertion products, are provided in the ESI.† The molecular structure of 3 will be used as a representative example of the bonding situation in all insertion products. X-ray quality crystals were obtained by vapor diffusion of pentane into a concentrated benzene solution of 3 (Fig. 2). The position of the *tert*-butyl substituent in the seven-membered boracycle indicates that the alkene insertion occurred in a 1,2-fashion. NMR spectroscopic analysis of the crude product 3 did not show the presence of the 2,1-isomer, suggesting that the insertion reaction is highly regioselective and likely governed by steric effects. In addition, due to the use of the unsymmetrical alkene substrate, a quaternary stereocenter is present in the boracycle, making 3 chiral. Although only the *S* isomer is depicted in Fig. 2, compound 3 crystallizes as a racemate. With a total of 4π electrons from the two carbon–carbon double bonds (1.420(2) and 1.412(2) Å) that are part of the aromatic system of the annulated benzene rings, the boracycle represents a 6,7-dihydroborepin and thus a partly saturated analog of a borepin. The BC_6_ ring adopts a twisted boat-like conformation in which the boron atom maintains its trigonal planar coordination environment (sum of bond angles at B1_1 is 360.0(2)°). Consistent with ring expansion from five to seven atoms, the endocyclic C–B–C angle is widened from 105.2(2)° in 2 to 119.6(1)° in 3. In a manner similar to 2, the methyl group of the carboranyl substituent is oriented toward the vacant acceptor orbital on the borole boron atom. The C–C distance (1.671(2) Å) in the carborane remains unchanged from that of 2 (1.666(3) Å) within three standard deviations.

**Scheme 3:**
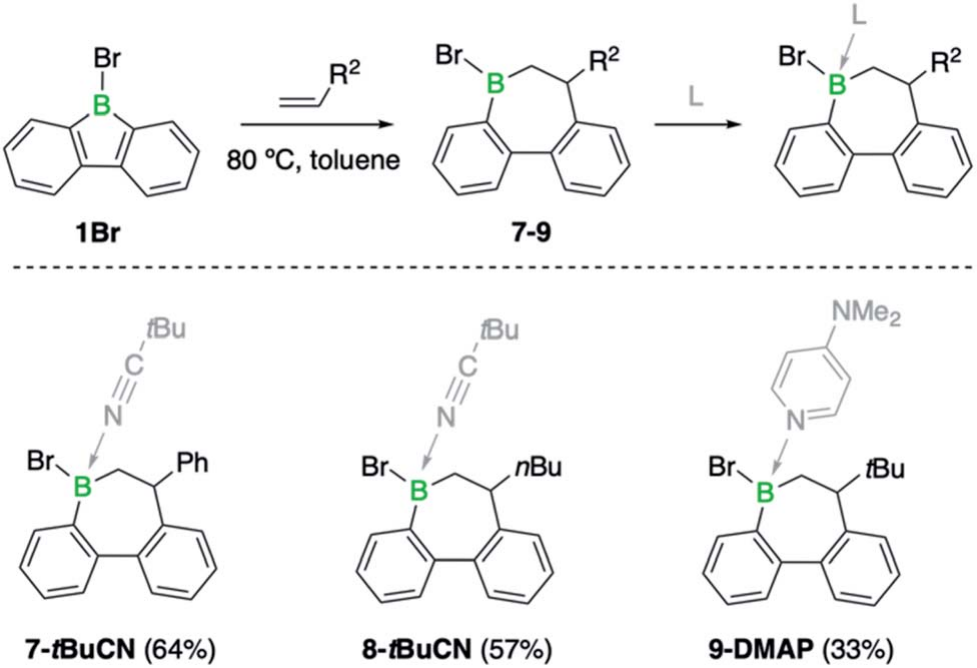
Alkene insertion reactivity of 1Br (DMAP = 4-dimethylaminopyridine).

To contextualize the increased insertion reactivity of 2, we compared its reactivity with 9-bromo-9-borafluorene 1, which was previously shown to have a high rate of alkyne insertion.^7*b*^ Treatment of 1 with 1-hexene, neohexene or styrene led to no reaction at room temperature, even by using the alkene substrates in large excess. Nonetheless, heating the reaction mixtures to 80 °C induced a slow conversion to the respective borepin insertion products. As shown by ^11^B NMR spectroscopy, complete formation of products 7, 8, and 9 was only observed after several days or even weeks (Scheme 3 and see ESI†). As for borafluorene 2, the sterically least encumbered 1-hexene was found to be the most efficient substrate and the use of Lewis bases such as pivalonitrile and 4-dimethylaminopyridine (DMAP) proved effective for the isolation of the ring expansion products. The adducts were fully characterized by NMR spectroscopy, high-resolution mass spectrometry, elemental analysis and single-crystal X-ray diffraction (see ESI† for details). It is worth mentioning that the presence of an additional chiral center at boron gives rise to the possibility of geometric isomers; we obtained 9-DMAP as a mixture of diastereomers in a ratio of *ca.* 4 : 1, but only single diastereomers were observed for the other adducts.

### DFT analysis of the mechanism

The comparison between the two borafluorenes illustrates the striking capacity of 2 for olefin insertion reactions and to furnish 6,7-dihydroborepins. To explore the factors contributing to the large difference in reactivity between borafluorenes 1 and 2, we turned to DFT methods and first developed an understanding of the mechanism and energetics of the insertion reaction. All computed compounds, the structures of which are identical to the experimental ones, are denoted with a prime symbol (′).

**Fig. 3:**
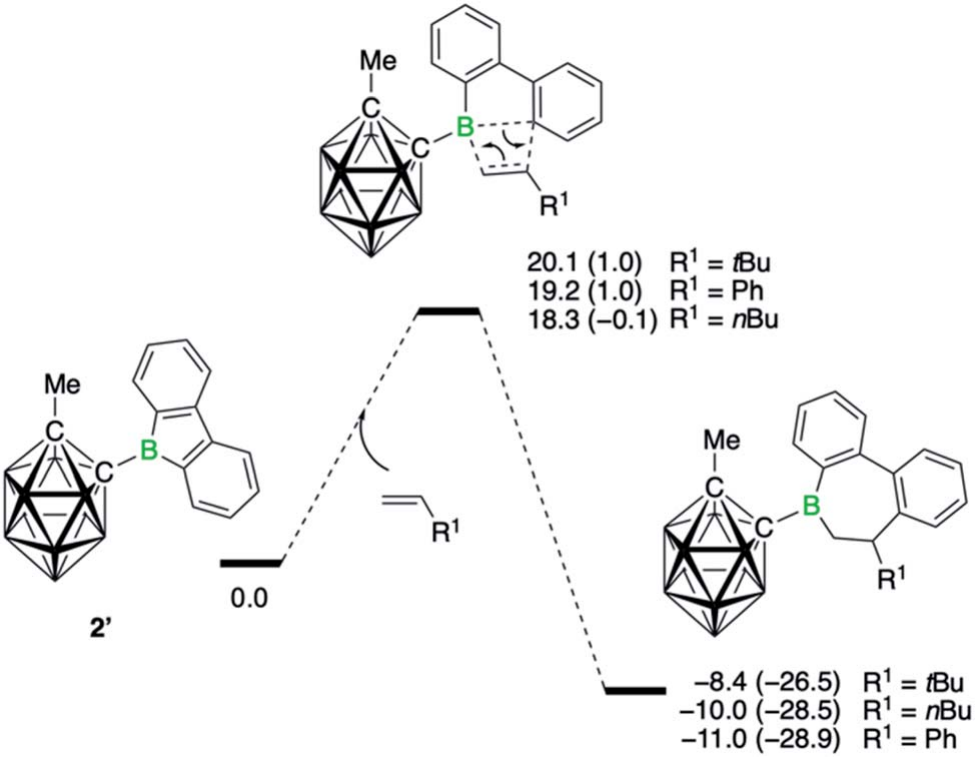
Energy profiles calculated for alkene 1,2-insertion into a B–C bond of the carboranyl-substituted borafluorene 2′. Relative free energies and electronic energies (in parenthesis) are given in kcal mol^−1^.

The energy profile for the insertion reaction of three monosubstituted olefins with 9-carboranyl-substituted borafluorene 2′ is displayed in Fig. 3. It shows a concerted process for the 1,2-insertion with one transition state. The lowest energy barrier (18.3 kcal mol^−1^) was found for the sterically least hindered alkene derivative 1-hexene, whereas the barrier is highest (20.1 kcal mol^−1^) for neohexene, where steric repulsions between the *tert*-butyl substituent and the borafluorene ring are expected (Fig. S72†). The calculated barriers for all three insertions are consistent with the experimental observation that the reactions proceed at room temperature. Corresponding transition states for a 2,1-insertion have significantly higher energy barriers, mainly because of higher steric hindrance in the four-membered transition states as indicated by the longer separations of the newly forming B–C bonds (Fig. S74†). The polarization of the alkene π bond by the alkyl group, and more strongly by the phenyl group, toward the terminal olefinic carbon atom means that the 2,1-insertion is also electronically disfavored. In short, both steric and electronic factors explain the experimentally observed regiochemistry.

Compared to the carboranyl derivative 2′, the reaction profiles for the 1,2-insertion of the three monosubstituted olefins with both the 9-bromo- (1Br′) and 9-phenyl-9-borafluorene (1Ph′) are associated with higher energy barriers (see ESI for details, Fig. S75 and S77†). The calculated barriers for 1-hexene insertion are 24.5 (1Br′) and 22.3 kcal mol^−1^ (1Ph′), respectively. That 1Br′ shows a higher barrier for insertion relative to 2′ corroborates the experimental observation that 1Br requires higher temperatures to form the insertion products. Interestingly, the barrier for borafluorene 1Br′ is the highest, despite being the most Lewis acidic of the series.¶¶Presumably due to the π-donor ability of the bromine atom, the electrophilic character of the boron atom in 1Br is lower than that of 1Ph, which might explain the slightly higher barrier for insertion. The steric disadvantage of the carboranyl substituent is apparently more than offset by its electronic effects.^9*e*^ Due to its strong electron-withdrawing effect, which is comparable to that of a 2,3,5,6-tetrafluorophenyl group,^25^ electron density is removed from the boron atom, making it more electron poor and thus more receptive for nucleophilic attack by the alkene. From both the experimental and theoretical data, it becomes clear that the alkene insertion reaction is dominated by the electronic properties of the boron substituents. That is, substituents that increase the electrophilicity at the boron atom favor the insertion of the alkenes. On the other hand, the Lewis acidity, which also factors in steric effects, does not play an important role. Hence the trend observed for alkyne insertions, where the reaction rates were found to correlate with the Lewis acidity (as determined by the Gutmann–Beckett method) of the 9-borafluorenes,^7*b*^ cannot be extended to these alkene insertions.

## Conclusions

A 9-carboranyl-substituted 9-borafluorene has been successfully synthesized and comprehensively characterized with the goal of elucidating the effect of the boron substituent on the electronic properties and reactivity of the 9-borafluorene. Results from both experimental and theoretical studies indicate a strongly electrophilic and Lewis acidic character, making the 9-borafluorene prone to nucleophilic addition reactions. Comparative reactivity studies with 9-bromo-9-borafluorene revealed its pronounced tendency to undergo unprecedented insertion reactions with alkenes to form 6,7-dihydroborepins: the carboranyl-substituted 9-borafluorene inserts alkenes into the B–C bond of the borole ring much more readily than the bromo derivative. The Lewis acidity, which was shown to be a good measure for their propensity to undergo alkyne insertions, fails to explain the dramatic difference in their reactivity as both 9-borafluorenes display similar Lewis acidities based on the Gutmann acceptor number (AN). Instead, the increased insertion reactivity towards monosubstituted alkenes seems to be favored by the more electrophilic character of the borafluorene imparted by the electron-withdrawing carboranyl substituent. Its greater steric bulk compared to the bromo or phenyl group suggests that steric effects of the boron substituents play only a secondary role in these reactions. Further studies directed towards the extension of their insertion reactivity with a range of other substrates are currently underway.

## Data availability

All experimental procedures, spectral data, and computational data are included in the ESI.†

## Author contributions

T. B. carried out the synthetic work and analytical characterization, including the crystallographic studies. X. G. carried out the computational studies. L. B. helped with the synthesis of some of the precursor molecules. Z. L., M. F., and H. B. assisted with data analysis and directed the research. I. K., T. B., and X. G. wrote the manuscript, with input from all authors.

## Conflicts of interest

There are no conflicts to declare.

## Supplementary Material

SC-013-D2SC02750J-s001

SC-013-D2SC02750J-s002
