# Supplementary material for: Alkene insertion reactivity of a o-carboranyl-substituted 9-borafluorene
Source: Chem Sci. 2022 Jun 2;13(25):7492–7. doi: 10.1039/d2sc02750j (PMC9244080; doi:10.1039/d2sc02750j)
Supplement: SC-013-D2SC02750J-s001 [file SC-013-D2SC02750J-s001.pdf]

Electronic Supplementary Material (ESI) for Chemical Science.
This journal is © The Royal Society of Chemistry 2022

Supporting Information for

# Alkene insertion reactivity of a *o*-carboranyl-substituted 9-borafluorene

Tobias Bischof,[†,ab] Xueying Guo,[†,c] Ivo Krummenacher,[ab] Lukas Beßler,[ab] Zhenyang Lin,[*c] Maik Finze*[ab] and Holger Braunschweig*[ab]

[a]*Institute for Inorganic Chemistry, Julius-Maximilians-Universität Würzburg, Am Hubland, 97074 Würzburg, German.*

[b]*Institute for Sustainable Chemistry & Catalysis with Boron, Julius-Maximilians-Universität Würzburg, Am Hubland, 97074 Würzburg, Germany.*

[c]*Department of Chemistry, The Hong Kong University of Science and Technology, Clear Water Bay, Kowloon, Hong Kong (P.R. China).*

*† T.B. and X.G. contributed equally to this work.*

TABLE OF CONTENTS

1. Methods and materials ..... 3
2. Experimental section ..... 4
2.1 NMR numbering scheme ..... 4
2.2 Synthesis of **1** ..... 4
2.3 Synthesis of **2** ..... 5
2.4 Synthesis of **2-thf** ..... 6
2.5 Synthesis of **2-acetone** ..... 7
2.6 Synthesis of **2-pyridine** ..... 8
2.7 Synthesis of **2-DMAP** ..... 9
2.8 Synthesis of **3** ..... 10
2.9 Synthesis of **4** ..... 11
2.10 Synthesis of **5** ..... 12
2.11 Synthesis of **5-*t*BuCN** ..... 13
2.12 Synthesis of **6-*t*BuCN** ..... 14
2.13 Synthesis of **7-*t*BuCN** ..... 15
2.14 Synthesis of **8-*t*BuCN** ..... 16
2.15 Synthesis of **9-DMAP** ..... 17
3. NMR spectra ..... 20
4. IR spectra ..... 41
5. Cyclic voltammetry studies ..... 48
6. X-ray crystallographic data ..... 50
7. Computational details ..... 62
7.1 Electronic properties ..... 62
7.2 Mechanistic studies ..... 63
7.3 Cartesian coordinates ..... 67
8. References ..... 113

# 1. Methods and materials

All manipulations were performed either under an atmosphere of dry argon or *in vacuo* using standard Schlenk line or glovebox techniques. Deuterated solvents were dried over molecular sieves and degassed by three freeze-pump-thaw cycles prior to use. All other solvents were degassed and distilled from appropriate drying agents. Both deuterated and non-deuterated solvents were stored under argon over activated 4 Å molecular sieves. NMR spectra were acquired on a Bruker Avance 500 or a Bruker Avance 400 NMR spectrometer. Chemical shifts (δ) are reported in ppm and internally referenced to the carbon nuclei ($^{13}C\{^{1}H\}$) or residual protons ($^{1}H$) of the solvent. Heteronuclei NMR spectra are referenced to external standards ($^{11}B$: $BF_3{\cdot}OEt_2$). Solid-state IR spectra were recorded on a Bruker FT-IR spectrometer ALPHA II inside a glovebox. Microanalyses (C, H, N, S) were performed on an Elementar vario MICRO cube elemental analyzer. High-resolution mass spectrometry (HRMS) data were obtained from a Thermo Scientific Exactive Plus spectrometer. Unless otherwise stated, solvents and reagents were purchased from Sigma-Aldrich, Alfa Aesar, VWR or TCI. 2,2'-Dilithium-1,1'-biphenyl[1,2] and 1-methyl-2-lithium-*ortho*-dicarba-*closo*-dodecaborane[3,4] were synthesized by following literature procedures.

# 2. Experimental section

## 2.1 NMR numbering scheme

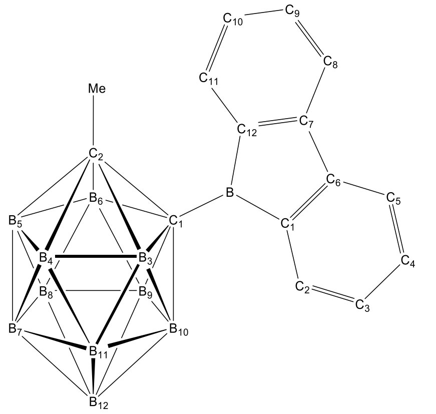


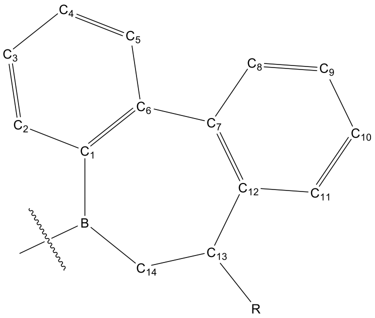


## 2.2 Synthesis of **1**

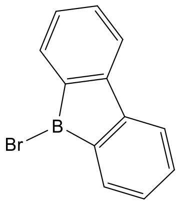


The synthesis of 9-bromo-9-borafluorene (**1**) was adopted from a literature report by Rivard.[2] 2,2'-Dilithium-1,1'-biphenyl (3.24 g, 19.51 mmol) was dissolved in 120 mL toluene and cooled to −78 °C. A solution of $BBr_3$ (1.8 mL, 18.97 mmol) in toluene (10 mL) was added dropwise with stirring. The resulting yellow suspension was allowed to warm to room temperature overnight. The mixture was filtered and all volatiles of the yellow solution were removed under vacuum. The crude product was recrystallized from hexane at −30 °C. Spectroscopic data are consistent with literature values.

Yield: 3.31 g (13.66 mmol, 72%) of a yellow, needle-shaped crystalline solid.

**$^1$H NMR** (500 MHz, $C_6D_6$): $\delta$ (ppm) = 7.43 (d, 2H, $^3J_{HH}$ = 7.1 Hz, Ar*H*), 6.95 (dd, 2H, $^3J_{HH}$ = 7.4 Hz, $^4J_{HH}$ = 1.3 Hz, Ar*H*), 6.91 (dd, 2H, $^3J_{HH}$ = 7.3 Hz, $^4J_{HH}$ = 1.1 Hz, Ar*H*), 6.76 (dd, 2H, $^3J_{HH}$ = 7.3 Hz, $^4J_{HH}$ = 1.1 Hz Ar*H*).

**$^{11}$B NMR** (160 MHz, $C_6D_6$): $\delta$ (ppm) = 65.9 (s, 1B, $B_{ring}$).

## 2.3 Synthesis of **2**

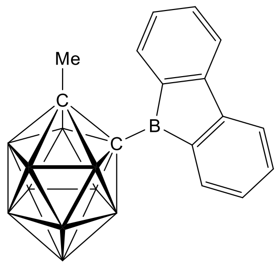


9-Bromo-9-borafluorene (**1**, 2.50 g, 10.29 mmol) was dissolved in 10 mL toluene and the mixture was cooled to −30 °C. 1-Methyl-2-lithium-*ortho*-dicarba-*closo*-dodecaborane (1.69 g,10.29 mmol) was suspended in 5 mL toluene and added dropwise over 2 h. The resulting suspension was filtered and the orange solution concentrated under vacuum. The addition of hexane (10 mL) resulted in precipitation of an orange solid. The solid was washed with hexane (5 x 5 mL) and dried *in vacuo*. Suitable crystals for X-ray diffraction analysis were obtained by slow evaporation of a saturated solution of **2** in benzene.

Yield: 2.59 g (8.09 mmol, 79%) of an orange solid.

**$^{1}$H{$^{11}$B} NMR** (500 MHz, $C_6D_6$): $\delta$ = 7.65 (d, 2H, $^{3}J_{HH}$ = 7.3 Hz, C2+11$_{aryl}$−*H*), 6.86 (dd, 2H, $^{3}J_{HH}$ = 7.4 Hz, $^{4}J_{HH}$ = 1.2 Hz, C4+9$_{aryl}$−*H*), 6.78 (d, 2H, $^{3}J_{HH}$ = 7.3 Hz, C5+8$_{aryl}$−*H*), 6.69 (dd, 2H, $^{3}J_{HH}$ = 7.4 Hz, $^{4}J_{HH}$ = 1.1 Hz, C3+10$_{aryl}$−*H*), 3.25 (s, 1H, B12−*H*), 3.06 (s, 2H, B8+11−*H*), 2.92 (s, 3H, B4+5+7−*H*), 2.68 (s, 2H, B3+6−*H*), 2.53 (s, 2H, B9+10−*H*), 1.26 (s, 3H, C*H*$_3$) ppm.

**$^{11}$B NMR** (160 MHz, $C_6D_6$): $\delta$ = 65.9 (s, 1B, B$_{ring}$), 2.0 (d, 1B, *B*12), −5.3 (d, 1B, *B*7), −6.7 (d, 2B, *B*8+11), −8.3 (d, 2B, *B*9+10), −9.0 (d, 2B, *B*4+5), −9.9 (d, 2B, *B*3+6) ppm.

**$^{13}$C{$^{1}$H} NMR** (126 MHz, $C_6D_6$): $\delta$ = 154.93 (s, 2C, *C*1+12$_{aryl}$−B), 136.81 (s, 2C, *C*2+11$_{aryl}$), 135.96 (s, 2C, *C*6+7$_{aryl}$), 128.81 (s, 2C, *C*3+10$_{aryl}$), 128.59 (s, 2C, *C*5+8$_{aryl}$), 120.12 (s, 2C, *C*4+9$_{aryl}$), 76.41 (s, 1C, *C*$_{cage}$−CH$_3$), 71.48 (s, 1C, *C*$_{cage}$−B), 25.06 (s,1C, C$_{cage}$−*C*H$_3$) ppm.

**HRMS** (LIFDI): calculated for **2**: 320.2739; found: 320.2728.

**Elemental analysis** (%) calcd. for $C_{15}H_{21}B_{11}$: C 56.26, H 6.61; found: C 57.24, H 7.11.

**IR spectroscopy** [$\tilde{\nu}$]: 3046 cm$^{-1}$ (*C$_{aryl}$−H*, w), 2561 cm$^{-1}$ (*B−H*, s), 1600 cm$^{-1}$ (*C=C*, s), 1429 cm$^{-1}$ (*C=C*, s).

*General note: Due to the overlap of the broad $^{11}$B NMR resonances, the coupling constants for the boron atoms of the carboranyl substituent could not be unambiguously determined.*

## 2.4 Synthesis of **2-thf**

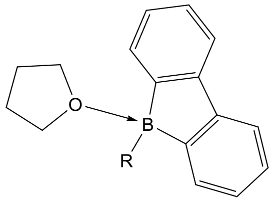


9-Carboranyl-9-borafluorene (**2**, 150 mg, 0.47 mmol) was dissolved in 3 mL toluene, and 0.1 mL of tetrahydrofuran in 1 mL of toluene was added. The resulting reaction mixture was stirred for 1 h at room temperature. Hexane (5 mL) was added to the reaction mixture, resulting in a white precipitate. The solid was washed with hexane (5 x 1 mL) and dried *in vacuo*. Crystals suitable for X-ray diffraction analysis were obtained by diffusing pentane into a toluene solution of **2-thf**.

Yield: 161 mg (0.41 mmol, 87%) of a white solid.

**$^{1}$H{$^{11}$B} NMR** (500 MHz, $C_6D_6$): $\delta$ = 7.62 (dm, 2H, $^{3}J_{HH}$ = 7.4 Hz, C2+11$_{aryl}$−*H*), 7.50 (dm, 2H, $^{3}J_{HH}$ = 7.5 Hz, C5+7$_{aryl}$−*H*), 7.21 (td, 2H, $^{3}J_{HH}$ = 7.4 Hz, $^{4}J_{HH}$ = 1.2 Hz, C4+9$_{aryl}$−*H*), 7.12 (td, 2H, $^{3}J_{HH}$ = 7.5 Hz, $^{4}J_{HH}$ = 1.3 Hz, C3+10$_{aryl}$−*H*), 3.47 (m, 4H. O(C$H_2$)$_2$ ), 3.18 (s, 1H, B−*H*), 3.04 (s, 1H, B−*H*), 2.98 (s, 2H, B−*H*), 2.83 (s, 2H, B−*H*), 2.57 (s, 2H, B−*H*), 2.50 (s, 2H, B−*H*), 1.02 (s, 3H, C$H_3$), 0.92 (s, 4H, (C$H_2$)$_2$) ppm.

**$^{11}$B NMR** (160 MHz, $C_6D_6$): $\delta$ = 8.7 (s, 1B, B$_{ring}$), −0.6 (d, 1B, *B*12), −4.9 (d, 1B, *B*7), −8.1 (d, 2B), −8.6 (d, 4B), −10.0 (d, 2B) ppm.

**$^{13}$C{$^{1}$H} NMR** (126 MHz, $C_6D_6$): $\delta$ = 149.89 (s, 2C, *C*6+7$_{aryl}$), 147.07 (s, 2C, *C*1+12$_{aryl}$−B), 134.09 (s, 2C, *C*2+11$_{aryl}$), 129.17 (s, 2C, *C*4+9$_{aryl}$), 126.76 (s, 2C, *C*3+10$_{aryl}$), 119.90 (s, 2C, *C*5+8$_{aryl}$), 78.75 (s, 1C, *C*$_{cage}$−B), 76.60 (s, 1C, *C*$_{cage}$−$CH_3$), 78.75 (s, 2C, O-(*C*$H_2$)$_2$), 24.64 (s, 1C, C$_{cage}$−*C*$H_3$), 24.49 (s, 2C, (*C*$H_2$)$_2$) ppm.

**Elemental analysis** (%) calcd. for $C_{19}H_{29}B_{11}O$: C 58.16, H 7.45; found: C 62.61, H 7.70.

**IR spectroscopy** [$\tilde{\nu}$]: 3048 cm$^{-1}$ (*C$_{aryl}$−H*, w), 2557 cm$^{-1}$ (*B−H*, s), 1593 cm$^{-1}$ (*C=C*, m), 1430 cm$^{-1}$ (*C=C*, s).

## 2.5 Synthesis of **2-acetone**

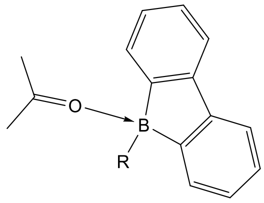


9-Carboranyl-9-borafluorene (**2**) (150 mg, 0.47 mmol) was dissolved in 3 mL toluene, and 0.1 mL of acetone in 1 mL of toluene was added. The resulting reaction mixture was stirred for 1 h at room temperature. Hexane (5 mL) was added to the reaction mixture, resulting in a white precipitate. The solid was washed with hexane (5 x 1 mL) and dried *in vacuo*. Crystals suitable for X-ray diffraction analysis were obtained by diffusing pentane into a benzene solution of **2-acetone**.

Yield: 117 mg (0.31 mmol, 66%) of a white solid.

**$^{1}H\{^{11}B\}$ NMR** (500 MHz, $C_6D_6$): $\delta$ = 7.56 (dm, 2H, $^{3}J_{HH}$ = 7.1 Hz, C2+11$_{aryl}$–*H*), 7.44 (dm, 2H, $^{3}J_{HH}$ = 7.5 Hz, C5+8$_{aryl}$–*H*), 7.19 (td, 2H, $^{3}J_{HH}$ = 7.4 Hz, $^{4}J_{HH}$ = 1.3 Hz, C4+9$_{aryl}$–*H*), 7.10 (td, 2H, $^{3}J_{HH}$ = 7.3 Hz, $^{4}J_{HH}$ = 1.1 Hz, C3+10$_{aryl}$–*H*), 3.18 (s, 2H, B–*H*), 2.98 (s, 4H, B–*H*), 2.93 (s, 2H, B–*H*), 2.57 (s, 2H, B–*H*), 1.12(s, 3H, C*H*$_3$), 0.95(s, 3H, OC(C*H*$_3$)$_2$) ppm.

**$^{11}B$ NMR** (160 MHz, $C_6D_6$): $\delta$ = 8.8 (s, 1B, B$_{ring}$), −1.1 (d, 1B, *B*12), −4.3 (d, 1B, *B*7), −8.0 (d, 4B), −9.4 (d, 4B) ppm.

**$^{13}C\{^{1}H\}$ NMR** (126 MHz, $C_6D_6$): $\delta$ = 227.14 (s, 1C, O–*C*), 149.9 (s, 2C, *C*6+7$_{aryl}$), 148.35 (s, 2C, *C*1+12$_{aryl}$–B), 132.87 (s, 2C, *C*2+11$_{aryl}$), 129.35 (s, 2C, *C*4+9$_{aryl}$), 127.35 (s, 2C, *C*3+10$_{aryl}$), 120.03 (s, 2C, *C*5+8$_{aryl}$), 78.56 (s, 1C, *C*$_{cage}$–B), 74.66 (s, 1C, *C*$_{cage}$–CH$_3$), 29.52 (s, 2C, OC(*C*H$_3$)$_2$), 23.25 (s, 1C, C$_{cage}$–*C*H$_3$) ppm.

**Elemental analysis** (%) calcd. for $C_{18}H_{27}B_{11}O$: C 57.15, H 7.19; found: C 57.01, H 7.22.

**IR spectroscopy** [$\tilde{v}$]: 3042 cm$^{-1}$ (*C$_{aryl}$–H*, w), 2565 cm$^{-1}$ (*B–H*, s), 1623 cm$^{-1}$ (*C=O*, m), 1432 cm$^{-1}$ (*C=C*, s).

## 2.6 Synthesis of **2-pyridine**

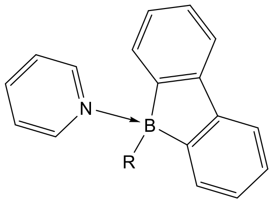


9-Carboranyl-9-borafluorene (**2**, 150 mg, 0.47 mmol) was dissolved in 3 mL toluene, and 0.1 mL of pyridine in 1 mL toluene was added. The reaction mixture was stirred for 1 h at room temperature before hexane (5 mL) was added to precipitate the product. The solid was washed with hexane (5 x 1 mL) and dried *in vacuo*. Crystals suitable for X-ray diffraction analysis were obtained by diffusing pentane into a benzene solution of **2-pyridine.**

Yield: 152 mg (0.38 mmol, 81%) of a white solid.

**$^{1}H\{^{11}B\}$ NMR** (500 MHz, $C_6D_6$): $\delta$ = 9.35 (dm, 2H, C2+6$_{pyridine}$−*H*), 8.10 (m, 1H, C2+11$_{aryl}$−*H*), 8.06 (tt, 1H, $^{3}J_{HH}$ = 7.6 Hz, $^{4}J_{HH}$ = 1.6 Hz, C4$_{pyridine}$−*H*), 7.64 (m, 4H, C5+8$_{aryl}$−*H*/ C3+5$_{pyridine}$−*H*), 7.34 (m, 4H, C3+4+10+11$_{aryl}$−*H*), 2.78 (s, 2H, B−*H*), 2.28 (s, 1H, B−*H*), 2.14 (s, 4H, B−*H*), 2.00 (s, 1H, B-*H*), 1.79 (s, 2H, B−*H*), 1.20 (s, 3H, C*H*$_3$) ppm.

**$^{11}B$ NMR** (160 MHz, $C_6D_6$): $\delta$ = 0.3 (s, 1B, B$_{ring}$), −1.9 (d, 1B, *B*12), −5.8 (d, 1B, *B*7), −8.4 (d, 4B), −9.4 (d, 2B), −10.6 (d, 2B) ppm.

**$^{13}C\{^{1}H\}$ NMR** (126 MHz, $C_6D_6$): $\delta$ = 149.68 (s, 2C, *C*1+12$_{aryl}$−B), 149.06 (s, 2C, *C*6+7$_{aryl}$), 147.33 (s, 2C, *C*2+6$_{pyridine}$), 141.52 (s, 1C, *C*4$_{pyridine}$), 133.56 (s, 2C, *C*2+11$_{aryl}$), 128.24 (s, 2C, *C*4+9$_{aryl}$), 126.73 (s, 2C, *C*3+10$_{aryl}$), 125.26 (s, 2C, *C*3+5$_{pyridine}$), 119.65 (s, 2C, *C*5+8$_{aryl}$), 80.36 (s, 1C, *C*$_{cage}$−B), 76.46 (s, 1C, *C*$_{cage}$−CH$_3$), 24.81 (s, 1C, C$_{cage}$−*C*H$_3$) ppm.

**Elemental analysis** (%) calcd. for $C_{20}H_{26}B_{11}N$: C 60.15, H 6.56, N 3.51; found: C 60.27, H 6.63, N 3.46.

**IR spectroscopy** [$\tilde{\nu}$]: 3066 cm$^{-1}$ (*C$_{aryl}$−H*, w), 2559 cm$^{-1}$ (*B−H*, s), 1619 cm$^{-1}$ (*C=C*, m), 1460 cm$^{-1}$ (*C=C*, s).

## 2.7 Synthesis of **2-DMAP**

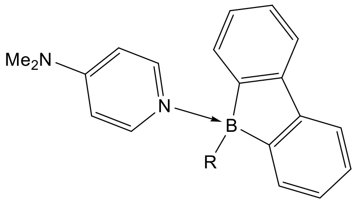


9-Carboranyl-9-borafluorene (**2**, 150 mg, 0.47 mmol) was dissolved in 3 mL toluene and a solution of 4-dimethylaminopyridine (57 mg, 0.47 mmol) in 1 mL of toluene was added. The reaction mixture was stirred for 1 h at room temperature. After a short time, the formation of a white precipitate was observed. Hexane (5 mL) was added to complete the precipitation. The solid was washed with hexane (5 x 1 mL) and dried *in vacuo*. Crystals suitable for X-ray diffraction analysis were obtained from a toluene solution of **2-DMAP** at –30 °C.

Yield: 198 mg (0.45 mmol, 95%) of a light-yellow solid.

**$^{1}H\{^{11}B\}$ NMR** (500 MHz, $d^8$-thf): $\delta$ = 9.33 (dm, $^{3}J_{HH}$ = 7.8 Hz, 2H, C2+6$_{DMAP}$–*H*), 8.63 (m, 2H, C2+11$_{aryl}$–*H*), 8.20 (m, 2H, C5+8$_{aryl}$–*H*), 7.83 (m, 4H, C3+4+9+10$_{aryl}$–*H*), 7.33 (dm, $^{3}J_{HH}$ = 7.8 Hz, 2H, C3+5$_{DMAP}$–*H*), 3.71 (s, 6H, N(C$H_3$)$_2$), 3.33 (s, 2H, B–*H*), 2.79 (s, 1H, B–*H*), 2.69 (s, 2H, B–*H*), 2.54 (s, 2H, B–*H*), 2.60 (s, 3H, B–*H*), 1.73 (s, 3H, C$H_3$) ppm.

**$^{11}B$ NMR** (160 MHz, $d^8$-thf): $\delta$ = −1.6 (s, 1B, B$_{ring}$), −2.4 (d, 1B, *B*12), −5.8 (d, 1B, *B*7), −8.7 (d, 2B), −9.7 (d, 4B), −10.3 (d, 2B) ppm.

**$^{13}C\{^{1}H\}$3 NMR** (126 MHz, $d^8$-thf): $\delta$ = 156.77 (s, 1C, *C*4$_{DMAP}$), 152.58 (s, 2C, *C*1+12$_{aryl}$–B), 150.40 (s, 2C, *C*2+6$_{DMAP}$), 134.81 (s, 2C, *C*2+11$_{aryl}$), 128.47 (s, 2C, *C*4+9$_{aryl}$), 127.13 (s, 2C, *C*3+10$_{aryl}$), 120.32 (s, 2C, *C*5+8$_{aryl}$), 107.11 (s, 2C, *C*3+5$_{DMAP}$), 84.20 (s, 1C, *C*$_{cage}$–B), 77.23 (s, 1C, *C*$_{cage}$–CH$_3$), 39.57 (s, 2C, N(*C*H$_3$)$_2$), 23.11 (s, 1C, C$_{cage}$–*C*H$_3$) ppm.

**Elemental analysis** (%) calcd. for $C_{22}H_{31}B_{11}N_2$: C 59.73, H 7.06, N 6.33; found: C 60.33, H 7.07, N 6.20.

**IR spectroscopy** [$\tilde{\nu}$]: 3046 cm$^{-1}$ (*C$_{aryl}$–H*, w), 2555 cm$^{-1}$ (*B–H*, s), 1644 cm$^{-1}$ (*C=C*, s), 1561 cm$^{-1}$ (*C=C*, s), 1430 cm$^{-1}$ (*C=C*, m).

## 2.8 Synthesis of **3**

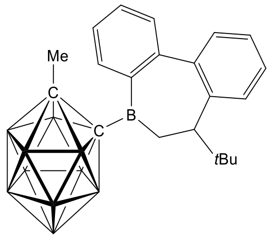


Dry neohexene (397 mg, 4.70 mmol) dissolved in 1 mL of toluene was added to a solution of 9-carboranyl-9-borafluorene (**2**, 150 mg, 0.47 mmol) in 3 mL of toluene and the reaction mixture was stirred for 16 h at room temperature. The reaction mixture was concentrated under vacuum and 5 mL of hexane was added to produce a white precipitate. The precipitate was isolated, washed with hexane (10 x 1 mL) and dried *in vacuo*. Crystals suitable for X-ray diffraction analysis were obtained by diffusing pentane into a benzene solution of **3**.

Yield: 48 mg (0.12 mmol, 25%) of a colorless solid.

**$^{1}H\{^{11}B\}$ NMR** (500 MHz, $C_6D_6$): $\delta$ = 7.46 (dd, 2H, $^{3}J_{HH}$ = 8.7 Hz, $^{3}J_{HH}$ = 8.0 Hz, C2+11$_{aryl}$−*H*), 7.21 (dm, 1H, $^{3}J_{HH}$ = 7.3 Hz, C5$_{aryl}$−*H*), 7.18 (dd, 1H, $^{3}J_{HH}$ = 7.6 Hz, $^{2}J_{HH}$ =1.3 Hz, C4$_{aryl}$−*H*), 7.03 (dd, 1H, $^{3}J_{HH}$ = 7.6 Hz, $^{4}J_{HH}$ =1.4 Hz, C3$_{aryl}$−*H*), 6.98 (m, 1H, C10$_{aryl}$−*H*), 6.95 (m, 2H, C8+9$_{aryl}$−*H*), 3.18 (s, 1H, B12−*H*), 3.03 (dd, 1H, $^{3}J_{HH}$ = 12.7 Hz, $^{4}J_{HH}$ = 2.9 Hz, C13$_{alkyl}$−*H*), 2.91 (s, 2H, B7−*H*/B−*H*), 2.88 (s, 1H, B−*H*), 2.60 (s, 2H, B−*H*), 2.53 (s, 1H, B−*H*), 2.50 (dd, 1H, $^{3}J_{HH}$ = 12.7 Hz, $^{3}J_{HH}$ = 18.6 Hz, C14$_{alkyl}$−*H*H), 2.33 (s, 2H, B−*H*), 2.20 (s, 1H, B−*H*), 1.84 (dd, 1H, $^{3}J_{HH}$ = 18.6 Hz, $^{4}J_{HH}$ = 2.9 Hz, C14$_{alkyl}$−*H*H), 0.92 (s, 9H, C(C$H_3$)$_3$), 0.79 (s,3H, C$H_3$) ppm.

**$^{11}B$ NMR** (160 MHz, $C_6D_6$): $\delta$ = 79.9 (s, 1B, B$_{ring}$), 2.0 (d, 1B, *B*12), −4.9 (d, 1B, *B*7), −7.1 (d, 2B), −7.9 (d, 2B), −8.8 (d, 2B), −10.0 (d, 2B) ppm.

**$^{13}C\{^{1}H\}$ NMR** (126 MHz, $C_6D_6$): $\delta$ = 144.95 (s, 1C, *C*6$_{aryl}$), 144.11 (s, 1C, *C*12$_{aryl}$), 141.67 (s,1C, *C*1$_{aryl}$-B), 141.10 (s, 1C, *C*7$_{aryl}$), 131.89 (s, 1C, *C*4$_{aryl}$), 130.83 (s, 1C, *C*8$_{aryl}$), 129.57 (s, 1C, *C*2$_{aryl}$), 128.47 (s, 1C, *C*5$_{aryl}$), 127.49 (s, 1C, *C*10$_{aryl}$), 126.56 (s, 1C, *C*9$_{aryl}$), 126.23 (s, 1C, *C*3$_{aryl}$), 125.98 (s, 1C, *C*11$_{aryl}$), 76.94 (s, 1C, *C*$_{cage}$−$CH_3$), 75.10 (s, 1C, *C*$_{cage}$−B), 48.16 (s, 1C, *C*13$_{alkyl}$), 44.96 (s, 1C, B−*C*14$_{alkyl}$), 32.95 (s, 1C, *C*($CH_3$), 29.11 (s, 3C, C(*C*$H_3$)$_3$), 23.28 (s, 1C, C$_{cage}$−*C*$H_3$) ppm.

**HRMS** (LIFDI): calculated for **3**: 404.3678; found: 404.3671.

**Elemental analysis** (%) calcd. for $C_{21}H_{33}B_{11}$: C 62.37, H 8.23; found: C 62.73, H 8.19.

**IR spectroscopy** [$\tilde{\nu}$]: 2958 $cm^{-1}$ (*C$_{alkyl}$−H*, m), 2578 $cm^{-1}$ (*B−H*, s), 1589 $cm^{-1}$ (*C=C*, m), 1468 $cm^{-1}$ (*C=C*, m).

## 2.9 Synthesis of **4**

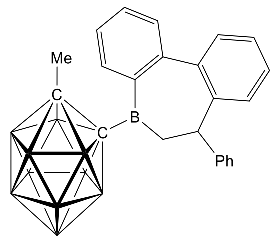


Dry styrene (490 mg, 4.70 mmol) dissolved in 1 mL toluene was added to a solution of 9-carboranyl-9-borafluorene (**2**, 150 mg, 0.47 mmol) in 3 mL of toluene and the reaction mixture was stirred for 16 h at room temperature. The mixture was concentrated under vacuum and 5 mL of hexane was added to produce a white precipitate. The precipitate was washed with hexane (10 x 1 mL) and dried *in vacuo*. Crystals suitable for X-ray diffraction analysis were obtained by diffusing hexane into a benzene solution of **4**.

Yield: 92 mg (0.23 mmol, 49%) of a colorless solid.

**$^{1}H\{^{11}B\}$ NMR** (500 MHz, $C_6D_6$): $\delta$ = 7.51 (d, 1H, $^{3}J_{HH}$ = 7.4 Hz, C2$_{aryl}$−*H*), 7.26 (m, 2H, C4+11$_{aryl}$−*H*), 7.16 (m, 2H, C5+8$_{aryl}$−*H*), 7.10 (m, 2H, C3+10$_{aryl}$−*H*), 7.04 (d, 2H, $^{3}J_{HH}$ = 7.3 Hz, C2/6$_{phenyl}$−*H*), 6.99 (dd, 1H, $^{3}J_{HH}$ = 7.6 Hz, $^{4}J_{HH}$ = 1.3 Hz, C9$_{aryl}$−*H*), 6.92 (dt, 1H, $^{3}J_{HH}$ = 7.6 Hz, $^{4}J_{HH}$ = 1.3 Hz, C3/5$_{phenyl}$−*H*), 6.82 (dt, 1H, $^{3}J_{HH}$ = 7.7 Hz, $^{4}J_{HH}$ = 1.2 Hz, C4$_{phenyl}$−*H*), 6.71 (d, 1H, $^{3}J_{HH}$ = 7.7 Hz, C3/5$_{phenyl}$−*H*), 4.35 (dd, 1H, $^{3}J_{HH}$ = 13.0 Hz, $^{4}J_{HH}$ = 3.0 Hz, C14$_{alkyl}$−*H*), 3.20 (s, 1H, B7−*H*), 2.94 (s, 2H, B12−*H*/B−*H*), 2.91 (s, 1H, B-*H*), 2.83 (dd, 1H, $^{3}J_{HH}$ = 13.0 Hz, $^{3}J_{HH}$ = 18.9 Hz, C13$_{alkyl}$−*H*), 2.58 (s, 2H, B−*H*), 2.55 (s, 2H, B−*H*), 2.35 (s, 2H, B−*H*), 2.26 (s, 2H, B−*H*), 1.72 (dd, 1H, $^{3}J_{HH}$ = 18.9 Hz, $^{4}J_{HH}$ = 3.0 Hz, C14$_{alkyl}$−*H*H), 0.84 (s, 3H, C$H_3$) ppm.

**$^{11}B$ NMR** (160 MHz, $C_6D_6$): $\delta$ = 79.2 (s, 1B, B$_{ring}$), 2.0 (d, 1B, *B*12), −4.8 (d, 1B, *B*7), −7.1 (d, 2B), −7.9 (d, 2B), −9.0 (d, 2B), −10.1 (d, 2B) ppm.

**$^{13}C\{^{1}H\}$ NMR** (126 MHz, $C_6D_6$): $\delta$ = 145.73 (s, 1C, *C*12$_{aryl}$), 144.42 (s, 1C, *C*6$_{aryl}$), 142.45 (s, 1C, *C*7$_{aryl}$), 141.31 (s,1C, *C*1$_{aryl}$-B), 139.53 (s, 1C, *C*1$_{phenyl}$), 132.04 (s, 1C, *C*4$_{aryl}$), 130.40 (s, 1C, *C*2$_{aryl}$), 129.77 (s, 1C, *C*9$_{aryl}$), 129.28 (s, 1C, *C*11$_{aryl}$), 129.14 (s, 2C, *C*2/6$_{phenyl}$), 128.71 (s, 2C, *C*3/5$_{phenyl}$), 128.12 (s, 1C, *C*4$_{phenyl}$), 127.06 (s, 1C, *C*5/8$_{aryl}$), 126.99 (s, 1C, *C*3$_{aryl}$), 126.63 (s, 1C, *C*5/8$_{aryl}$), 126.18 (s, 1C, *C*10$_{aryl}$), 76.90 (s, 1C, *C*$_{cage}$−$CH_3$), 75.05 (s, 1C, *C*$_{cage}$−B), 47.00 (s, 1C, B−*C*14$_{alkyl}$), 43.69 (s, 1C, *C*13$_{alkyl}$), 24.27 (s, 1C, C$_{cage}$−*C*$H_3$) ppm.

**HRMS** (LIFDI): calculated for **4**: 424.3365; found: 424.3360.

**Elemental analysis** (%) calcd. for $C_{23}H_{29}B_{11}$: C 65-09, H 6.89; found: C 65.11, H 6.88.

**IR spectroscopy** [$\tilde{\nu}$]: 3057 cm$^{-1}$ ($C_{aryl}$−*H*, w), 2579 cm$^{-1}$ (*B*−*H*, s), 1590 cm$^{-1}$ (*C*=*C*, m), 1423 cm$^{-1}$ (*C*=*C*, s).

## 2.10 Synthesis of **5**

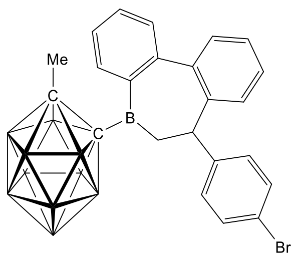


A solution of dry 4-bromostyrene (860 mg, 4.70 mmol) dissolved in 1 mL of toluene was added to a solution of 9-carboranyl-9-borafluorene (**2**, 150 mg, 0.47 mmol) in 3 mL of toluene and the reaction mixture was stirred for 72 h at room temperature. The reaction mixture was concentrated under vacuum and hexane (5 mL) was added to produce a white precipitate. The precipitate was isolated, washed with hexane (10 x 1 mL) and dried *in vacuo*. Crystals suitable for X-ray diffraction analysis were obtained by diffusing pentane into a benzene solution of **5**.

Yield: 187 mg (0.37 mmol, 79%) of a light yellow solid.

**$^{1}$H{$^{11}$B} NMR** (500 MHz, $C_6D_6$): $\delta$ = 7.50 (d, 1H, $^{3}J_{HH}$ = 7.6 Hz, C2$_{aryl}$−*H*), 7.29 (d, 2H, $^{3}J_{HH}$ = 7.8 Hz, C3+5$_{phenyl}$−*H*), 7.25 (m, 2H, C4+5$_{aryl}$−*H*), 7.10 (m, 1H, C3$_{aryl}$−*H*), 6.97 (m, 1H, C8$_{aryl}$−*H*), 6.92 (m, 1H, C9$_{aryl}$−*H*), 6.85 (m, 1H, C10$_{aryl}$−*H*), 6.64 (d, 2H, $^{3}J_{HH}$ = 7.7 Hz, C2+6$_{phenyl}$−*H*), 6.53 (d, 1H, $^{3}J_{HH}$ = 7.6 Hz, C11$_{aryl}$−*H*), 4.16 (d, 1H, $^{3}J_{HH}$ = 13.0 Hz, C14$_{alkyl}$−*H*H), 3.19 (s, 1H, B12−*H*), 2.93 (s, 2H, B7−*H* / B−*H*), 2.90 (s, 1H, B-*H*), 2.66 (dd, 1H, $^{3}J_{HH}$ = 12.9 Hz, $^{3}J_{HH}$ = 18.2 Hz, C13$_{alkyl}$−*H*), 2.57 (s, 2H, B-*H*), 2.52 (s, 1H, B-*H*), 2.34 (s, 2H, B-*H*), 2.20 (s, 1H, B-*H*), 1.59 (d, 1H, $^{3}J_{HH}$ = 18.5 Hz; C14$_{akyl}$−*H*H), 0.81 (s, 3H, C$H_3$) ppm.

**$^{11}$B NMR** (160 MHz, $C_6D_6$): $\delta$ = 79.6 (s, 1B, B$_{ring}$), 2.1 (d, 1B, *B*12), −4.7 (d, 1B, *B*7), −7.1 (d, 2B), −7.8 (d, 2B), −9.0 (d, 2B), −10.2 (d, 2B) ppm.

**$^{13}$C{$^{1}$H} NMR** (126 MHz, $C_6D_6$): $\delta$ = 145.02 (s, 1C, *C*12$_{aryl}$), 144.21 (s, 1C, *C*6$_{aryl}$), 141.43 (s, 1C, *C*1$_{phenyl}$), 141.19 (s, 1C, *C*1$_{aryl}$−B), 139.47 (s, 1C, *C*7$_{aryl}$), 132.14 (s, 1C, *C*4$_{aryl}$), 131.77 (s, 2C, *C*3+5$_{phenyl}$), 130.84 (s, 2C, *C*2+6$_{phenyl}$), 130.44 (s, 1C, *C*2$_{aryl}$), 129.82 (s, 1C, *C*8$_{aryl}$), 129.25 (s, 1C, *C*5$_{aryl}$), 128.19 (s, 1C, *C*10$_{aryl}$), 127.25 (s, 1C, *C*9$_{aryl}$), 126.34 (s, 1C, *C*11$_{aryl}$), 126.27 (s, 1C, *C*3$_{aryl}$), 120.97 (s, 1C, *C*4$_{phenyl}$−Br), 76.92 (s, 1C, *C*$_{cage}$−CH$_3$), 74.89 (s, 1C, *C*$_{cage}$−B), 46.68 (s, 1C, B−*C*14$_{alkyl}$), 43.08 (s, 1C, *C*13$_{alkyl}$), 24.24 (s, 1C, C$_{cage}$−*C*H$_3$) ppm.

**HRMS** (LIFDI): calculated for **5**: 502.2471; found: 502.2446.

**Elemental analysis** (%) calcd. for $C_{23}H_{28}B_{11}Br$: C 54.89, H 5.61; found: C 57.27, H 5.46.*

**IR spectroscopy** [$\tilde{\nu}$]: 3055 $cm^{-1}$ ($C_{aryl}$−*H*, w), 2570 $cm^{-1}$ (*B*−*H*, s), 1592 $cm^{-1}$ (*C*=*C*, m), 1483 $cm^{-1}$ (*C*=*C*, s), 1434 $cm^{-1}$ (*C*=*C*, s).

**Note: Despite several attempts with NMR-clean material this is the best result so far.*

## 2.11 Synthesis of **5-*t*BuCN**

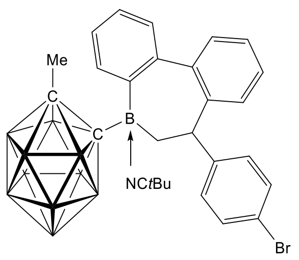


A solution of pivalonitrile (17 mg, 0.20 mmol) in 1 mL of toluene was added with stirring to a solution of **5** (100 mg, 0.20 mmol) in 4 mL of toluene. Addition of pentane (5 mL) resulted in the precipitation of a white solid, which was washed with hexane (5 x 2 mL) and dried *in vacuo*. Crystals suitable for X-ray diffraction analysis were obtained by diffusing pentane into a benzene solution of **5-*t*BuCN**.

Yield: 103 mg (0.18 mmol, 88%) of a colorless solid.

**$^{1}H\{^{11}B\}$ NMR** (500 MHz, $C_6D_6$): $\delta$ = 7.72 (dd, 1H, $^{3}J_{HH}$ = 7.5 Hz, $^{4}J_{HH}$ = 1.5 Hz, $C2_{aryl}$−*H*), 7.38 (dd, 1H, $^{3}J_{HH}$ = 7.6 Hz, $^{4}J_{HH}$ = 1.5 Hz, $C5_{aryl}$−*H*), 7.32 (d, 2H, $^{3}J_{HH}$ = 8.4 Hz, $C3{+}5_{phenyl}$−*H*), 7.31 (dd, 1H, $^{3}J_{HH}$ = 7.5 Hz, $^{4}J_{HH}$ = 1.7 Hz, $C4_{aryl}$−*H*), 7.26 (dd, 1H, $^{3}J_{HH}$ = 7.3 Hz, $^{4}J_{HH}$ = 1.6 Hz, $C3_{aryl}$−*H*), 7.22 (dd, 1H, $^{3}J_{HH}$ = 7.6 Hz, $^{4}J_{HH}$ = 1.3 Hz, $C8_{aryl}$−*H*), 7.04 (dd, 1H, $^{3}J_{HH}$ = 7.5 Hz, $^{4}J_{HH}$ = 1.3 Hz, $C9_{aryl}$−*H*), 6.96 (dd, 1H, $^{3}J_{HH}$ = 7.6 Hz, $^{4}J_{HH}$ = 1.4 Hz, $C10_{aryl}$−*H*), 6.84 (d, 2H, $^{3}J_{HH}$ = 8.4 Hz, $C2{+}6_{phenyl}$−*H*), 6.61 (d, 1H, $^{3}J_{HH}$ = 7.8 Hz, $C11_{aryl}$−*H*), 4.26 (d, 1H, $^{3}J_{HH}$ = 12.0 Hz, $C14_{alkyl}$−*H*H), 3.15 (s, 1H, B12−*H*), 2,98 (s, 1H, B7−*H*), 2.87 (s, 2H, B−*H*), 2.70 (s, 1H, B−*H*), 2.48 (s, 4H, B−*H*), 2.37 (s, 1H, B−*H*), 2.04 (dd, 1H, $^{3}J_{HH}$ = 12.0 Hz, $^{3}J_{HH}$ = 16.0 Hz, $C13_{alkyl}$−*H*), 1.61 (dd, 1H, $^{3}J_{HH}$ = 16.0 Hz, $^{4}J_{HH}$ = 1.3 Hz, $C14_{alkyl}$−*H*H), 1.10 (s,3H, $CH_3$), 0.52 (s, 9H, $C(CH_3)_3$) ppm.

**$^{11}B$ NMR** (160 MHz, $C_6D_6$): $\delta$ = 21.5 (s, 1B, $B_{ring}$), −0.3 (d, 1B, *B*12), −4.4 (d, 1B, *B*7), −7.9 (d, 2B), −8.8 (d, 4B), −9.8 (d, 2B) ppm.

$^{13}C\{^1H\}$ **NMR** (126 MHz, $C_6D_6$): $\delta$ = 145.79 (s, 1C, $C12_{aryl}$), 145.33 (s, 1C, $C6_{aryl}$), 144.44 (s, 1C, $C1_{phenyl}$), 143.04 (s, 1C, $C1_{aryl}$–B), 140.65 (s, 1C, $C7_{aryl}$), 133.86 (s, 1C, $C2_{aryl}$), 131.74 (s, 2C, $C3{+}5_{phenyl}$), 131.72 (s, 1C, $C5_{aryl}$), 131.19 (s, 2C, $C2{+}6_{phenyl}$), 130.82 (s, 1C, $C8_{aryl}$), 129.31 (s, 1C, $C4_{aryl}$), 127.84 (s, 1C, $C10_{aryl}$), 126.81 (s, 1C, $C9_{aryl}$), 126.32 (s, 1C, $C3_{aryl}$), 125.14 (s, 1C, $C11_{aryl}$), 121.56 (s, 1C, *C*N), 120.61 ($C12_{aryl}$), 79.53 (s, 1C, $C_{cage}$–B), 75.85 (s, 1C, $C_{cage}$-$CH_3$), 44.68 (s, 1C, $C13_{alkyl}$), 40.86 (s; 1C, B–$C14_{alkyl}$), 28.39 (s, 1C, $C(CH_3)_3$), 26.32 (s, 3C, $C(CH_3)_3$), 24.20 (s, 1C, $C_{cage}$–$CH_3$) ppm.

**HRMS** (LIFDI): calculated for [**5** – NC-*t*Bu]: 502.2471; found: 502.2465.

**Elemental analysis** (%) calcd. for $C_{28}H_{37}B_{11}NBr$: 57.35, H 6.36 N 2.39; found: C 57.66, H 6.40, N 2.30.

**IR spectroscopy** [$\tilde{\nu}$]: 3053 $cm^{-1}$ ($C_{aryl}$–*H*, w), 2973 $cm^{-1}$ ($C_{alkyl}$–*H*, w), 2572 $cm^{-1}$ (*B*–*H*, s), 2323 $cm^{-1}$ (*C≡N*, m), 1593 $cm^{-1}$ (*C=C*, w), 1440 $cm^{-1}$ (*C=C*, m).

## 2.12 Synthesis of **6-*t*BuCN**

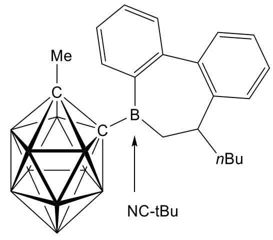


A solution of dry 1-hexene (395 mg, 4.70 mmol) dissolved in 1 mL of toluene was added to a solution of 9-(1-Me-2-*B*-*ortho*-$C_2B_{10}H_{12}$)-9-hydro-9-borafluorene (**2**, 150 mg, 0.47 mmol) in 5 mL toluene and the reaction mixture was stirred for 16 h at room temperature. Full conversion was confirmed by $^{11}B$ NMR spectroscopy (δ = 78 ppm). To the reaction mixture was added with stirring a solution of pivalonitrile (138 mg, 0.94 mmol) in 1 mL of toluene. The solution was concentrated and precipitated with pentane (10 mL), affording a white solid, which was washed with hexane (10 x 1 mL) and dried *in vacuo*. Crystals suitable for X-ray diffraction analysis were obtained by diffusing pentane into a benzene solution of **6-*t*BuCN**.

Yield: 261 mg (0.54 mmol, 57%) of a colorless solid.

$^1H\{^{11}B\}$ **NMR** (500 MHz, $C_6D_6$): $\delta$ = 7.65 (dd, 1H, $^3J_{HH}$ = 7.5 Hz, $^4J_{HH}$ = 1.8 Hz, $C2_{aryl}$–*H*), 7.26 (dd, 2H, $^3J_{HH}$ = 7.6 Hz, $^4J_{HH}$ = 1.5 Hz, $C5_{aryl}$–*H*), 7.22 (dt, 1H, $^3J_{HH}$ = 7.3 Hz, $^4J_{HH}$ = 1.6 Hz, $C4_{aryl}$–*H*), 7.19 (dt, 1H, $^3J_{HH}$ = 7.3 Hz, $^4J_{HH}$ = 1.7 Hz, $C3_{aryl}$–*H*), 7.14 (m, 3H, $C8{+}9{+}10_{aryl}$–*H*), 7.06 (m, 1H, $C9_{aryl}$–*H*), 3.11 (s, 1H, B12–*H*), 2.93 (s, 1H, B7–*H*), 2.85 (s, 1H, B–*H*), 2.82 (s, 1H, B–*H*), 2.81 (m, 1H,

$C13_{alkyl}$-*H*), 2.67 (s, 1H, B−*H*), 2.45 (s, 2H, B−*H*), 2.40 (s, 3H, B−*H*), 1.75 (m, 1H, $C1_{alkyl}$−*H*), 1.53 (m, 1H, $C14_{alkyl}$−*H*H), 1.50 (m, 1H, $C1_{alkyl}$−*H*), 1.43 (dd, 1H, $^3J_{HH}$ = 16.8 Hz, $^4J_{HH}$ = 2.3 Hz, $C14_{alkyl}$−*H*H), 1.23 (m, 2H, $C3_{alkyl}$−*H*), 1.16 (m, 1H, $C2_{alkyl}$−*H*), 1.01 (m, 1H, $C2_{alkyl}$−*H*), 0.98 (s, 3H, $CH_3$), 0.80 (t, 3H, $^3J_{HH}$ = 7.2 Hz, $C4_{alkyl}$−*H*), 0.67 (s, 9H, $C(CH_3)_3$) ppm.

**$^{11}$B NMR** (160 MHz, $C_6D_6$): $\delta$ = 32.6 (s, 1B, $B_{ring}$), 0.0 (d, 1B, *B*12), −4.7 (d, 1B, *B*7), −8.5 (d, 2B), −8.7 (d, 4B), −9.8 (d, 2B) ppm.

**$^{13}$C{$^1$H} NMR** (126 MHz, $C_6D_6$): $\delta$ = 145.99 (s, 1C, $C12_{aryl}$), 145.34 (s, 1C, $C6_{aryl}$), 143.23 (s, 1C, $C1_{aryl}$-B), 141.30 (s, 1C, $C7_{aryl}$), 132.98 (s, 1C, $C2_{aryl}$), 130.96 (s, 1C, $C5_{aryl}$), 130.78 (s, 1C, $C8/10/11_{aryl}$), 129.52 (s, 1C, $C4_{aryl}$), 127.95 (s, 1C, $C8/10/11_{aryl}$), 126.40 (s, 1C, $C9_{aryl}$), 126.08 (s, 1C, $C3_{aryl}$), 123.25 (s, 1C, $C8/10/11_{aryl}$), 121.89 (s, 1C, *C*N), 78.78 (s, 1C, $C_{cage}$−B), 75.97 (s, 1C, $C_{cage}$−$CH_3$), 43.59 (s, 1C, B−$C14_{alkyl}$), 38.95 (s, 1C, $C13_{alkyl}$), 34.53 (s, 1C, $C1_{alkyl}$), 30.90 (s, 1C, $C2_{alkyl}$), 28.28 (s, 1C, $C(CH_3)_3$), 26.82 (s, 3C, $C(CH_3)_3$), 24.20 (s, 1C, $C_{cage}$−$CH_3$), 23.51 (s, 1C, $C3_{alkyl}$), 14.44 (s, 1C, $C4_{alkyl}$) ppm.

**HRMS** (LIFDI): calculated for [**6** – NC-*t*Bu]: 404.3678; found: 404.3669.

**Elemental analysis** (%) calcd. for $C_{26}H_{42}B_{11}N$: C 64.05, H 8.68, N 2.89; found: C 64.33, H 8.62, N 2.87.

**IR-spectroscopy** [$\tilde{\nu}$]: 3053 cm$^{-1}$ ($C_{aryl}$−*H*, w), 2926 cm$^{-1}$ ($C_{alkyl}$−*H*, w), 2557 cm$^{-1}$ (*B*−*H*, s), 2319 cm$^{-1}$ (*C*≡*N*, m), 1593 cm$^{-1}$ (*C*=*C*, w), 1440 cm$^{-1}$ (*C*=*C*, m).

*Note: In solution **6-tBuCN** is in equilibrium with the base-free insertion product **6**.*

## 2.13 Synthesis of **7-*t*BuCN**

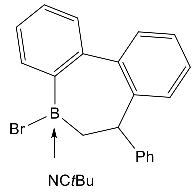


9-Bromo-9-borafluorene (**1**, 400 mg, 1.65 mmol) was dissolved in 10 mL of benzene, and to this mixture was added styrene (1.60 g, 15.4 mmol). The reaction mixture was stirred at 80 °C for 21 days. The completeness of the insertion was indicated by $^{11}$B NMR spectroscopy (δ = 73.8 ppm). Pivalonitrile (138 mg, 1.65 mmol) was added and the reaction mixture was concentrated to ca. 5 mL. Upon addition of 10 mL of pentane a white precipitate formed, which was washed with

hexane (10 x 1 mL) and dried *in vacuo*. Crystals suitable for X-ray diffraction analysis were obtained from a mixture of toluene/pentane of **7-*t*BuCN** at −30 °C.

Yield: 451 mg (1.05 mmol, 64%) of a colorless solid.

**$^{1}$H{$^{11}$B} NMR** (500 MHz, $C_6D_6$): *δ* = 8.67 (m, 1H, C2$_{aryl}$−*H*), 7.50 (m, 1H, C5$_{aryl}$−*H*), 7.38 (m, 2H, C3+4$_{aryl}$−*H*), 7.27 (d, 1H, $^{3}J_{HH}$ = 7.3 Hz, C8$_{aryl}$−*H*), 7.19 (m, 2H, C2+6$_{phenyl}$−*H*), 7.14 (m, 2H, C3+5$_{phenyl}$−*H*), 7.08 (m, 1H, C4$_{phenyl}$−*H*), 6,94 (t, 1H, $^{3}J_{HH}$ = 7.2 Hz, C9$_{aryl}$−*H*), 6.87 (m, 1H, C11$_{aryl}$−*H*), 6.83 (m, 1H, C10$_{aryl}$−*H*), 4.40 (dd, 1H, $^{3}J_{HH}$ = 13.2 Hz, $^{4}J_{HH}$ = 4.4 Hz, C13$_{alkyl}$−*H*), 2.43 (dd, 1H, $^{3}J_{HH}$ = 14.7 Hz, $^{4}J_{HH}$ = 4.6 Hz, C14$_{alkyl}$−*H*H), 2.31 (dd, 1H, $^{3}J_{HH}$ = 14.7 Hz, $^{3}J_{HH}$ = 13.2 Hz, C14$_{alkyl}$−*H*H), 0.28 (s, 9H, C(C*H*$_3$)$_3$) ppm.

**$^{11}$B NMR** (160 MHz, $C_6D_6$): *δ* = 8.8 (s, 1B, B$_{ring}$) ppm.

**$^{13}$C{$^{1}$H} NMR** (126 MHz, $C_6D_6$): *δ* = 146.03 (s, 1C, *C*12$_{aryl}$), 145.02 (s, 1C, *C*1$_{phenyl}$), 144.18 (s, 1C, *C*6$_{aryl}$), 143.74 (s, 1C, *C*7$_{aryl}$), 142.12 (s, 1C, *C*1$_{aryl}$−B), 135.04 (s, 1C, *C*2$_{aryl}$), 129.50 (s, 2C, *C*2+6$_{phenyl}$), 129.27 (s, 1C, *C*8$_{aryl}$), 128.98 (s, 1C, *C*5$_{aryl}$), 128.96 (s, 1C, *C*3/4$_{aryl}$), 128.38 (s, 1C, *C*3+5$_{phenyl}$), 127.80 (s, 1C, *C*3/4$_{aryl}$), 127.58 (s, 1C, *C*11$_{aryl}$), 127.08 (s, 1C, *C*10$_{aryl}$), 126.34 (s, 1C, *C*4$_{phenyl}$), 126.13 (s, 1C, *C*9$_{aryl}$), 120.15 (s, 1C, *C*N), 46.57 (s, 1C, *C*13$_{alkyl}$), 40.48 (s, 1C, B−*C*14$_{alkyl}$), 28.16 (s, 1C, *C*(CH$_3$)$_3$), 26.13 (s, 3C, C(*C*H$_3$)$_3$) ppm.

**HRMS** (LIFDI): calculated for **7-*t*BuCN**: 429.1263; found: 429.1253; calculated for [**7** – Br – NC-*t*Bu]: 267.1345; found: 267.1335.

**Elemental analysis** (%) calcd. for $C_{25}H_{25}BBrN$: C 69.80, H 5.86, N 3.26; found: C 69.09, H 7.65, N 2.75.

**IR spectroscopy** [$\tilde{\nu}$]: 3046 cm$^{-1}$ (*C$_{aryl}$−H*, w), 2915 cm$^{-1}$ (*C$_{alkyl}$−H*, m), 2318 cm$^{-1}$ (*C≡N*, m), 1595 cm$^{-1}$ (*C=C*, w), 1432 cm$^{-1}$ (*C=C*, s).

## 2.14 Synthesis of **8-*t*BuCN**

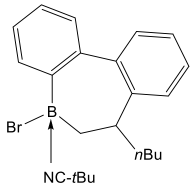


9-Bromo-9-borafluorene (**1Br**, 400 mg, 1.65 mmol) was dissolved in 10 mL of benzene and to this mixture 1-hexene (858 mg, 10.2 mmol) was added. The reaction mixture was stirred at 80 °C for 4 d. The completeness of the insertion reaction was confirmed by $^{11}$B NMR spectroscopy (δ = 74.1 ppm). Pivalonitrile (138 mg, 1.65 mmol) was added and the reaction mixture was

concentrated to ca. 5 mL. Upon addition of 10 mL of pentane a white precipitate formed, which was washed with hexane (10 × 1 mL) and dried *in vacuo*. Crystals suitable for X-ray diffraction analysis were obtained from a mixture of toluene/pentane of **8-*t*BuCN** at −30 °C.

Yield: 383 mg (0.93 mmol, 57%) of a colorless solid.

**$^{1}$H{$^{11}$B} NMR** (500 MHz, $C_6D_6$): $\delta$ = 8.60 (dd, 1H, $^{3}J_{HH}$ = 5.7 Hz, $^{4}J_{HH}$ = 3.2 Hz, $C2_{aryl}$−*H*), 7.41 (dd, 1H, $^{3}J_{HH}$ = 5.5 Hz, $^{4}J_{HH}$ = 3.2 Hz, $C5_{aryl}$−*H*), 7.33 (dd, 2H, $^{3}J_{HH}$ = 5.5 Hz, $^{4}J_{HH}$ = 3.4 Hz, $C3/4_{aryl}$−*H*), 7.25 (m, 2H, $C9/10_{aryl}$−*H*), 7.09 (dt, 1H, $^{3}J_{HH}$ = 7.4 Hz, $^{4}J_{HH}$ = 1.3 Hz, $C8_{aryl}$−*H*), 7.03 (dt, 1H, $^{3}J_{HH}$ = 7.3 Hz, $^{4}J_{HH}$ = 1.2 Hz, $C11_{aryl}$−*H*), 2.88 (m, 1H, $C13_{alkyl}$−*H*), 2.29 (dd, 1H, $^{3}J_{HH}$ = 15.4 Hz, $^{4}J_{HH}$ = 4.7 Hz, $C14_{Alkyl}$−*H*H), 1.77 (m, 1H, $C1_{alkyl}$−*H*), 1.57 (m, 1H, $C1_{alkyl}$−*H*), 1.55 (dd, 1H, $^{3}J_{HH}$ = 15.4 Hz, $^{3}J_{HH}$ = 12.7 Hz, $C14_{alkyl}$−*H*H), 1.18 (m, 2H, $C3_{alkyl}$−*H*), 1.13 (m, 2H, $C2_{alkyl}$−*H*), 0.77 (t, 3H, $^{3}J_{HH}$ = 7.0 Hz, $C4_{alkyl}$−*H*), 0.34 (s, 9H, C($CH_3$)$_3$) ppm.

**$^{11}$B NMR** (160 MHz, $C_6D_6$): $\delta$ = 8.4 (s, 1B, $B_{ring}$) ppm.

**$^{13}$C{$^{1}$H} NMR** (126 MHz, $C_6D_6$): $\delta$ = 145.19 (s, 1C, $C12_{aryl}$), 144.56 (s, 1C, $C6_{aryl}$), 144.32 (s, 1C, $C7_{aryl}$), 142.31 (s, 1C, $C1_{aryl}$−B), 134.78 (s, 1C, $C2_{aryl}$), 129.86 (s, 1C, $C9+10_{aryl}$), 129.35 (s, 1C, $C4_{aryl}$), 128.82 (s, 1C, $C5_{aryl}$), 127.58 (s, 1C, $C3_{aryl}$), 127.15 (s, 1C, $C8_{aryl}$), 125.98 (s, 1C, $C11_{aryl}$), 124.77 (s, 1C, $C9+10_{aryl}$), 120.64 (s, 1C, *C*N), 43.99 (s; 1C, B-$C14_{alkyl}$), 40.46 (s, 1C, $C13_{alkyl}$), 35.23 (s, 1C, $C1_{alkyl}$), 30.34 (s, 1C, $C2_{alkyl}$), 28.08 (s, 1C, *C*($CH_3$)$_3$), 26.34 (s, 3C, C($CH_3$)$_3$), 23.48 (s, 1C, $C3_{alkyl}$), 14.28 (s, 1C, $C4_{alkyl}$) ppm.

**Elemental analysis** (%) calcd. for $C_{23}H_{29}BBrN$: C 67.34, H 7.13, N 3.41; found: C 67.27, H 7.07, N 3.09.

**IR spectroscopy** [$\tilde{\nu}$]: 3053 cm$^{-1}$ ($C_{aryl}$−*H*, w), 2922 cm$^{-1}$ ($C_{alkyl}$−*H*, m), 2316 cm$^{-1}$ (*C≡N*, m), 1593 cm$^{-1}$ (*C=C*, w), 1431 cm$^{-1}$ (*C=C*, s).

## 2.15 Synthesis of **9-DMAP**

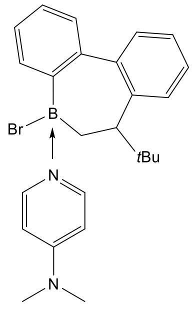


3,3-Dimethylbut-1-ene (1.75 g, 20.8 mmol) was added to a solution of 9-bromo-9-borafluorene (**1**, 400 mg, 1.65 mmol) in 10 mL of benzene and the reaction mixture was stirred at 80 °C for 21

days. The completeness of the insertion was monitored by $^{11}$B NMR spectroscopy (δ = 74.1 ppm). 4-Dimethylaminopyridine (202 mg, 1.65 mmol) was added and the reaction mixture was concentrated to ca. 5 mL. Upon addition of 10 mL of pentane a white precipitate formed, which was washed with hexane (10 × 1 mL) and dried *in vacuo*. Crystals suitable for X-ray diffraction analysis were obtained by diffusing hexane into a benzene solution of **9-DMAP**.

Yield: 244 mg (0.54 mmol, 33%) of a colorless solid.

Two diastereomers can be observed in solution in a ratio of ca. 4 to 1. The following resonances were assigned to the main isomer.

**$^1$H{$^{11}$B} NMR** (500 MHz, $C_6D_6$): $\delta$ = 8.20 (m, 1H, C2$_{aryl}$–*H*), 7.59 (d, 2H, $^3J_{HH}$ = 6.5 Hz, C2/6$_{DMAP}$–*H*), 7.49 (s, 1H, $^3J_{HH}$ = 7.8 Hz, C11$_{aryl}$–*H*), 7.35 (m, 2H, C3/4$_{aryl}$–*H*), 7.17 (m, 1H, C5$_{aryl}$–*H*), 6.96 (dt, 1H, $^3J_{HH}$ = 7.7 Hz, $^4J_{HH}$ = 1.4 Hz, C10$_{aryl}$–*H*), 6.79 (t, 1H, $^3J_{HH}$ = 7.5 Hz, C9$_{aryl}$–*H*), 6.69 (d, 1H, $^3J_{HH}$ = 7.5 Hz, C8$_{aryl}$–*H*), 6.11 (d, 2H, $^3J_{HH}$ = 7.4 Hz, C3/5$_{DMAP}$–*H*), 2.96 (s, 6H, N(C*H*$_3$)$_3$), 2.58 (dd, 1H, $^3J_{HH}$ = 12.7 Hz, $^4J_{HH}$ = 5.6 Hz, C13$_{alkyl}$–*H*), 1.89 (m, 2H, C14$_{alkyl}$–*H*$_2$), 1.11 (s, 9H, C(C*H*$_3$)$_3$) ppm.

**$^{11}$B NMR** (160 MHz, $C_6D_6$): $\delta$ = 8.4 (s, 1B, B$_{ring}$) ppm.

**$^{13}$C{$^1$H} NMR** (126 MHz, $C_6D_6$): $\delta$ = 154.99 (s, 1C, *C*4$_{DMAP}$), 146.93 (s, 1C, *C*1$_{aryl}$–B), 144.62 (s, 1C, *C*6$_{aryl}$), 144.34 (s, 1C, *C*7$_{aryl}$), 143.66 (s, 2C, *C*2+6$_{DMAP}$), 143.39 (s, 1C, *C*12$_{aryl}$), 134.19 (s, 1C, *C*2$_{aryl}$), 129.32 (s, 1C, *C*8$_{aryl}$), 127.82 (s, 1C, *C*5$_{aryl}$), 127.15 (s, C, *C*3/4$_{aryl}$), 126.42 (s, 1C, *C*3/4$_{aryl}$), 125.81 (s, 1C, *C*10$_{aryl}$), 125.71 (s, 1C, *C*11$_{aryl}$), 124,90 (s, 1C, *C*9$_{aryl}$), 105.37 (s, 2C, *C*3+5$_{DMAP}$), 50.64 (s, 1C, *C*13$_{alkyl}$), 39.38 (s, 2C, N(*C*H$_3$)$_2$), 31.72 (s, 1C, B–*C14*$_{alkyl}$), 29.37 (s, 1C, *C*(CH$_3$)$_3$), 26.13 (s, 3C, C(*C*H$_3$)$_3$) ppm.

**HRMS** (LIFDI): calculated for [**9-DMAP** – Br]: 369.2502; found: 369.2484.

**Elemental analysis** (%) calcd. for $C_{25}H_{30}BBrN_2$: C 66.84, H 6.73, N 6.24; found: C 68.21, H 7.00, N 5.79.

**IR spectroscopy** [$\tilde{\nu}$]: 3048 cm$^{-1}$ (*C$_{aryl}$–H*, w), 2930 cm$^{-1}$ (*C$_{alkyl}$–H*, m), 1631 cm$^{-1}$ (*C*=*C*, s), 1561 cm$^{-1}$ (*C*=*C*, w), 1430 cm$^{-1}$ (*C*=*C*, m).

**Table S1.** Assessment of the Lewis acidity of the 9-borafluorenes **1Br**, **1Ph**, and **2** with the Gutmann-Beckett method in $CD_2Cl_2$.

| Compound | Lewis base | $^{11}B$ NMR (ppm) | $^{31}P$ NMR (ppm) | $\Delta^{31}P$ NMR (ppm) | AN |
|---|---|---|---|---|---|
| **1Br** | $OPEt_3$ | 7.2 | 78.9 | 37.9 | 83.8 |
| **2** | $OPEt_3$ | 2.3 | 78.8 | 37.8 | 83.5 |
| **1Ph** | $OPEt_3$ | 5.0 | 76.4 | 35.4 | 78.2 |

# 3. NMR spectra

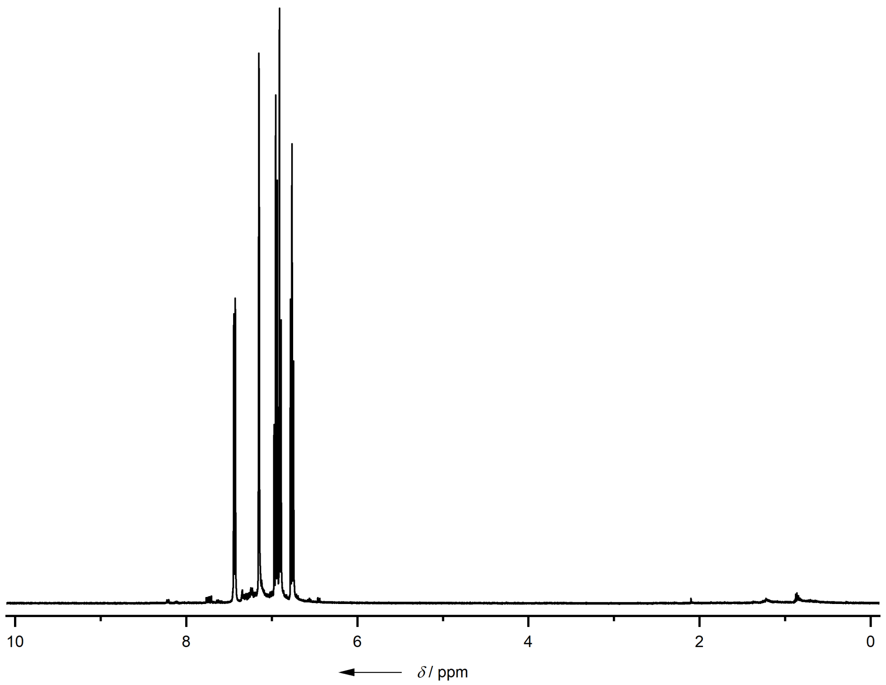


**Figure S1.** $^{1}H$ NMR spectrum of **1** in $C_6D_6$.

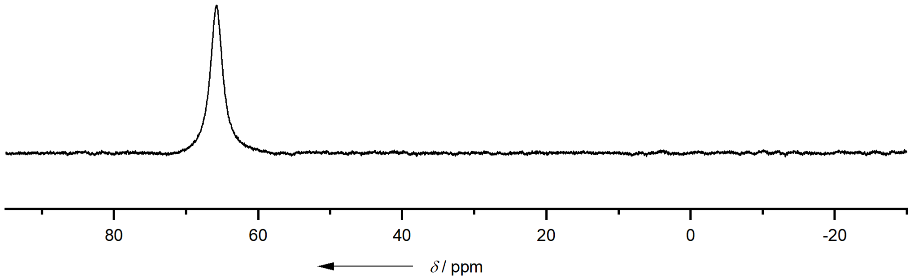


**Figure S2.** $^{11}B\{^{1}H\}$ NMR spectrum of **1** in $C_6D_6$.

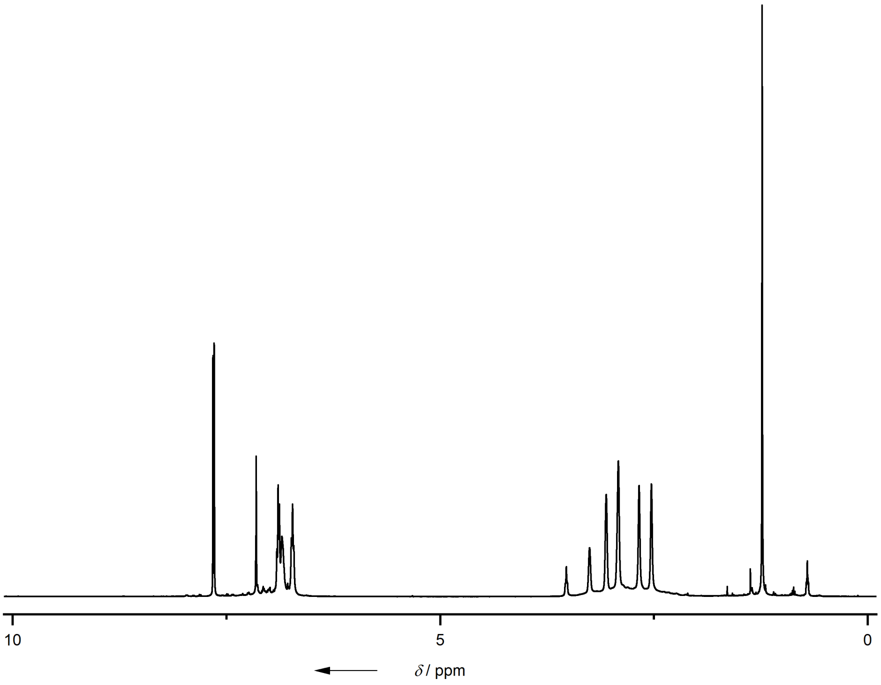


Figure S3. $^{1}$H NMR spectrum of **2** in $C_6D_6$.

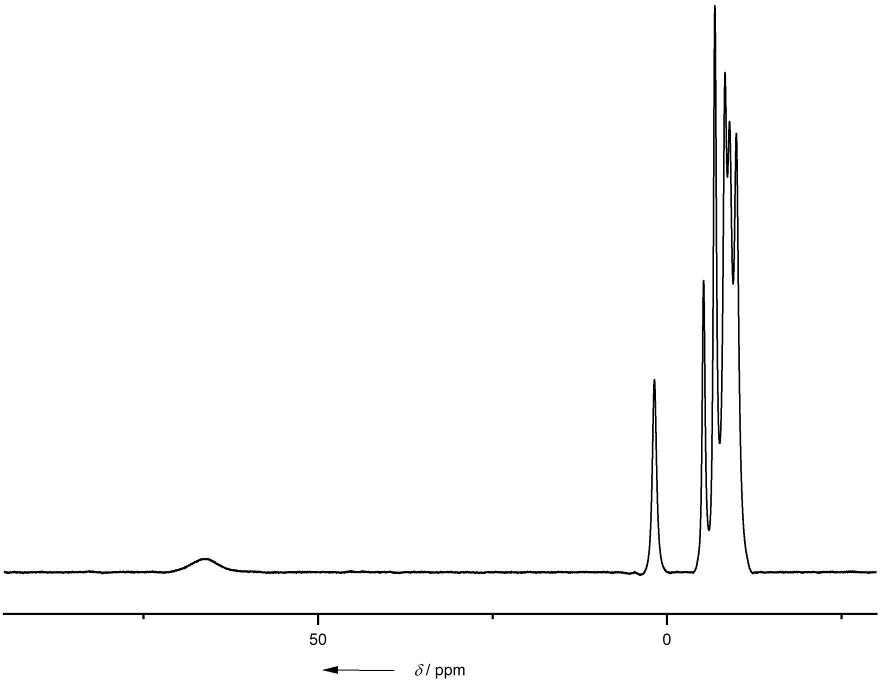


Figure S4. $^{11}$B{$^{1}$H} NMR spectrum of **2** in $C_6D_6$.

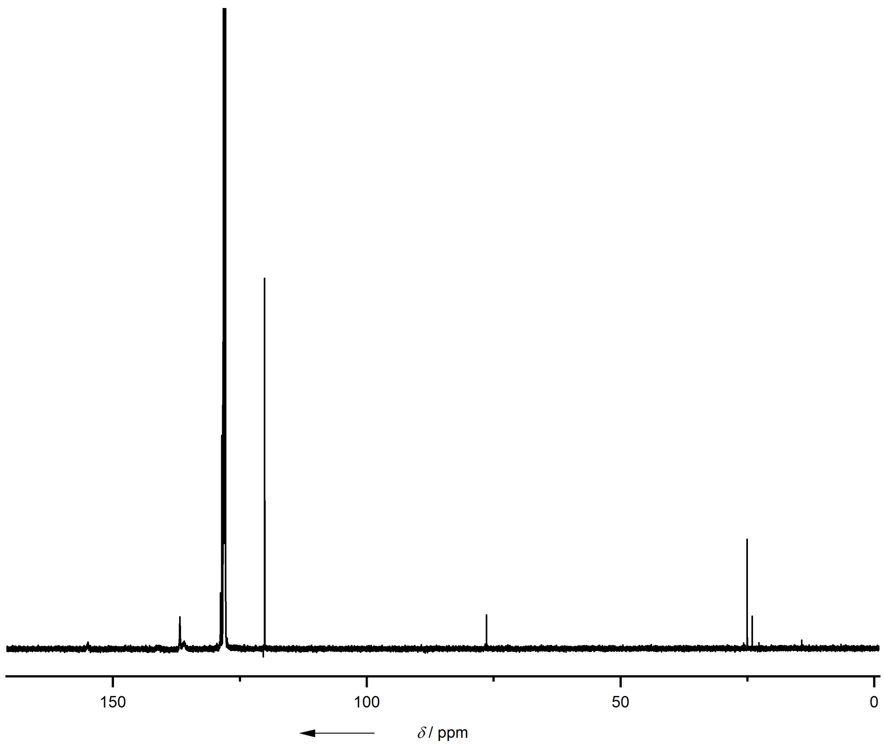


**Figure S5.** $^{13}C$ NMR spectrum of **2** in $C_6D_6$.

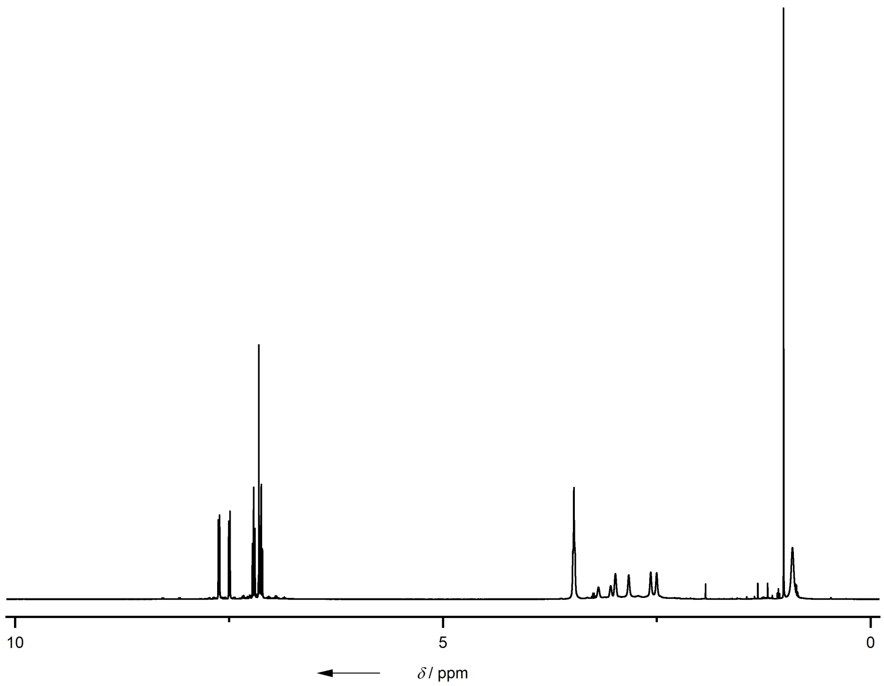


**Figure S6.** $^{1}H$ NMR spectrum of **2-thf** in $C_6D_6$.

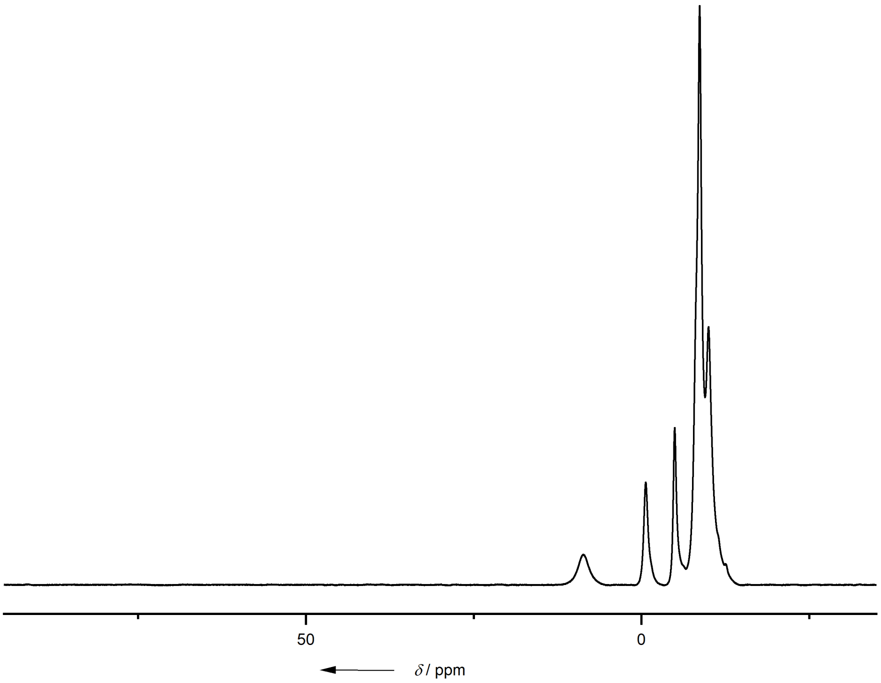


**Figure S7.** $^{11}B\{^1H\}$ NMR spectrum of **2-thf** in $C_6D_6$.

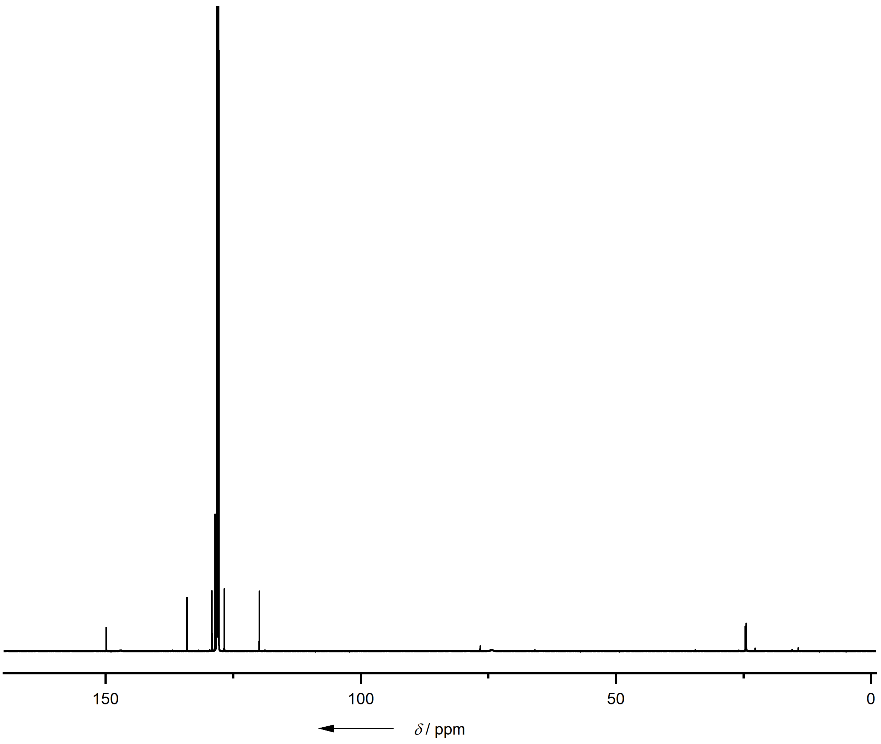


**Figure S8.** $^{13}C$ NMR spectrum of **2-thf** in $C_6D_6$.

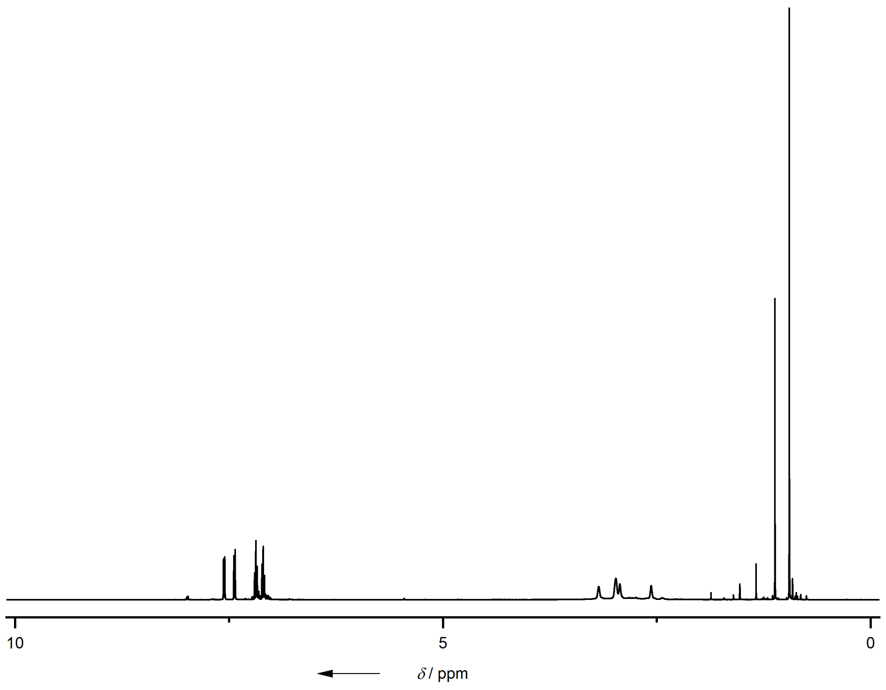


**Figure S9.** $^{1}H$ NMR spectrum of **2-acetone** in $C_6D_6$.

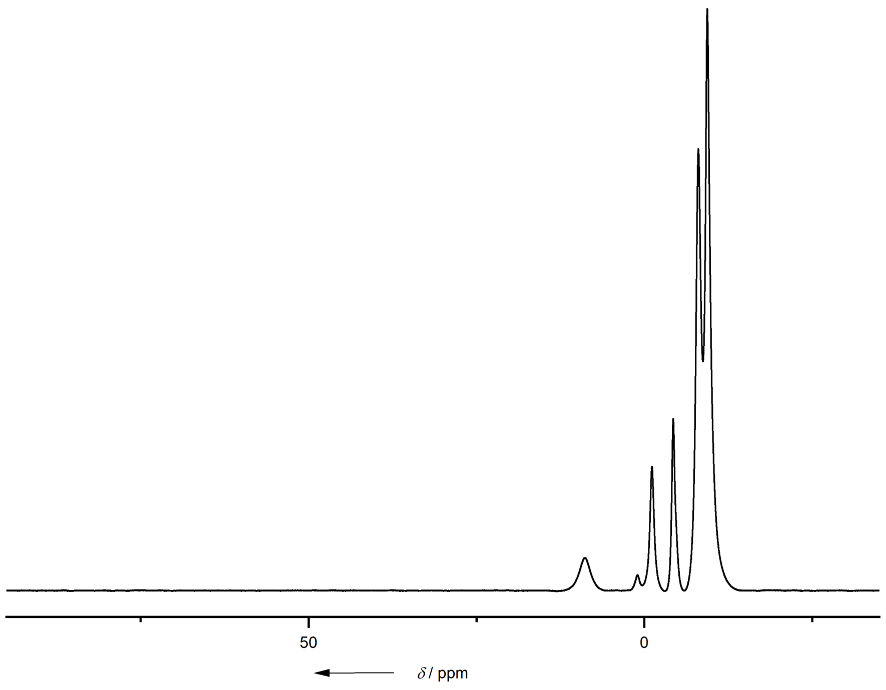


**Figure S10.** $^{11}B\{^{1}H\}$ NMR spectrum of **2-acetone** in $C_6D_6$.

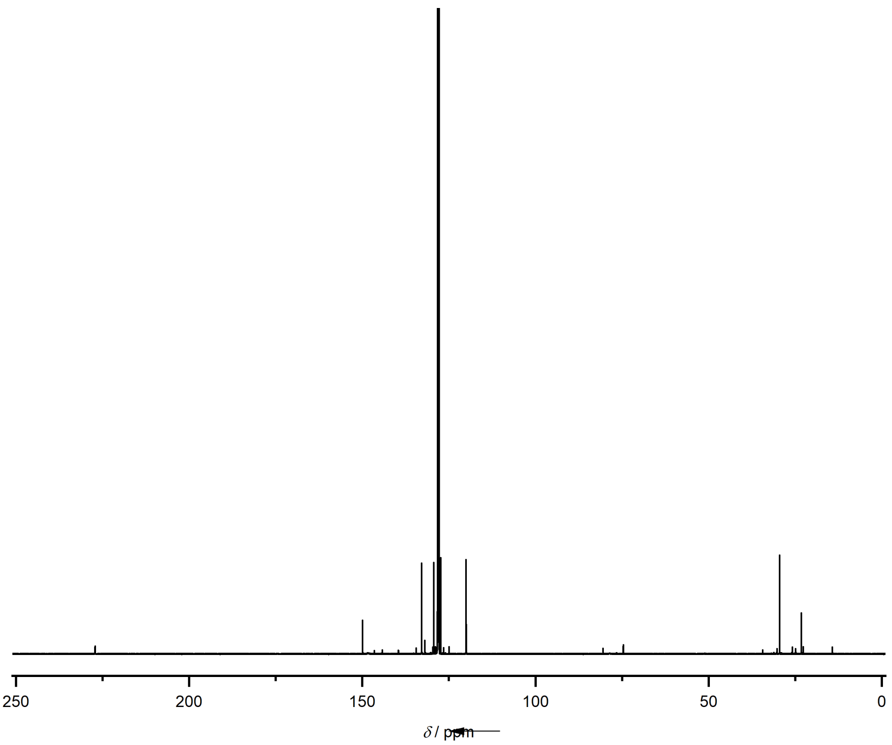


**Figure S11.** $^{13}C$ NMR spectrum of **2-acetone** in $C_6D_6$.

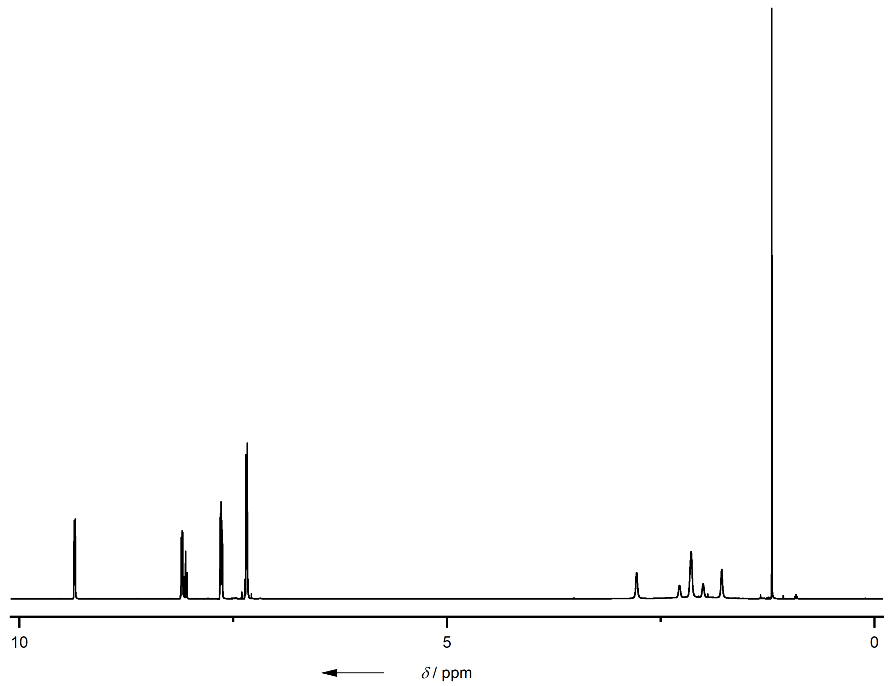


**Figure S12.** $^{1}H$ NMR spectrum of **2-pyridine** in $C_6D_6$.

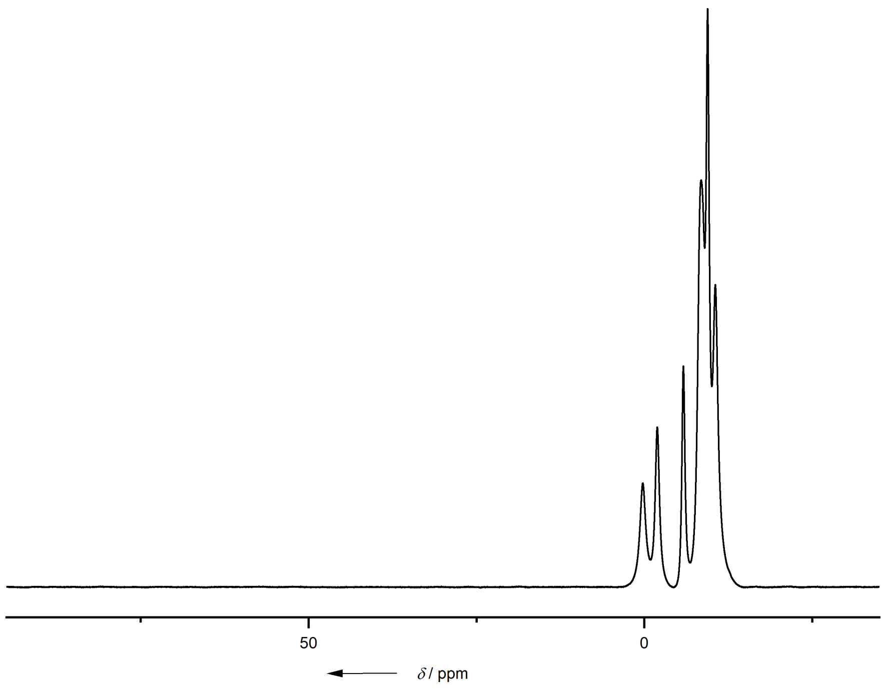


**Figure S13.** $^{11}B\{^1H\}$ NMR spectrum of **2-pyridine** in $C_6D_6$.

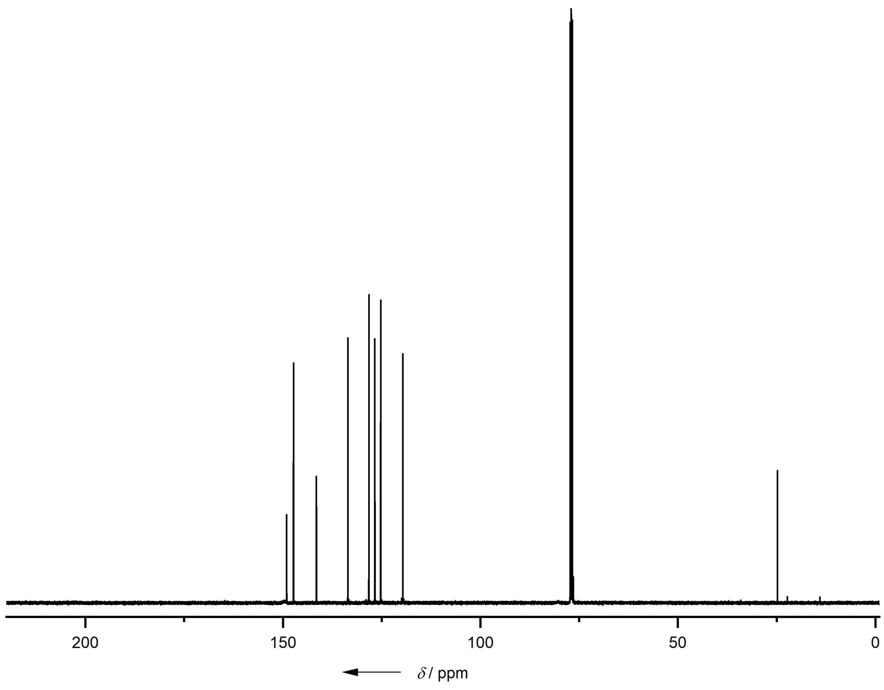


**Figure S14.** $^{13}C$ NMR spectrum of **2-pyridine** in $C_6D_6$.

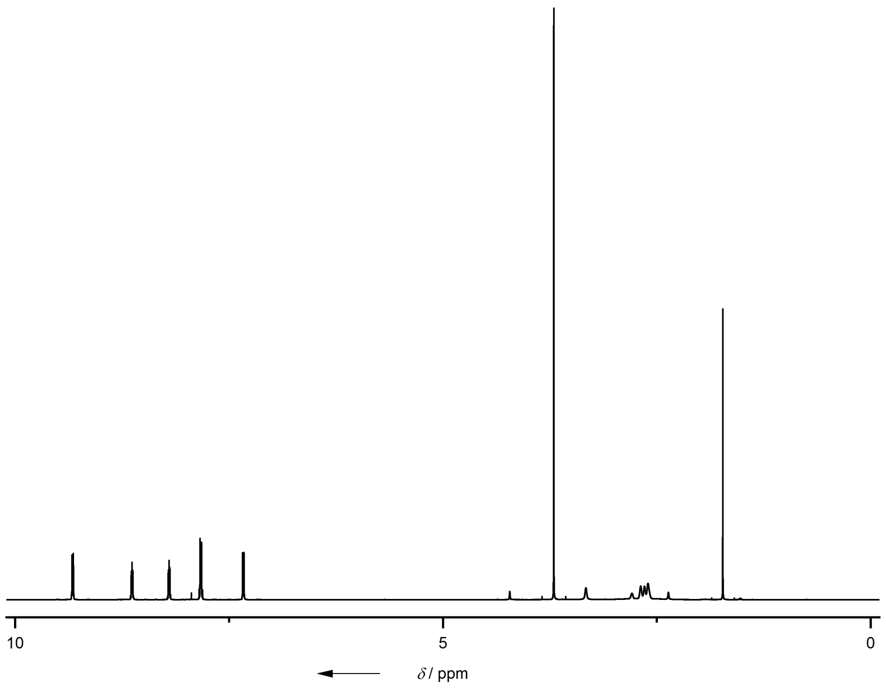


**Figure S15.** $^{1}H$ NMR spectrum of **2-DMAP** in $C_6D_6$.

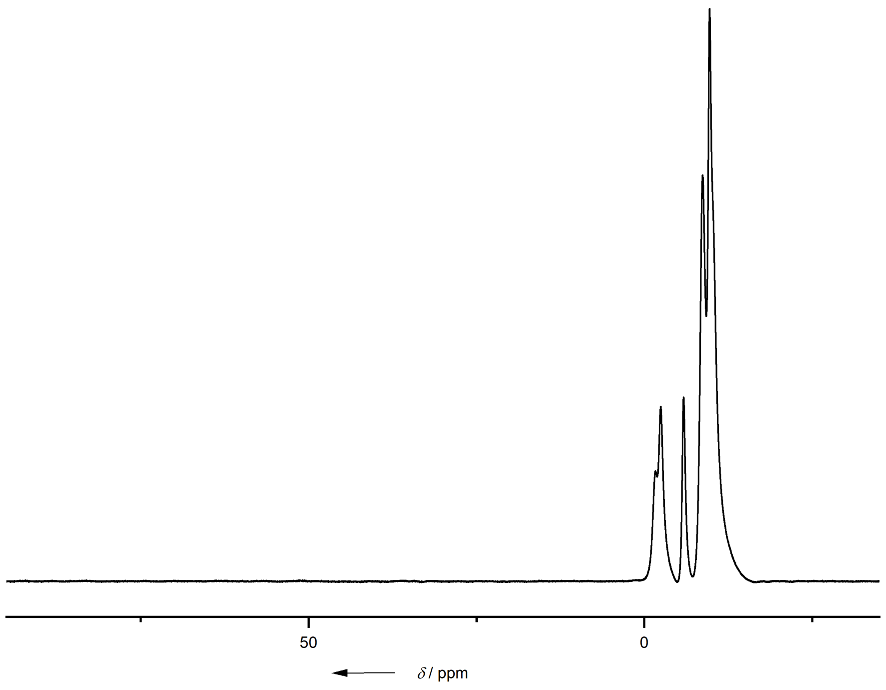


**Figure S16.** $^{11}B\{^{1}H\}$ NMR spectrum of **2-DMAP** in $C_6D_6$.

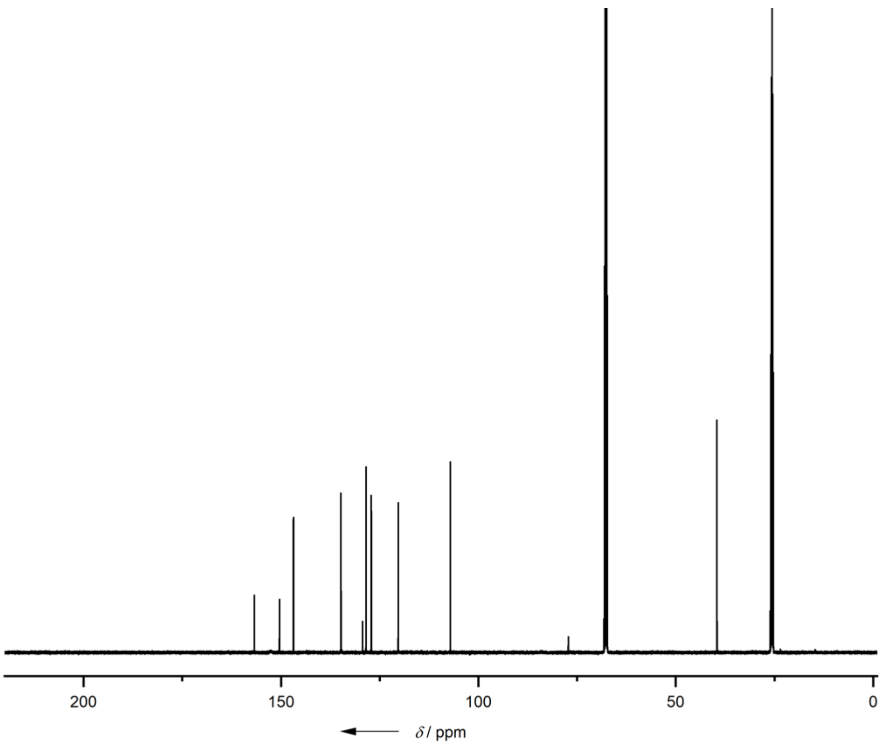


**Figure S17.** $^{13}C$ NMR spectrum of **2-DMAP** in $C_6D_6$.

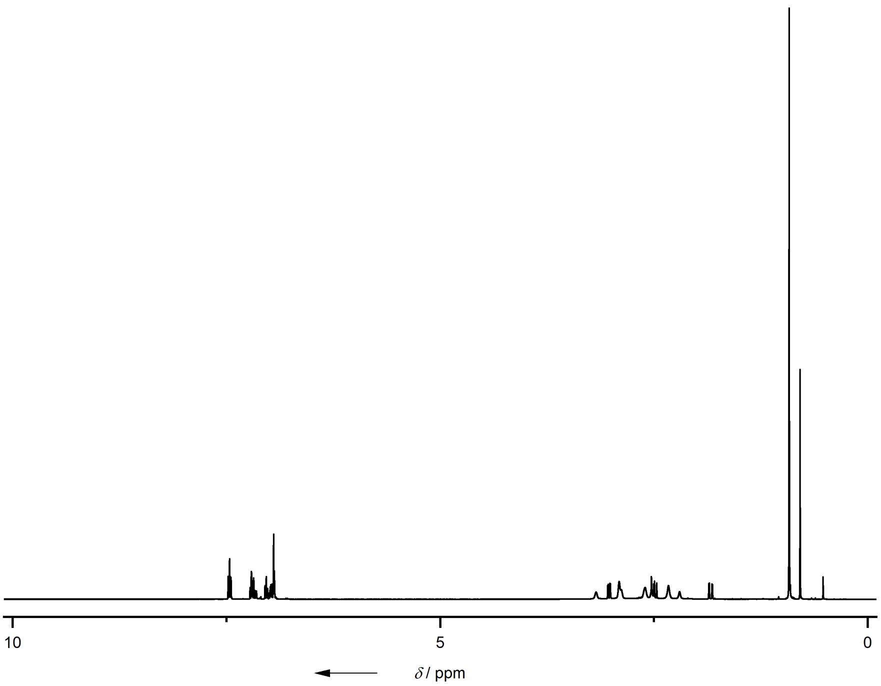


**Figure S18.** $^{1}H$ NMR spectrum of **3** in $C_6D_6$.

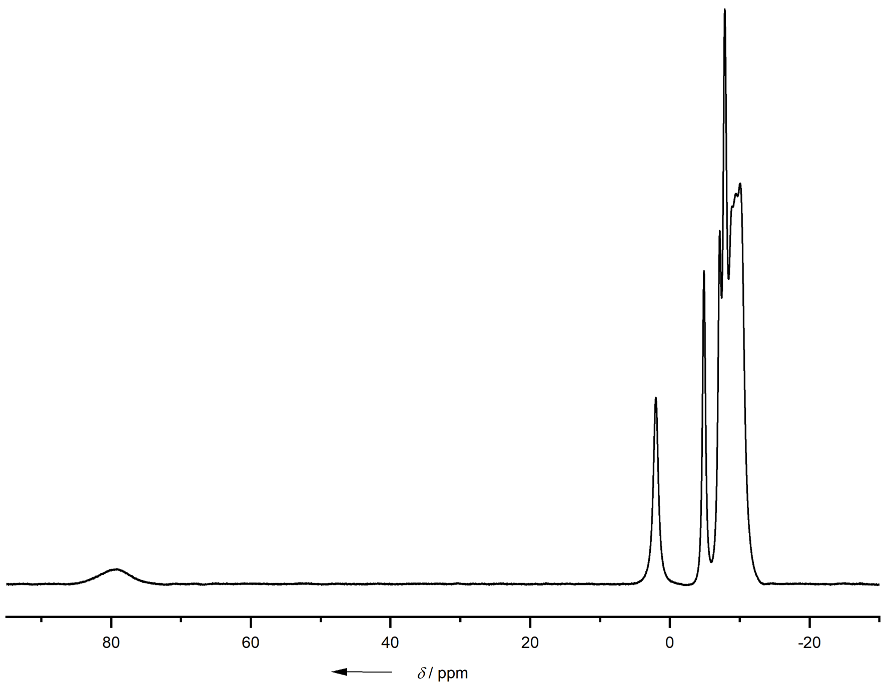


**Figure S19.** $^{11}B\{^{1}H\}$ NMR spectrum of **3** in $C_6D_6$.

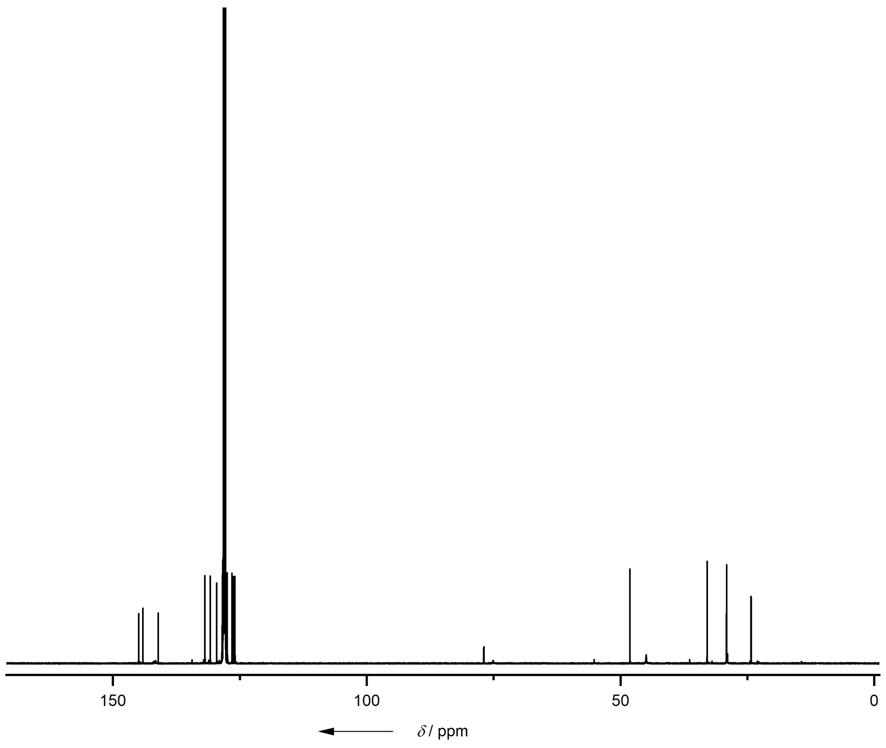


**Figure S20.** $^{13}C$ NMR spectrum of **3** in $C_6D_6$.

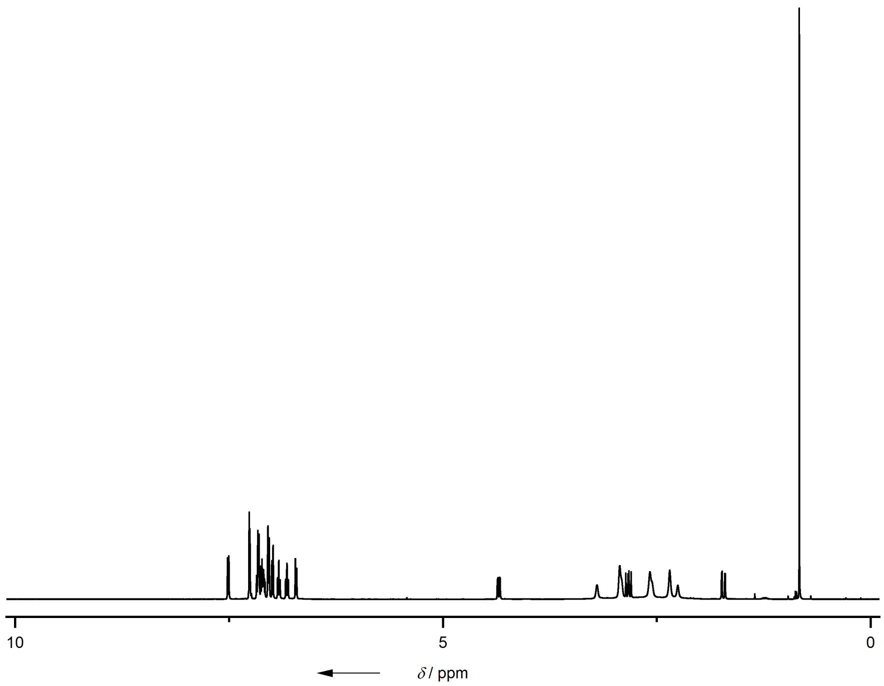


**Figure S21.** $^{1}H$ NMR spectrum of **4** in $C_6D_6$.

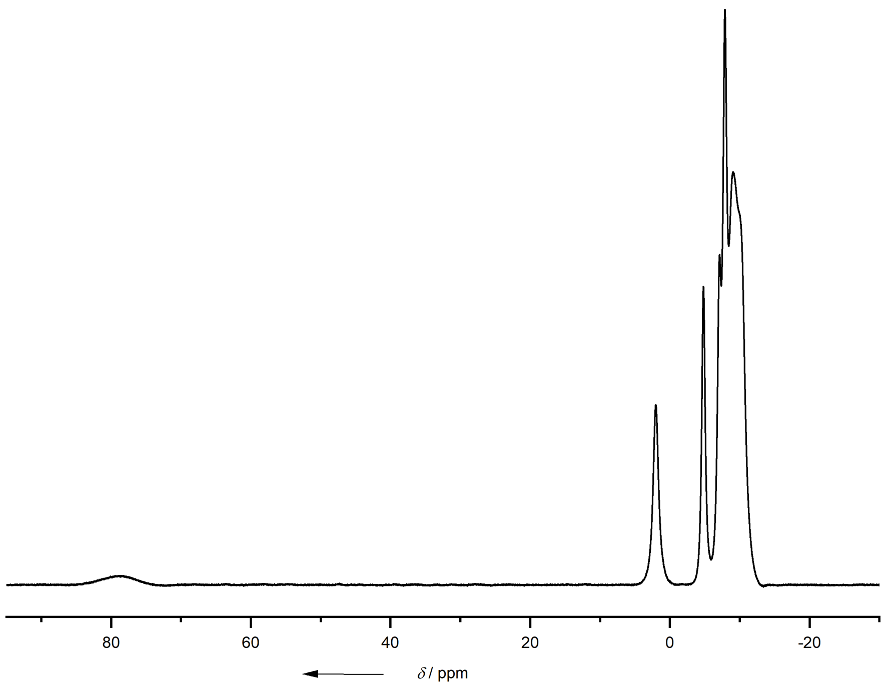


**Figure S22.** $^{11}B\{^{1}H\}$ NMR spectrum of **4** in $C_6D_6$.

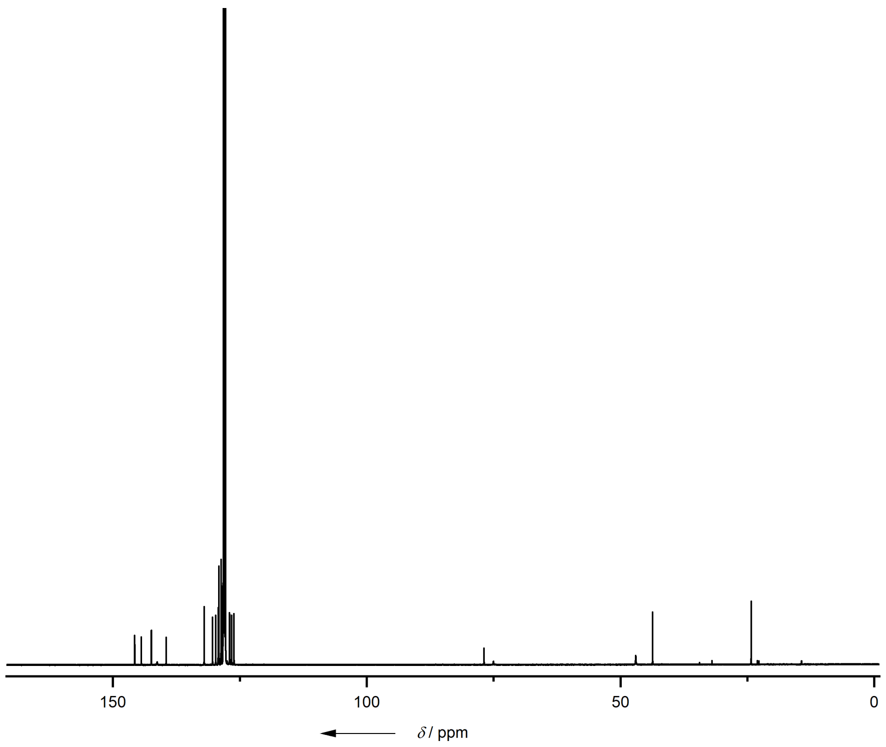


**Figure S23.** $^{13}C$ NMR spectrum of **4** in $C_6D_6$.

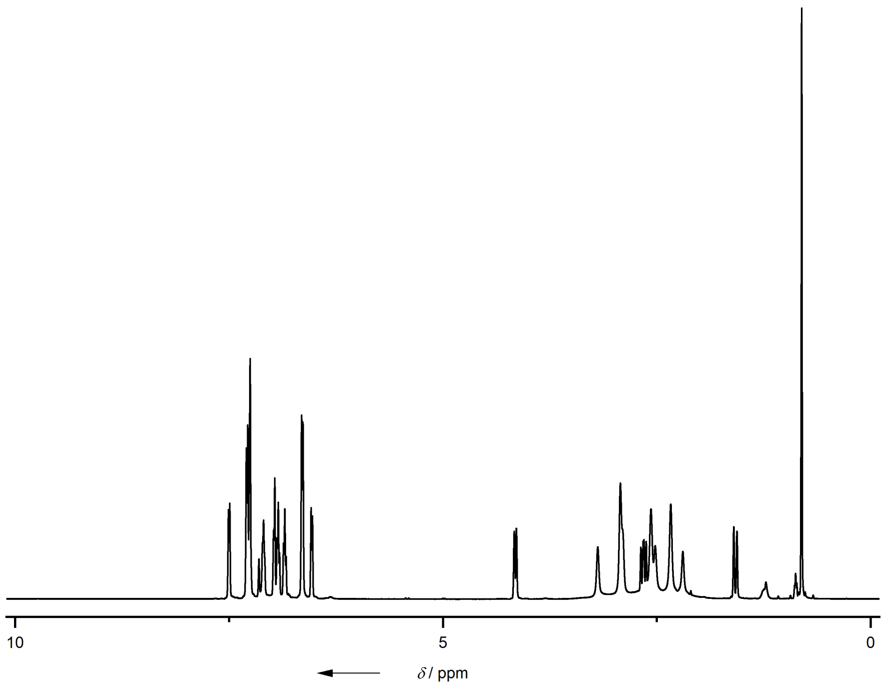


**Figure S24.** $^{1}H$ NMR spectrum of **5** in $C_6D_6$.

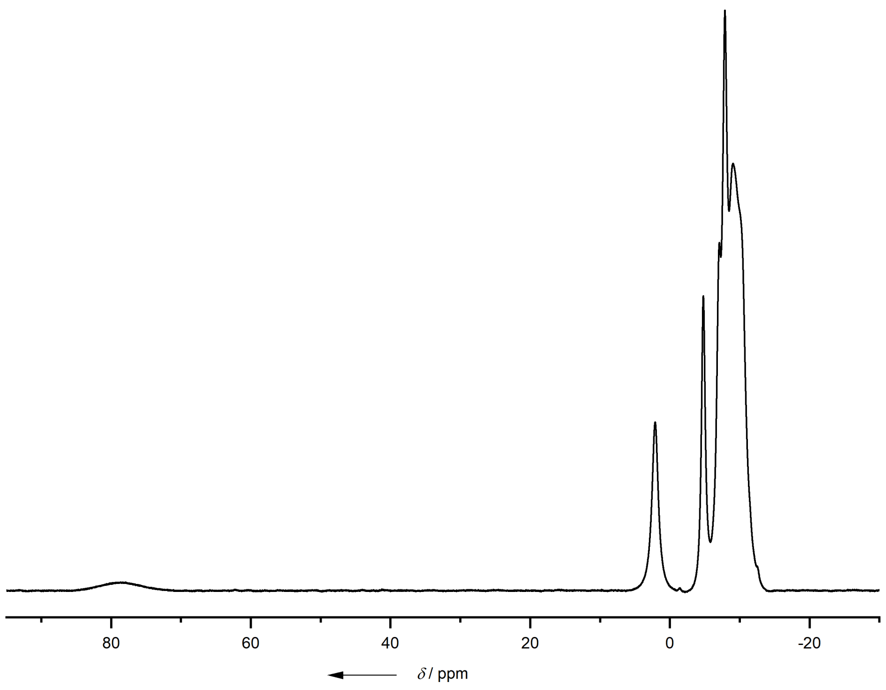


**Figure S25.** $^{11}B\{^{1}H\}$ NMR spectrum of **5** in $C_6D_6$.

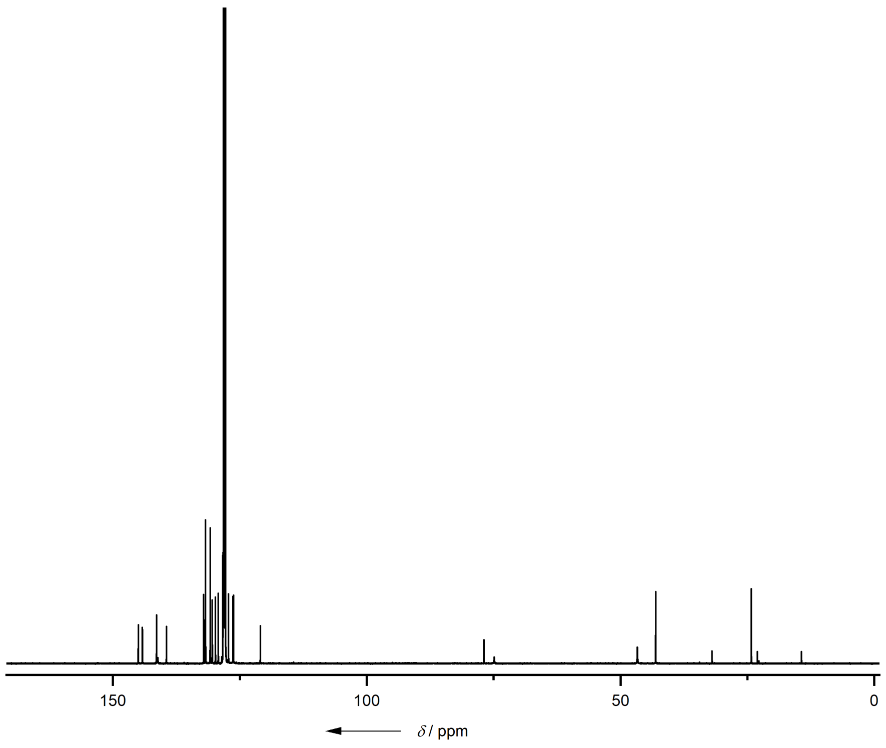


**Figure S26.** $^{13}C$ NMR spectrum of **5** in $C_6D_6$.

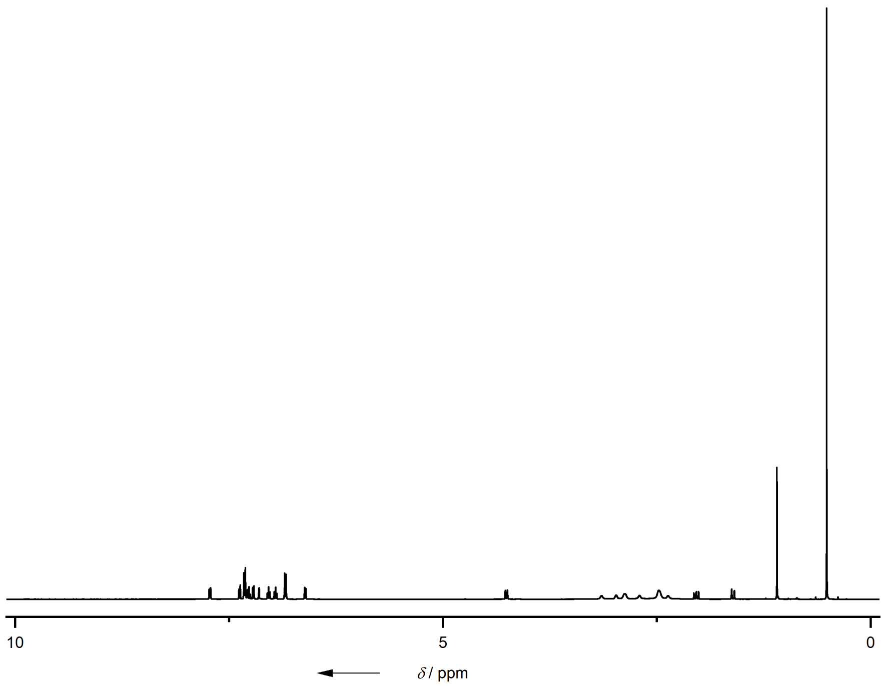


Figure S27. $^{1}H$ NMR spectrum of **5-*t*BuCN** in $C_6D_6$.

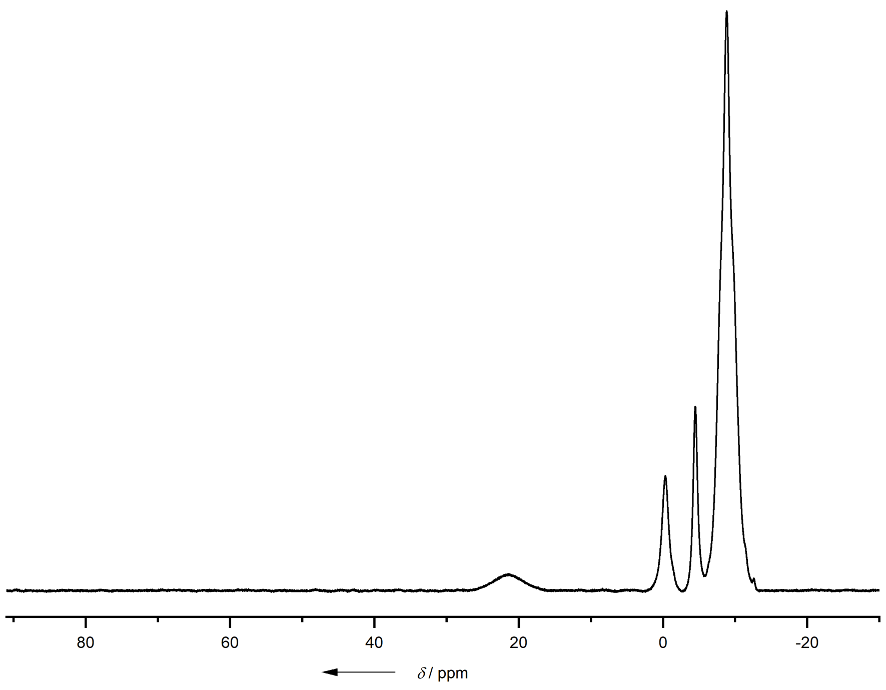


Figure S28. $^{11}B\{^{1}H\}$ NMR spectrum of **5-*t*BuCN** in $C_6D_6$.

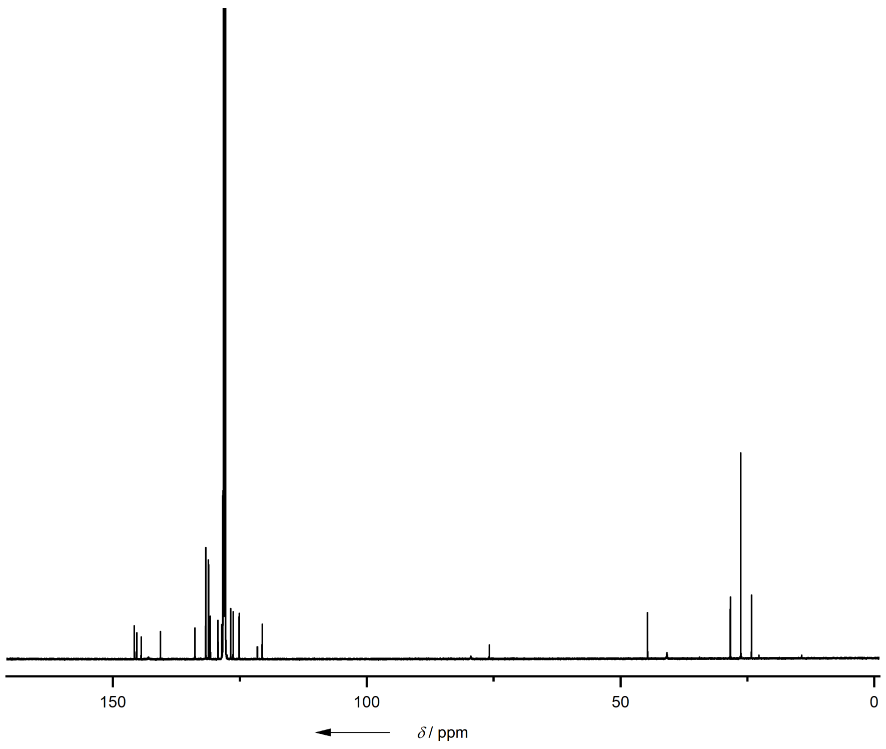


**Figure S29.** $^{13}C$ NMR spectrum of **5-*t*BuCN** in $C_6D_6$.

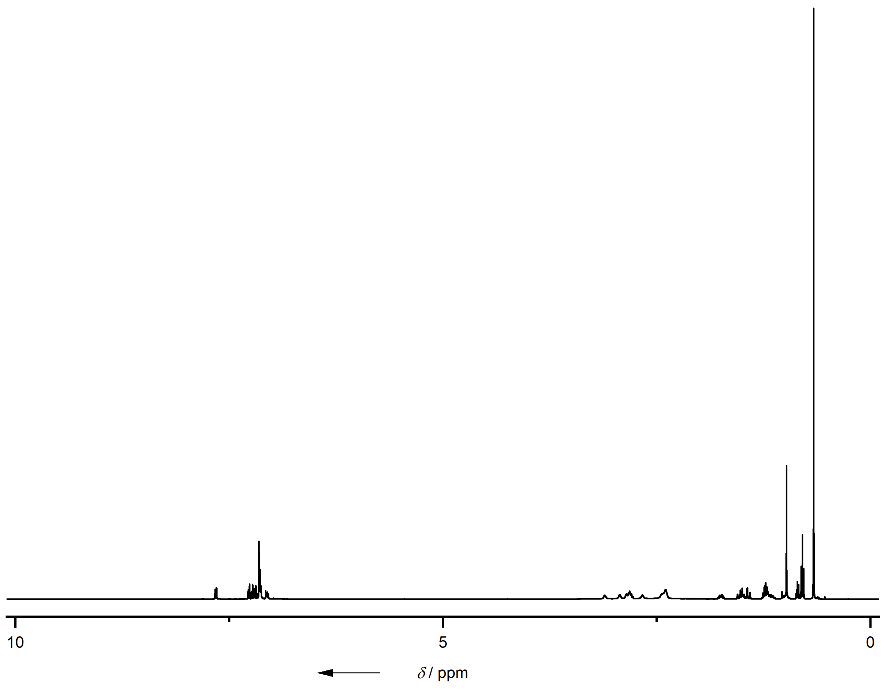


**Figure S30.** $^{1}H$ NMR spectrum of **6-*t*BuCN** in $C_6D_6$.

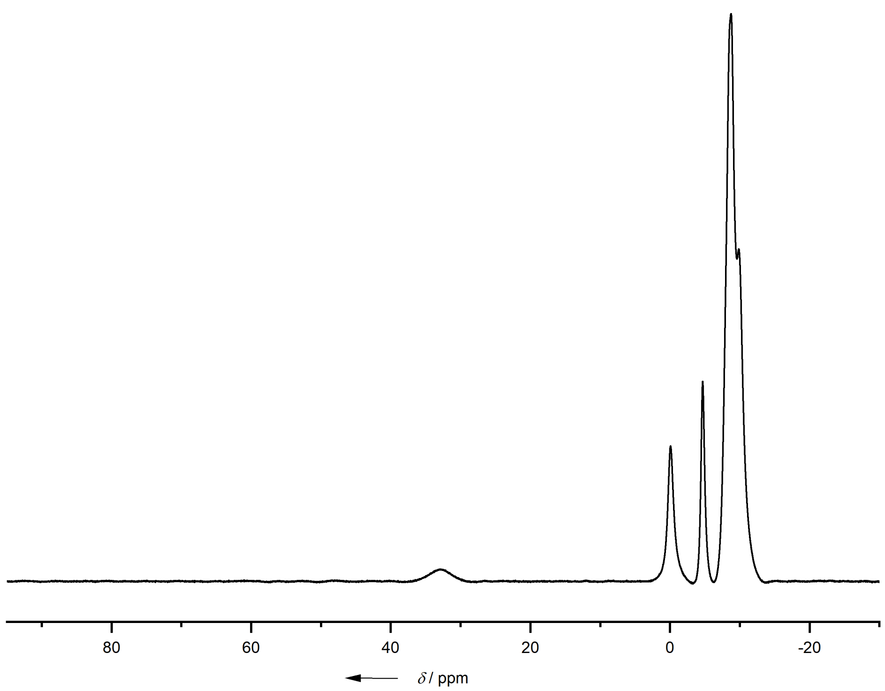


**Figure S31.** $^{11}B\{^{1}H\}$ NMR spectrum of **6-*t*BuCN** in $C_6D_6$.

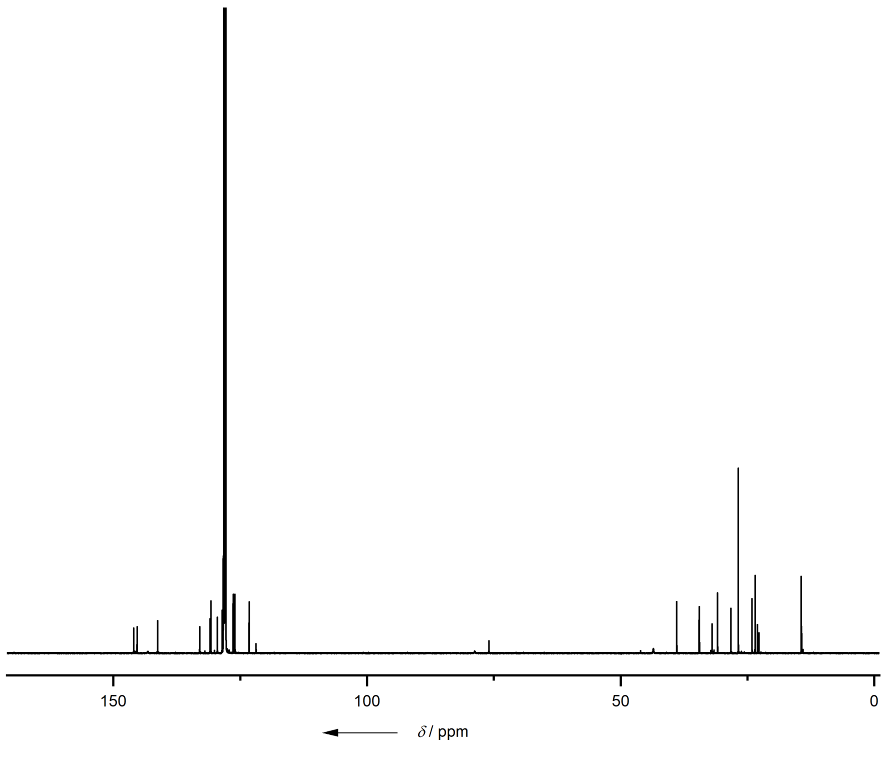


**Figure S32.** $^{13}C$ NMR spectrum of **6-*t*BuCN** in $C_6D_6$.

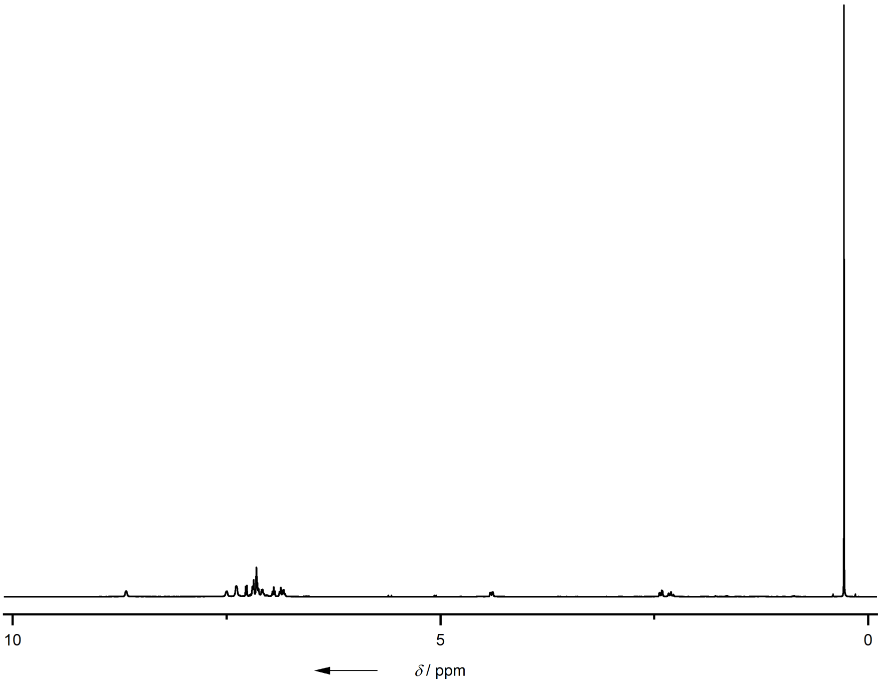


**Figure S33.** $^{1}H$ NMR spectrum of **7-*t*BuCN** in $C_6D_6$.

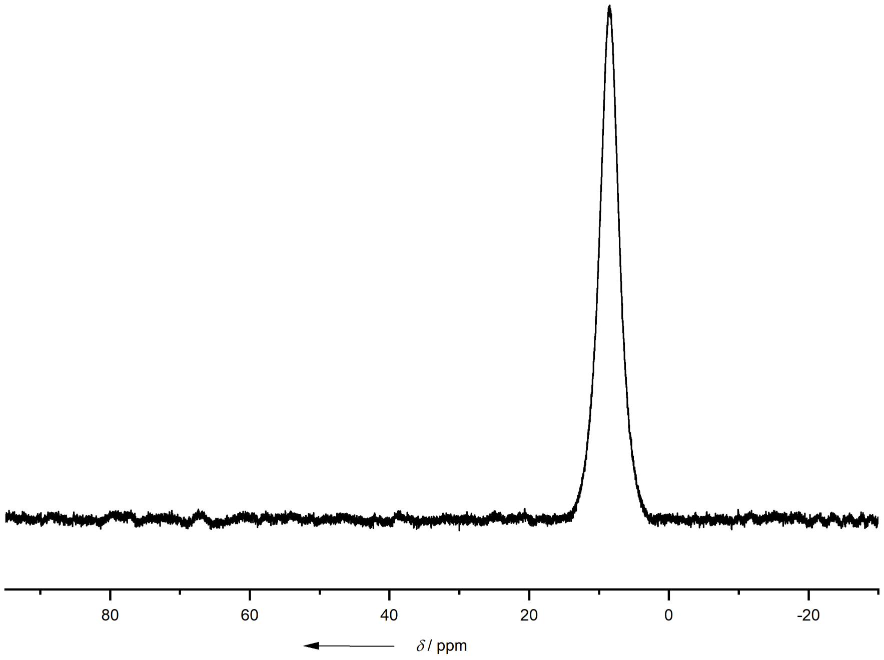


**Figure S34.** $^{11}B\{^{1}H\}$ NMR spectrum of **7-*t*BuCN** in $C_6D_6$.

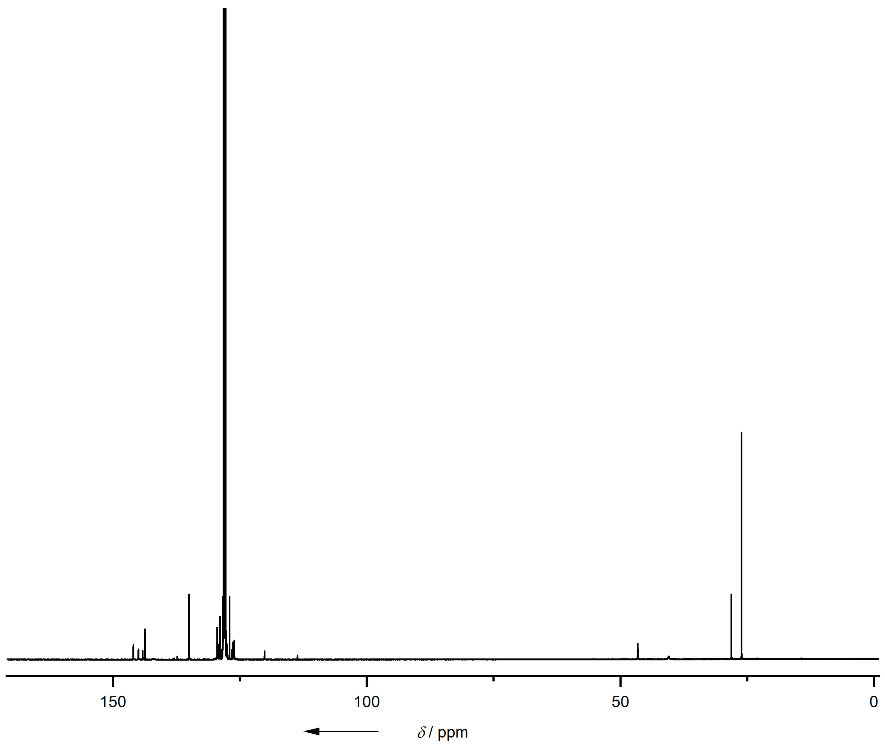


**Figure S35.** $^{13}$C NMR spectrum of **7-*t*BuCN** in $C_6D_6$.

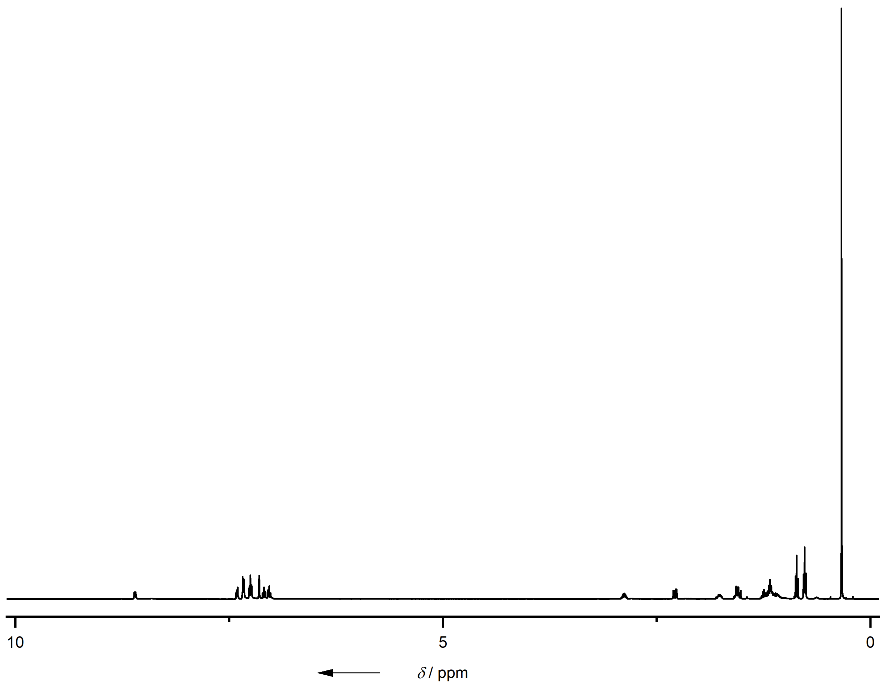


**Figure S36.** $^{1}$H NMR spectrum of **8-*t*BuCN** in $C_6D_6$.

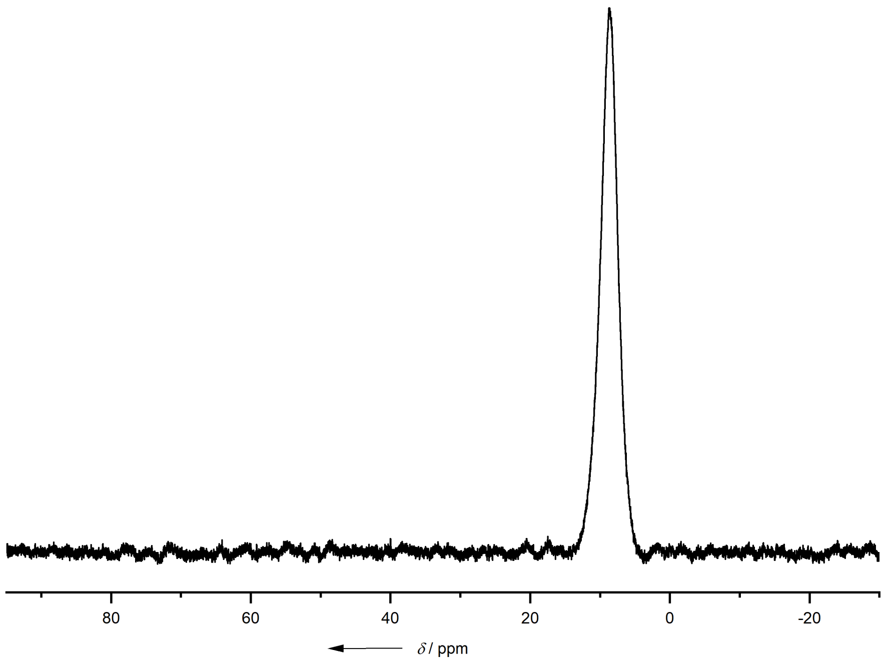


**Figure S37.** $^{11}B\{^{1}H\}$ NMR spectrum of **8-*t*BuCN** in $C_6D_6$.

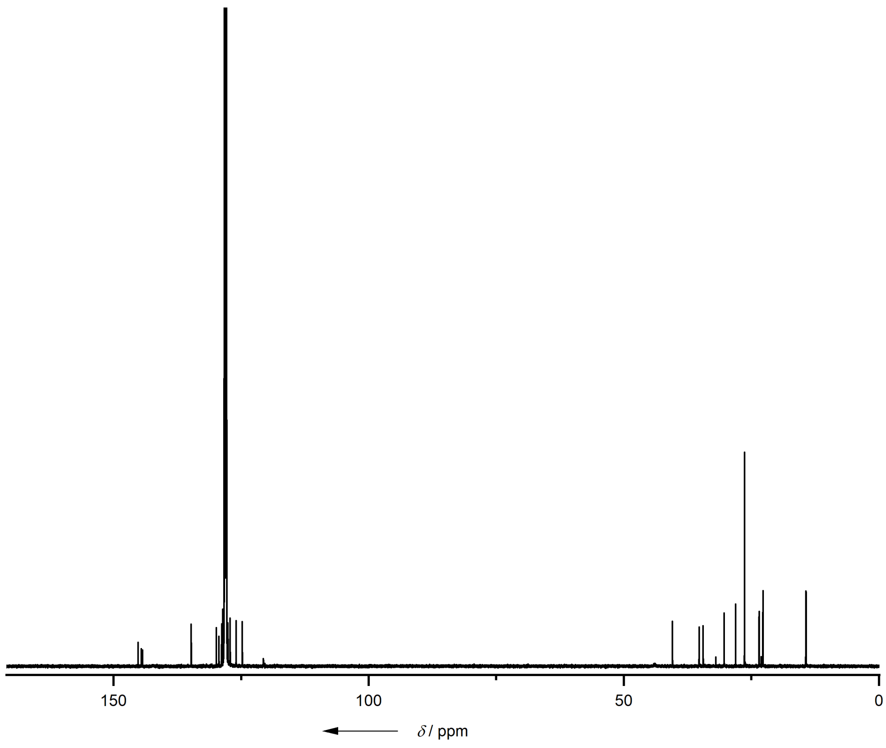


**Figure S38.** $^{13}C$ NMR spectrum of **8-*t*BuCN** in $C_6D_6$.

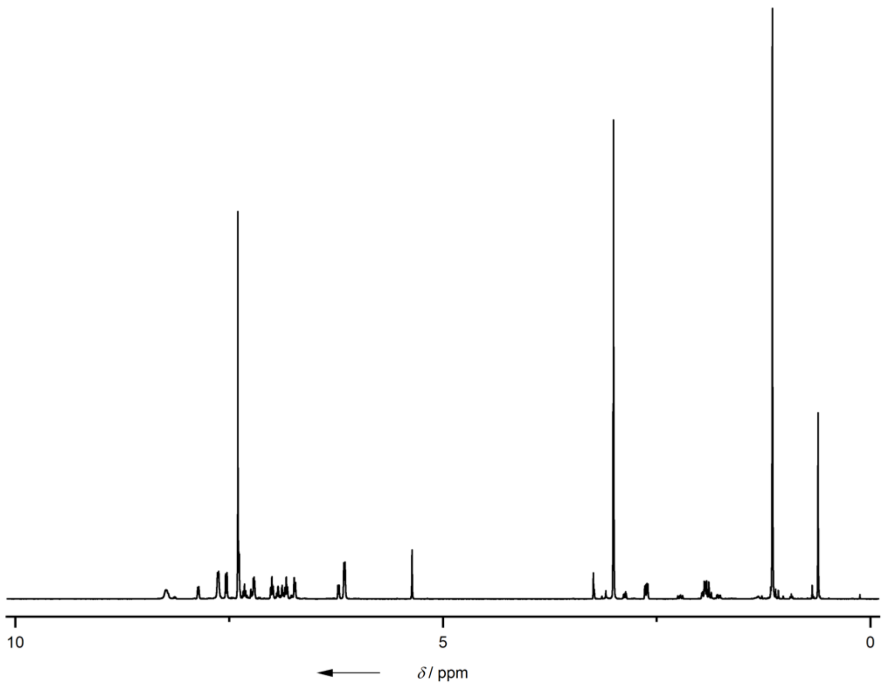


Figure S39. $^{1}$H NMR spectrum of **9-DMAP** in $CD_2Cl_2$.

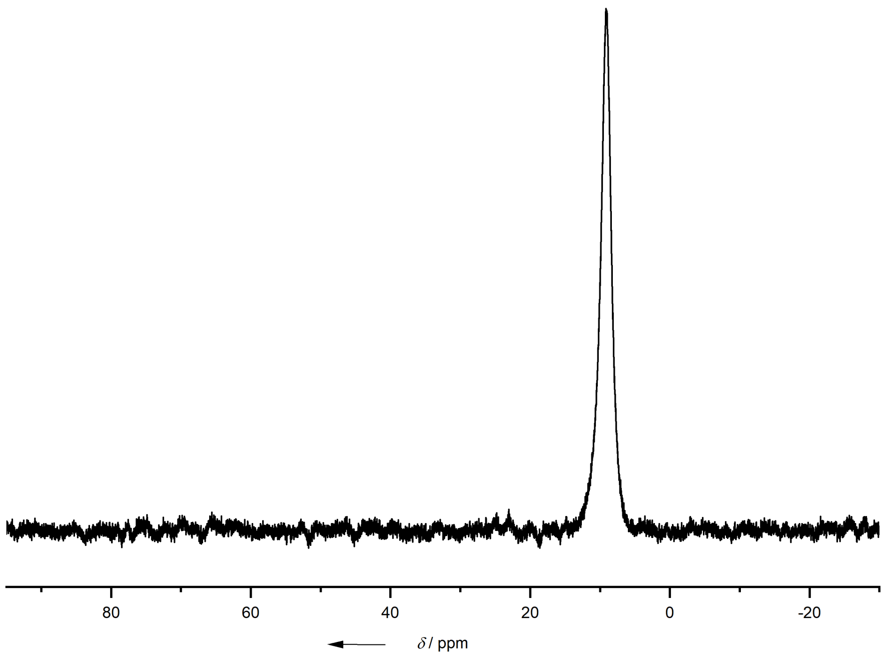


Figure S40. $^{11}$B{$^{1}$H} NMR spectrum of **9-DMAP** in $CD_2Cl_2$.

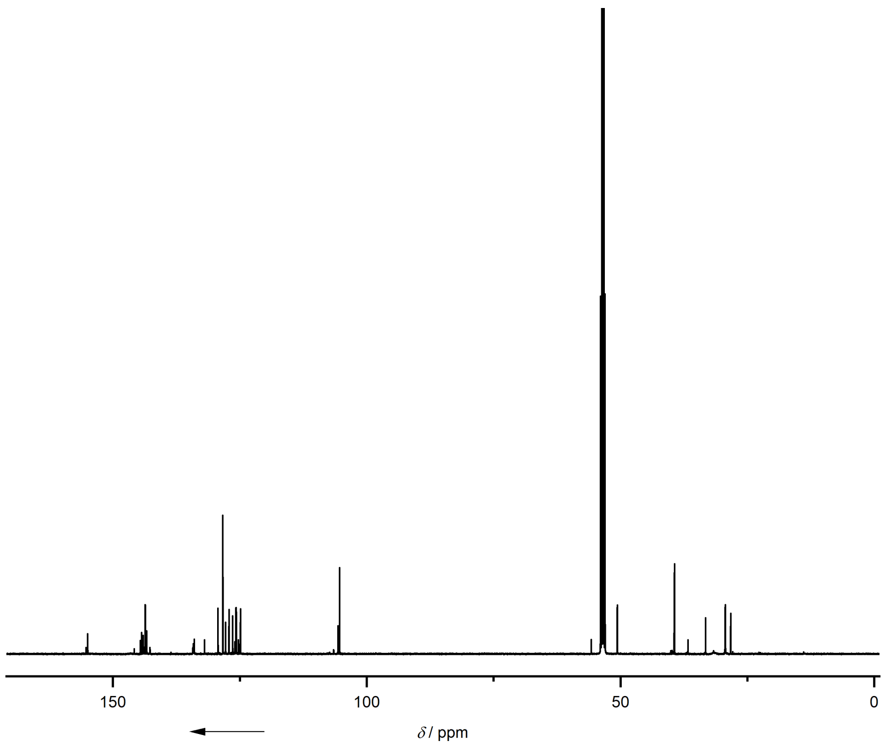


**Figure S41** $^{13}C$ NMR spectrum of **9-DMAP** in $CD_2Cl_2$.

# 4. IR spectra

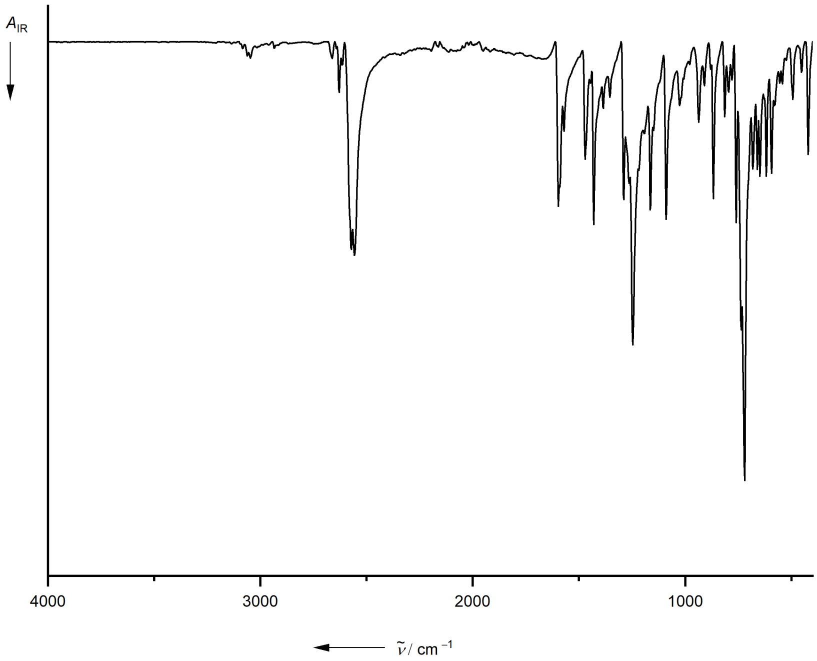


**Figure S42.** IR spectrum of **2**.

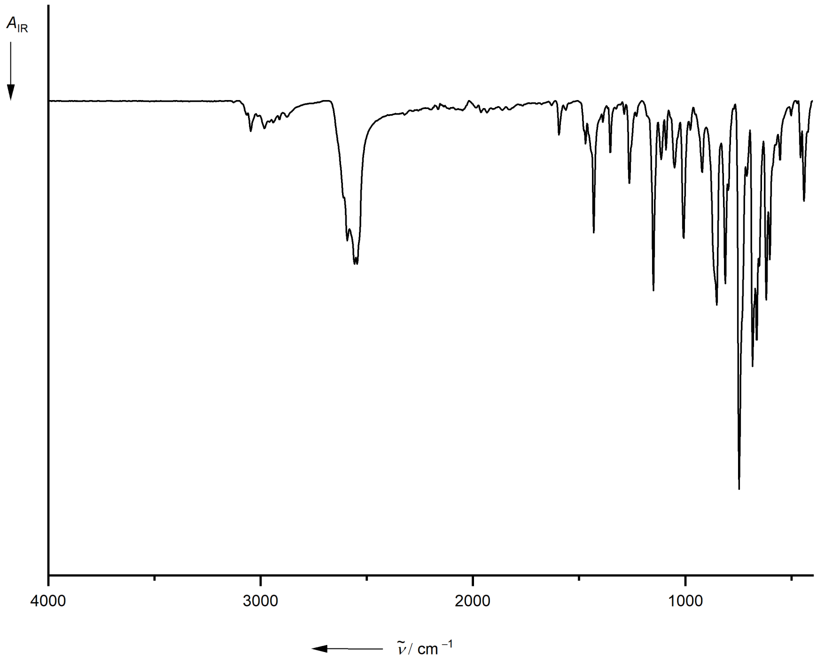


**Figure S43.** IR spectrum of **2-thf**.

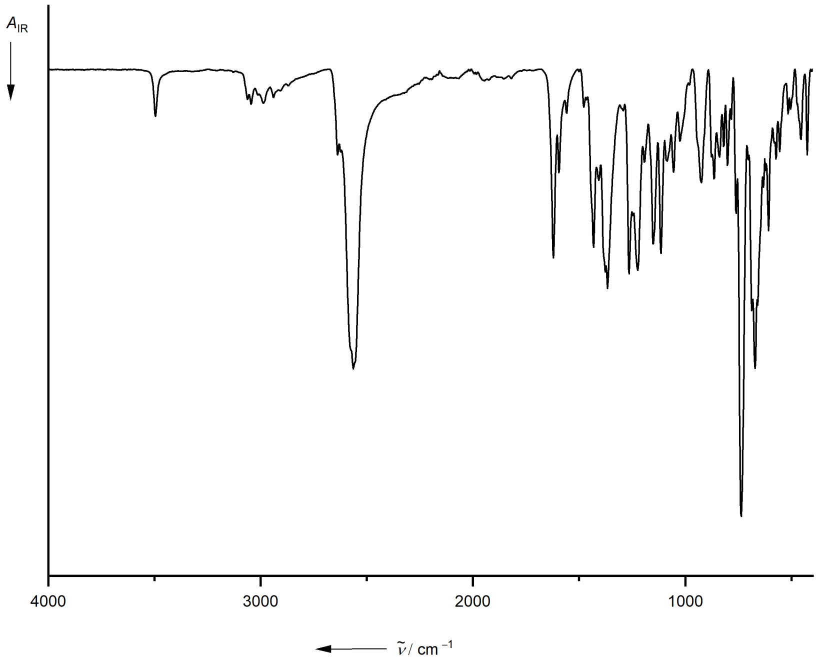


**Figure S44.** IR spectrum of **2-acetone**.

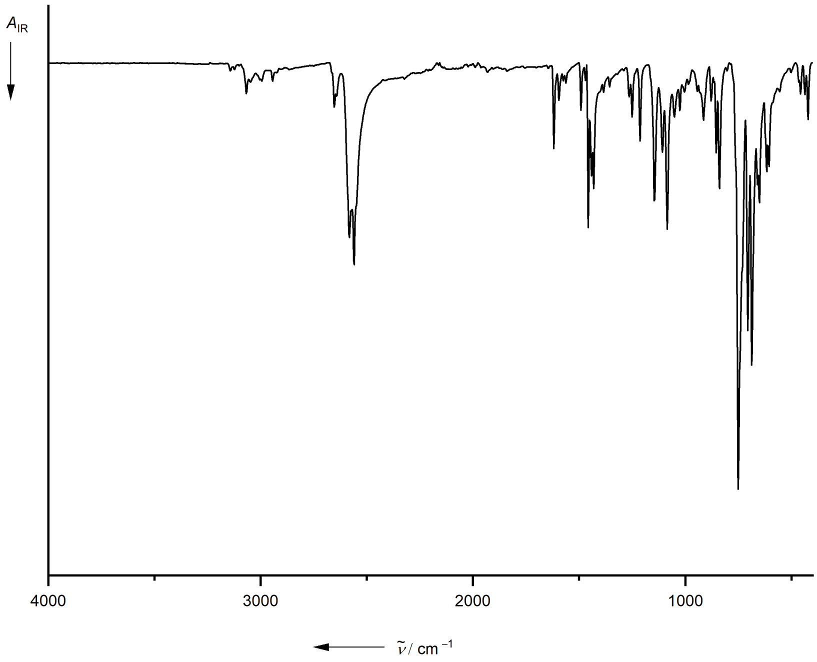


**Figure S45.** IR spectrum of **2-pyridine**.

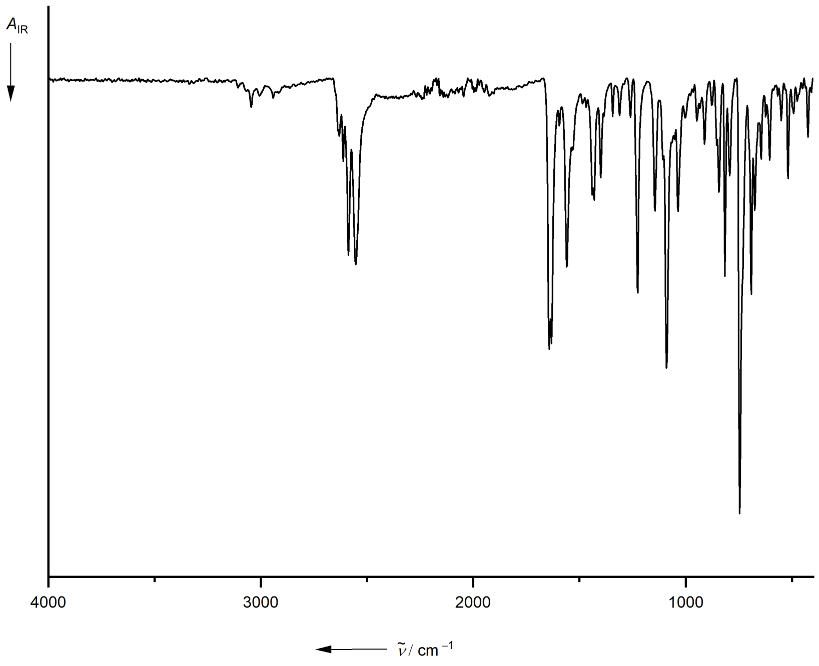


**Figure S46.** IR spectrum of **2-DMAP**.

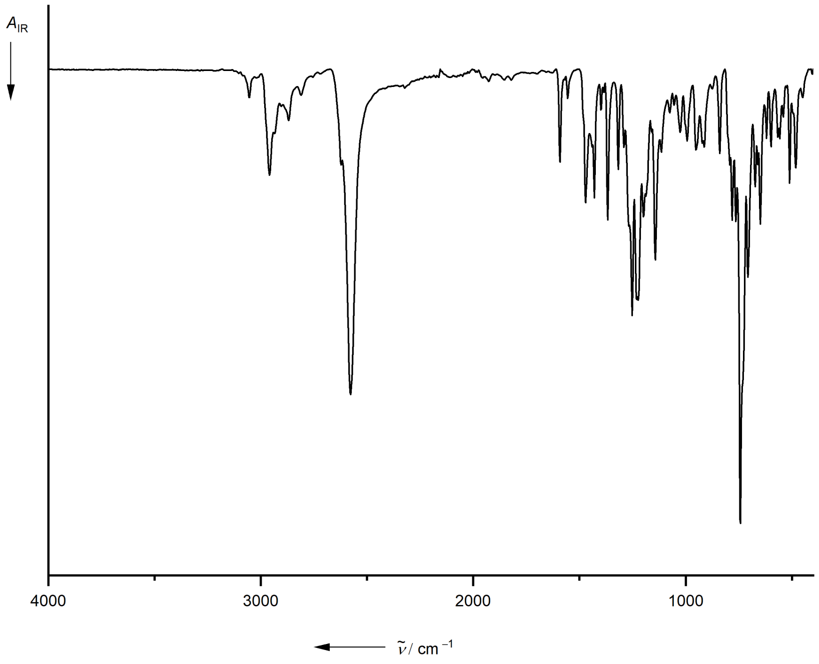


**Figure S47.** IR spectrum of **3**.

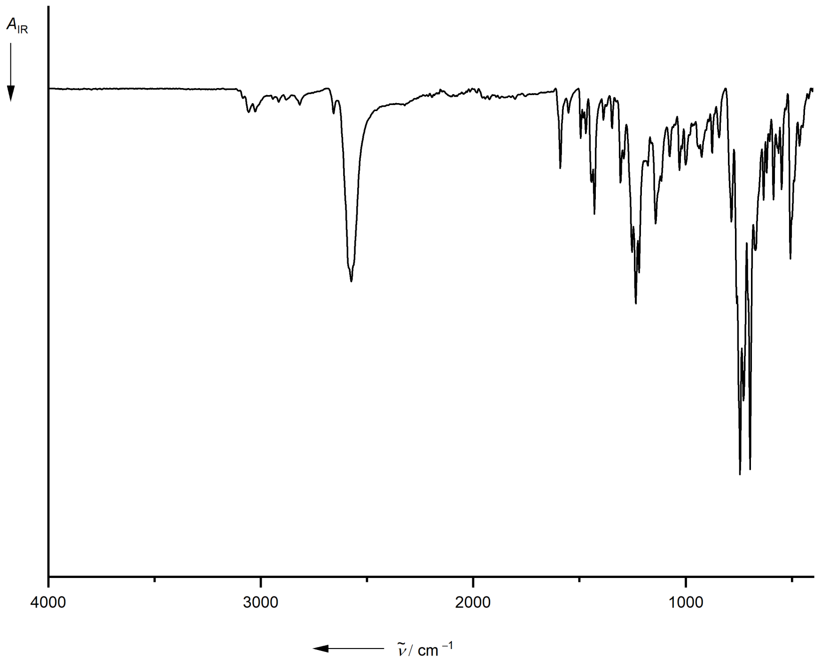


**Figure S48.** IR spectrum of **4**.

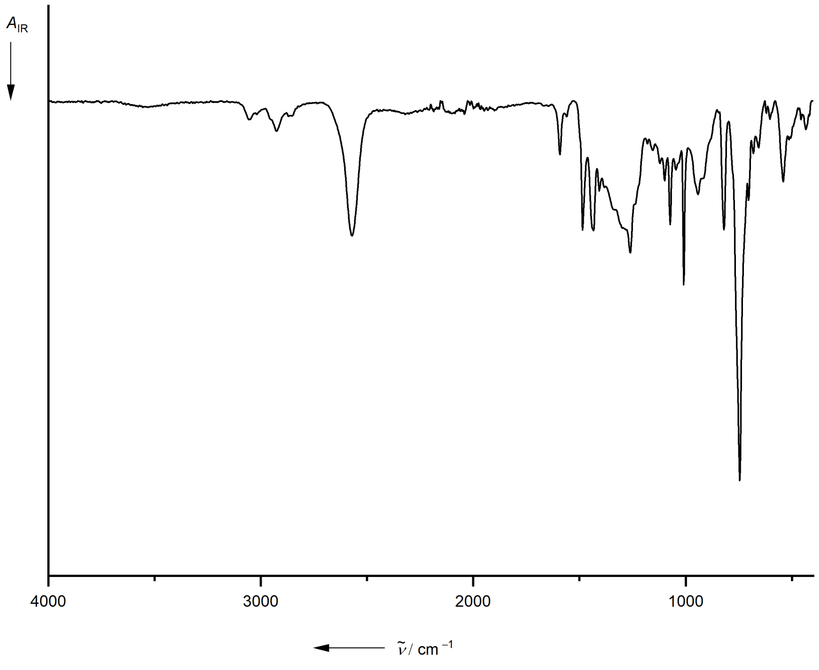


**Figure S49.** IR spectrum of **5**.

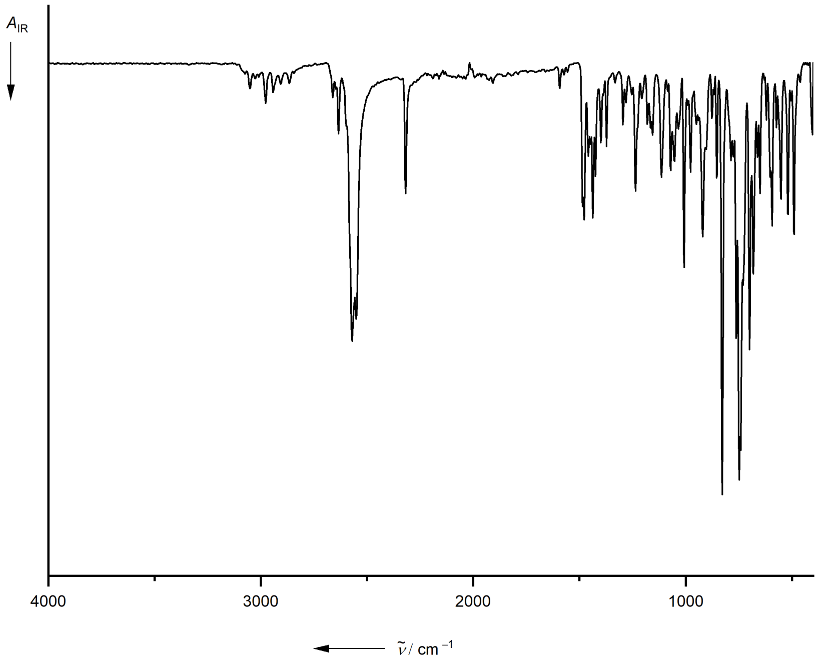


**Figure S50.** IR spectrum of **5-*t*BuCN**.

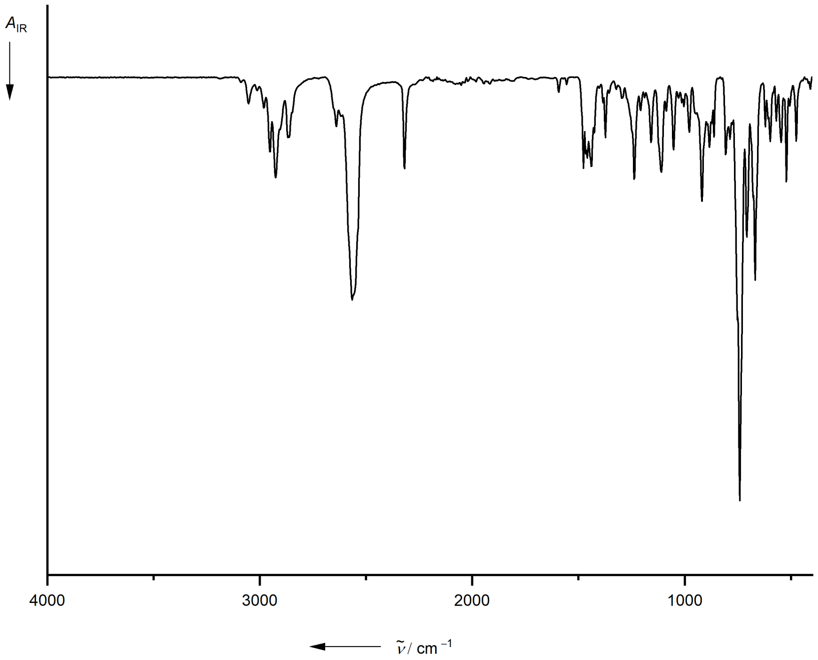


**Figure S51.** IR spectrum of **6-*t*BuCN**.

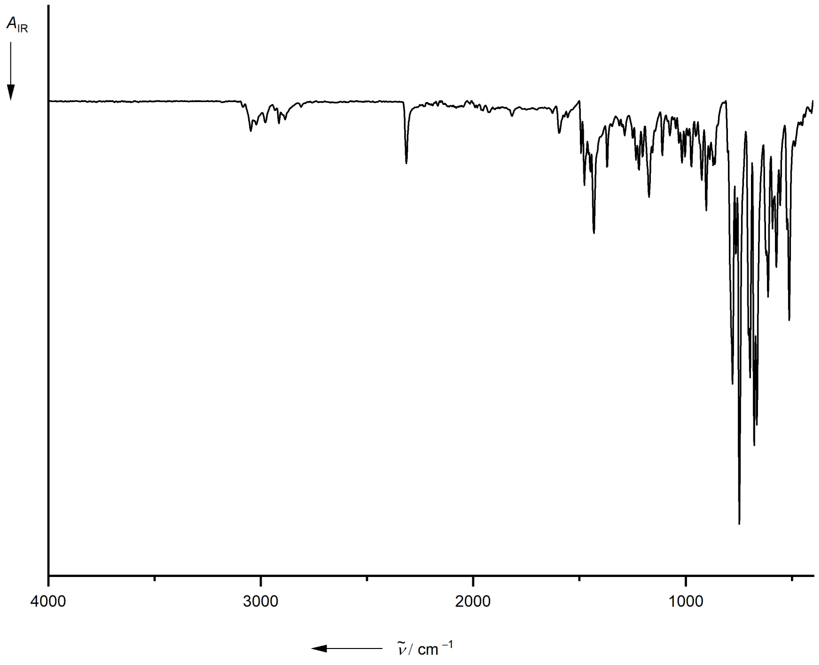


**Figure S52.** IR spectrum of **7-tBuCN**.

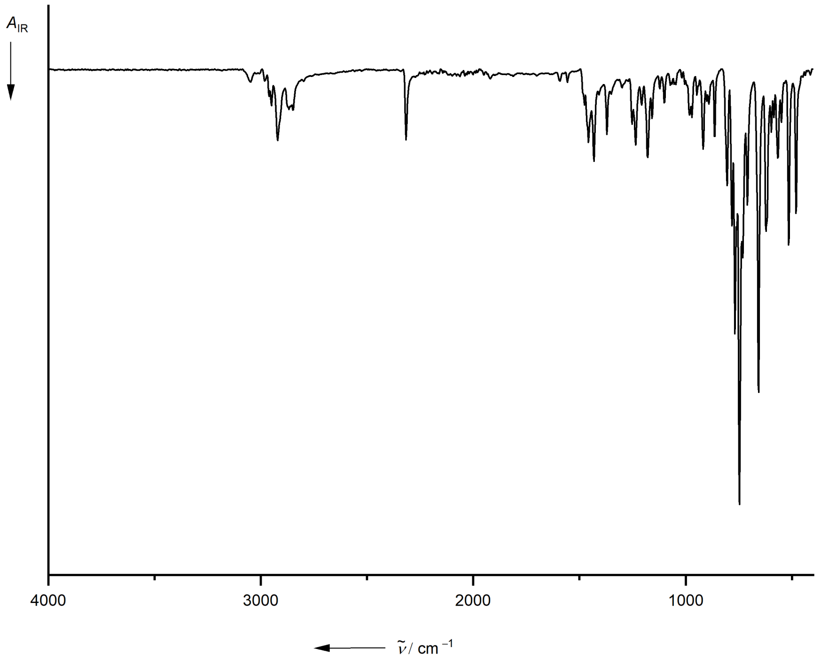


**Figure S53.** IR spectrum of **8-*t*BuCN**.

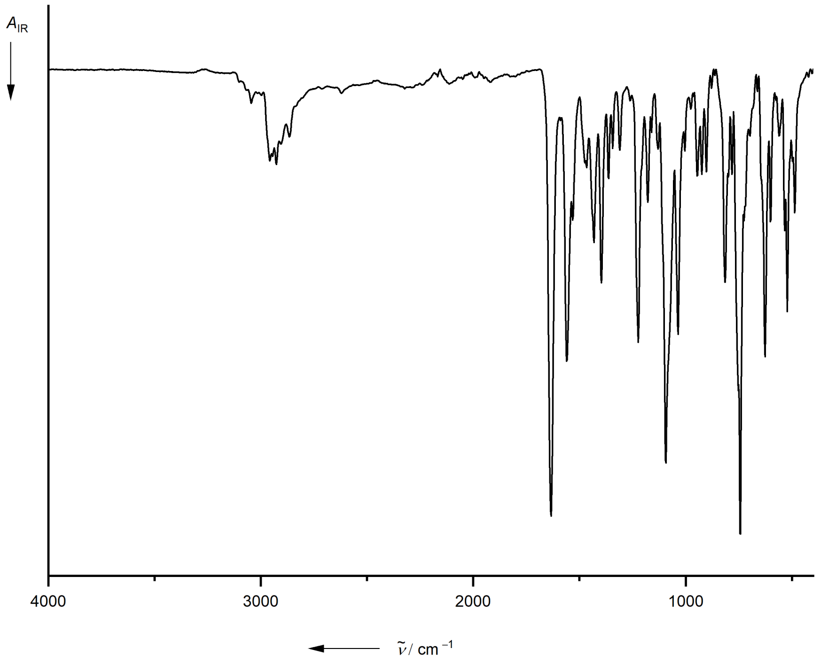


**Figure S54.** IR spectrum of **9-DMAP**.

## 5. Cyclic voltammetry studies

Cyclic voltammetry experiments were performed using a Gamry Instruments Reference 600 potentiostat. A standard three-electrode cell configuration was employed using a platinum disk working electrode, a platinum wire counter electrode, and a silver wire, separated by a Vycor tip, serving as the reference electrode. Formal redox potentials are referenced to the ferrocene/ferrocenium ($[Cp_2Fe]^{+/0}$) redox couple by using ferrocene as an internal standard. Tetra-n-butylammonium hexafluorophosphate ($[nBu_4N][PF_6]$) was employed as the supporting electrolyte. Compensation for resistive losses (*iR* drop) was employed for all measurements.

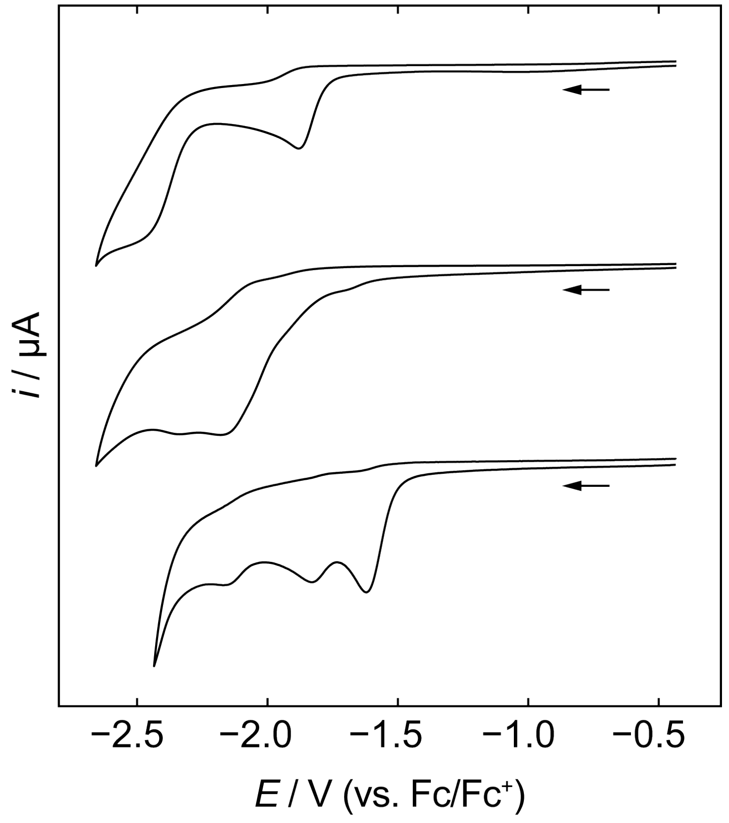


**Figure S55.** Cyclic voltammograms of 9-phenyl-9-borafluorene (**1Ph**, top), 9-bromo-9-borafluorene (**1Br**, center) and **2** (bottom) in $CH_2Cl_2$/0.1 M $[nBu_4N][PF_6]$ measured at 250 mV $s^{-1}$. Formal potentials: **1Ph**: $E_{pc1}$ = −1.87 V, $E_{pc2}$ = −2.44 V; **1Br**: $E_{pc1}$ = −2.21 V (broad, onset at −1.84 V), $E_{pc2}$ = −2.33 V, **2**: $E_{pc1}$ = −1.63 V, $E_{pc2}$ = −1.83 V, $E_{pc3}$ = −2.17 V (relative to the $Fc/Fc^+$ couple).

The compound 2-methyl-1,2-dicarba-*closo*-dodecaborane(12) shows no reduction peak under the same conditions, indicating a reduction potential that is more negative than −2.5 V (vs. Fc/Fc+).

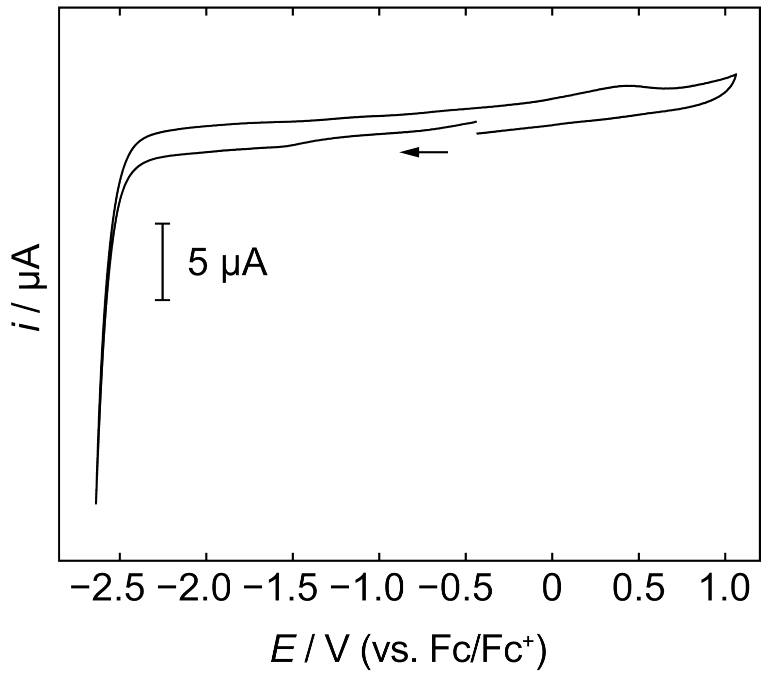


**Figure S56.** Cyclic voltammogram of 2-methyl-1,2-dicarba-*closo*-dodecaborane(12) in $CH_2Cl_2$/0.1 M [*n*$Bu_4N$][$PF_6$] measured at 250 mV $s^{-1}$.

# 6. X-ray crystallographic data

The crystal data of **2**, **2-thf**, **2-acetone**, **2-pyridine**, **2-DMAP**, **3**, **4**, **5** and **5-*t*BuCN** were collected on a XtaLAB Synergy, Dualflex diffractometer with a HyPix area detector and multi-layer mirror monochromated Cu $K_{\alpha}$ radiation. The crystal data of **6-*t*BuCN**, **7-*t*BuCN**, **8-*t*BuCN** and **9-DMAP** were collected on a XtaLAB Synergy, rotating-anode X-ray tube with a HyPix-Arc 150 area detector and multi-layer mirror monochromated Cu $K_{\alpha}$ radiation.

The structures were solved using the intrinsic phasing method,[4] refined with the ShelXL program[5] and expanded using Fourier techniques. All non-hydrogen atoms were refined anisotropically. Hydrogen atoms were included in the structure factor calculations. All hydrogen atoms except H3 to H12 bound to the boron atoms of the carborane moiety were assigned to idealized geometric positions. The coordinates of H3 to H12 were refined freely or constrained to idealized geometric positions depending on the quality of the data.

**Crystal data for 2:** $C_{15}H_{21}B_{11}$, $M_r$ = 320.23, orange block, 0.228 x 0.078 x 0.045 mm$^3$, monoclinic space group $P2_1$, $a$ = 6.73580(10) Å, $b$ = 11.9489(2) Å, $c$ = 11.5149(2) Å, β= 113.731(18) °, $V$ = 902.42(3) Å$^3$, $Z$ = 2, $\rho_{calcd}$ = 1.178 g/cm$^3$, μ = 0.403 mm$^{-1}$, $F$(000) = 332, $T$ = 100(2) K, $R_1$ = 0.0466, $wR^2$ = 0.1221, 3832 independent reflections [2θ ≤155.506°] and 246 parameters. CCDC 2172383.

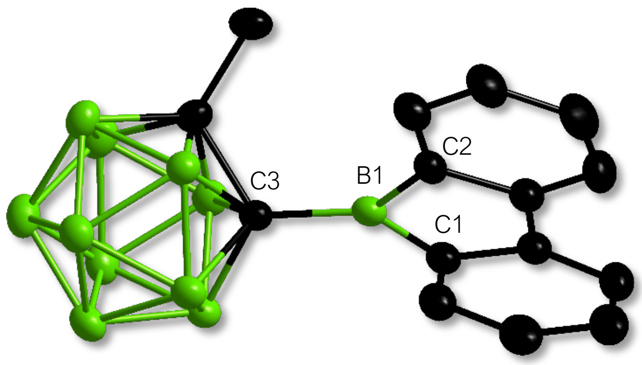


**Figure S57.** Crystallographically-derived molecular structure of **2**. Atomic displacement ellipsoids set at 50% probability. Hydrogen atoms omitted for clarity. Selected bond lengths (Å) and angles (°): B1-C1 1.567(3), B1-C2 1.595(3), C1-B1-C3 105.16(18).

**Refinement details for 2-thf:** Electron density was squeezed due to non-solvable benzene/thf solvent disorder on a symmetry axis. Disorder in the carborane cluster (residue 6) was refined with a free variable and restrained with SIMU/RIGU/ISOR. Disorder in the biphenyl backbone (residue 4) was refined with a free variable and restrained with SIMU/RIGU/ISOR. **Crystal data for 2-thf:** $C_{19}H_{29}B_{11}O$, $M_r$ = 392.33, colorless block, 0.470 x 0.240 x 0.170 mm$^3$, tetragonal space group $P4_2/n$, $a$ = 27.0865(2) Å, $b$ = 27.0865(2) Å, $c$ = 12.6873(2) Å, $V$ = 308.4(2) Å$^3$, $Z$ = 16, $\rho_{calcd}$ = 1.120 g/cm$^3$, μ = 0.428 mm$^{-1}$, $F$(000) = 3296, $T$ = 100(2) K, $R_1$ = 0.0964, $wR^2$ = 0.2815, 9061 independent reflections [2θ ≤144.234°] and 732 parameters. CCDC 2172384.

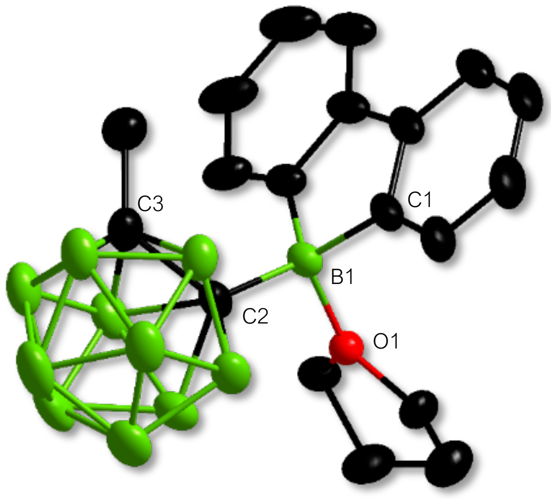


**Figure S58.** Crystallographically-derived molecular structure of **2-thf.** Atomic displacement ellipsoids set at 50% probability. Hydrogen atoms omitted for clarity. Selected bond lengths (Å) and angles (°): B1–C1 1.617(3), B1–C2 1.665(3), C2–C3 1.695(3), B1–O1 1.607(3), C2–B1–O1 105.4(16).

**Crystal data for 2-acetone:** $C_{21}H_{30}B_{11}O$, $M_r$ = 417.36, colorless block, 0.322 x 0.186 x 0.047 mm$^3$, triclinic space group *P*-1, *a* = 7.8822(1) Å, *b* = 11.7078(2) Å, *c* = 13.3889(3) Å, α= 105.252(2) °, β= 90.534(2) °, γ= 92.608(2) °, *V* = 1190.45(4) Å$^3$, *Z* = 2, $\rho_{calcd}$ = 1.164 g/cm$^3$, μ = 0.448 mm$^{-1}$, *F*(000) = 438, *T* = 100(2) K, $R_1$ = 0.0457, $wR^2$ = 0.1233, 4698 independent reflections [2θ ≤144.152°] and 311 parameters. CCDC 2172386.

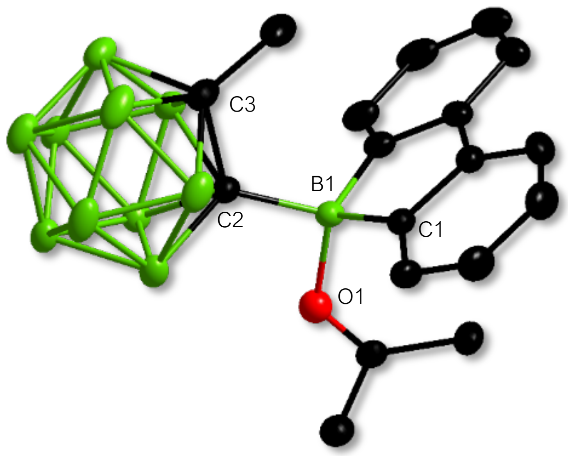


**Figure S59.** Crystallographically-derived molecular structure of **2-acetone.** Atomic displacement ellipsoids set at 50% probability. Hydrogen atoms omitted for clarity. Selected bond lengths (Å) and angles (°): B1–C1 1.617(3), B1–C2 1.666(19), C2–C3 1.673(17), B1–O1 1.594(15), C2–B1–O1 100.8(9).

**Refinement details for 2-pyridine:** Disorder in benzene refined with a free variable and restrained with SADI/SIMU/RIGU/ISOR. **Crystal data for 2-pyridine:** $C_{32}H_{38}B_{11}N$, $M_r$ = 555.54, colorless block, 0.482 x 0.234 x 0.107 mm$^3$, monoclinic space group $P2_1$, *a* = 12.3347(10) Å, *b* = 21.7120(10) Å, *c* = 13.3034(10) Å, β= 115.859(10) °, *V* = 3206.05(5) Å$^3$, *Z* = 4, $\rho_{calcd}$ = 1.151 g/cm$^3$, μ = 0.441 mm$^{-1}$, *F*(000) = 1168, *T* = 100(2) K, $R_1$ = 0.0404, $wR^2$ = 0.1032, 12634 independent reflections [2θ ≤ 144.178°] and 870 parameters. CCDC 2172395.

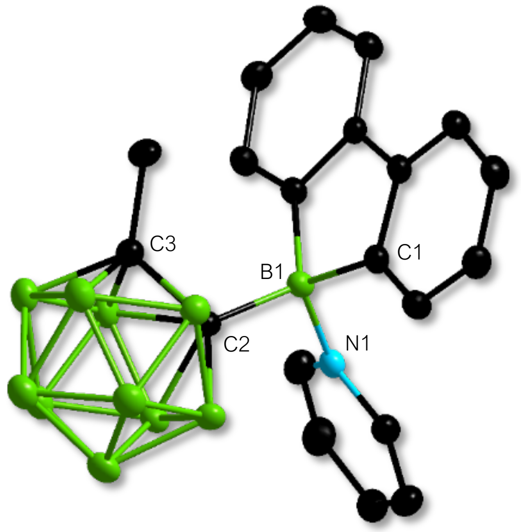


**Figure S60.** Crystallographically-derived molecular structure of **2-pyridine.** Atomic displacement ellipsoids set at 50% probability. Hydrogen atoms omitted for clarity. Selected bond lengths (Å) and angles (°): B1–C1 1.634(3), B1–C2 1.680(3), C2–C3 1.691(3), B1–N1 1.624(3), C2–B1–N1 104.6(16).

**Crystal data for 2-DMAP:** $C_{28}H_{37}B_{11}N_2$, $M_r$ = 520.50, colorless block, 0.290 x 0.256 x 0.029 mm$^3$, monoclinic space group $P2_1$, $a$ = 13.6036(3) Å, $b$ = 13.3357(2) Å, $c$ = 16.4811(2) Å, β= 96.337(2) °, $V$ = 2971.63(9) Å$^3$, $Z$ = 4, $\rho_{calcd}$ = 1.163 g/cm$^3$, μ = 0.450 mm$^{-1}$, $F$(000) = 1096, $T$ = 100(2) K, $R_1$ = 0.0516, $wR^2$ = 0.1346, 5849 independent reflections [2θ ≤144.248°] and 383 parameters. CCDC 2172390.

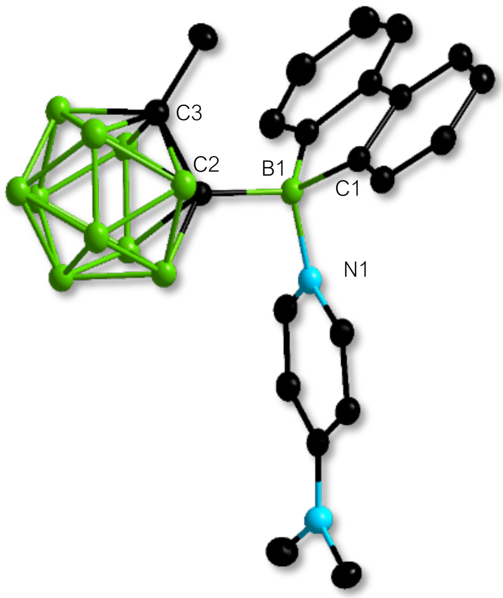


**Figure S61.** Crystallographically-derived molecular structure of **2-DMAP.** Atomic displacement ellipsoids set at 50% probability. Hydrogen atoms omitted for clarity. Selected bond lengths (Å) and angles (°): B1–C1 1.638(193), B1–C2 1.696(19), C2–C3 1.687 (19), B1–N1 1.610(19), C2–B1–N1 104.8(10).

**Refinement details for 3:** Disordered toluene (solvent) was squeezed - disorder was not possible to refine. 198 electrons were squeezed - fits well for 4 toluene (50 electrons each) molecules (Z = 4). **Crystal data for 3:** $C_{24}H_{36}B_{11}$ [$C_{21}H_{33}B_{11}$, 0.5($C_6H_6$)], $M_r$ = 443.44, colorless block, 0.292 x 0.163 x 0.101 mm$^3$, monoclinic space group $P2_1/n$, $a$ = 12.6143(2) Å, $b$ = 21.4357(3) Å, $c$ = 13.0722 (2) Å, β= 116.555(2) °, $V$ = 3161.79 (10) Å$^3$, $Z$ = 4, $\rho_{calcd}$ = 0.932 g/cm$^3$, μ = 0.0334 mm$^{-1}$, $F$(000) = 940, $T$ = 100(2) K, $R_1$ = 0.0519, $wR^2$ = 0.1383, 6233 independent reflections [2θ ≤144.252°] and 330 parameters. CCDC 2172385.

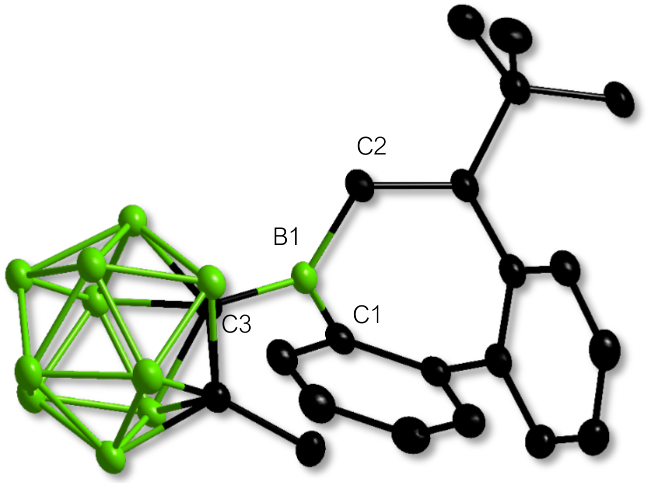


**Figure S62.** Crystallographically-derived molecular structure of **3**. Atomic displacement ellipsoids set at 50% probability. Hydrogen atoms omitted for clarity. Selected bond lengths (Å) and angles (°): B1-C1 1.555(2), B1-C2 1.567(2), B1-C3 1.614(2), C1-B1-C2 119.55(12).

**Crystal data for 4:** $C_{23}H_{29}B_{11}$, $M_r$ = 424.37, colorless block, 0.470 x 0.255 x 0.114 mm$^3$, monoclinic space group $P2_1/c$, $a$ = 15.9256(10) Å, $b$ = 12.1813(10) Å, $c$ = 24.883(2) Å, β= 97.611(10) °, $V$ = 4785.66(6) Å$^3$, $Z$ = 8, $\rho_{calcd}$ = 1.178 g/cm$^3$, μ = 0.425 mm$^{-1}$, $F$(000) = 1776, $T$ = 100(2) K, $R_1$ = 0.0435, $wR^2$ = 0.1129, 9421 independent reflections [2θ ≤144.240°] and 635 parameters. CCDC 2172393.

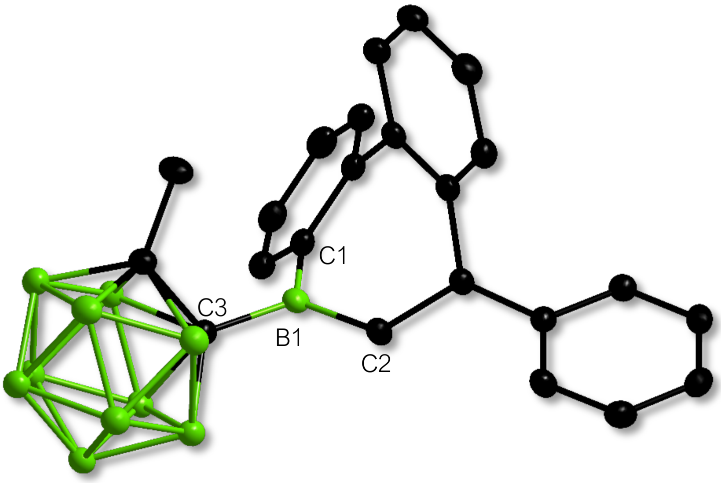


**Figure S63.** Crystallographically-derived molecular structure of **4**. Atomic displacement ellipsoids set at 50% probability. Hydrogen atoms omitted for clarity. Selected bond lengths (Å) and angles (°): B1-C1 1.566(17), B1-C2 1.563(17), B1-C3 1.604 (17), C1-B1-C2 119.53(10).

**Refinement details for 5:** Refined as a two-component inversion twin. **Crystal data for 5:** $C_{23}H_{28}B_{11}Br$, $M_r$ = 503.27, colorless plate, 0.310 x 0.190 x 0.050 mm$^3$, orthorhombic space group $P2_12_12_1$, $a$ = 8.9395(10) Å, $b$ = 9,8238(10) Å, $c$ = 29.8355(2) Å, $V$ = 2620.15(6) Å$^3$, $Z$ = 4, $\rho_{calcd}$ = 1.276 g/cm$^3$, μ = 2.190 mm$^{-1}$, $F$(000) = 1024, $T$ = 100(2) K, $R_1$ = 0.0363, $wR^2$ = 0.0891, 5155 independent reflections [2θ ≤144.244°] and 318 parameters. CCDC 2172387.

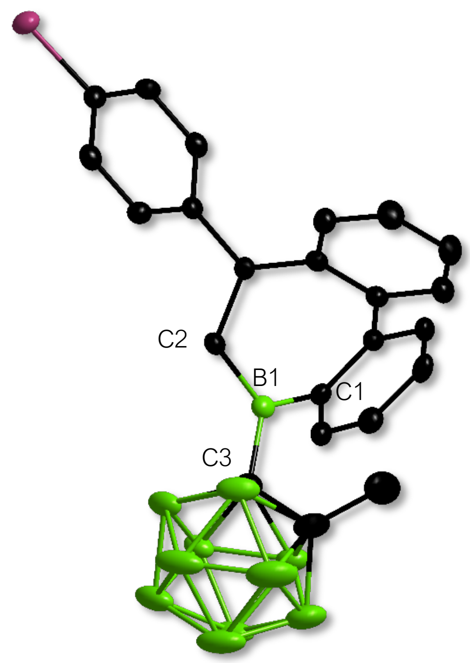


**Figure S64.** Crystallographically-derived molecular structure of **5**. Atomic displacement ellipsoids set at 50% probability. Hydrogen atoms omitted for clarity. Selected bond lengths (Å) and angles (°): B1-C1 1.555(5), B1-C2 1.560(5), B1-C3 1.606(5), C1-B1-C2 120.2(3).

**Refinement details for 5-*t*BuCN:** Refined as a two-component inversion twin. **Crystal data for 5-*t*BuCN:** $C_{28}H_{37}B_{11}BrN$, $M_r$ = 586.40, colorless plate, 0.250 x 0.100 x 0.020 mm$^3$, triclinic space group $P$-1, $a$ = 9.6639(2) Å, $b$ = 9.8598(2) Å, $c$ = 17.3492(3) Å, α= 84.800(10) °, β= 80.733(2) °, γ= 68.625(2) °, $V$ = 1518.39(6) Å$^3$, $Z$ = 2, $\rho_{calcd}$ = 1.283 g/cm$^3$, μ = 1.972 mm$^{-1}$, $F$(000) = 604, $T$ = 100(2) K, $R_1$ = 0.0316, $wR^2$ = 0.0804, 5918 independent reflections [2θ ≤144.240°] and 414 parameters. CCDC 2172388.

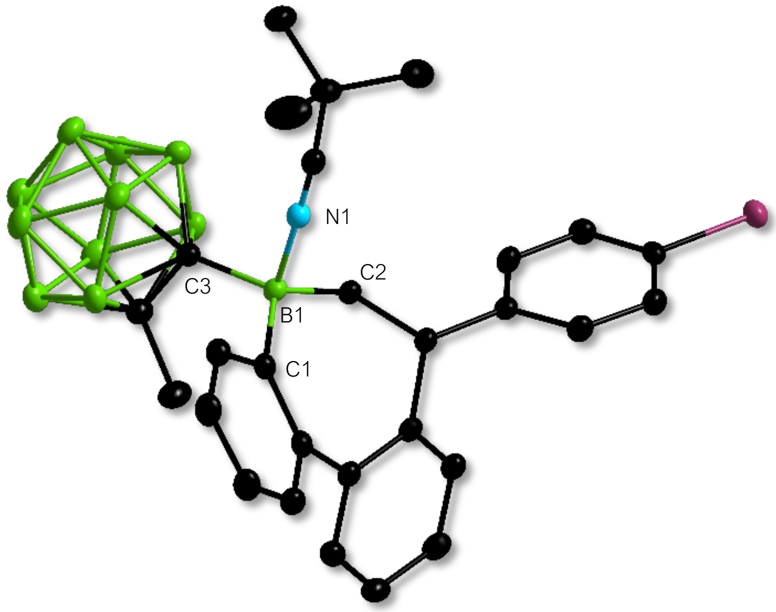


**Figure S65.** Crystallographically-derived molecular structure of **5-*t*BuCN.** Atomic displacement ellipsoids set at 50% probability. Hydrogen atoms omitted for clarity. Selected bond lengths (Å) and angles (°): B1-C1 1.629(2), B1-C2 1.616(2), B1-C3 1.689(2), B1-N1 1.608(2), C1-B1-C2 118.03(13).

**Refinement details for 6-*t*BuCN:** Disorder in *n*-butyl groups refined with PART's and a free variable, and restrained with SIMU/RIGU/ISOR. Disorder in solvent molecules was squeezed (mixture of pentane/hexane, 226 electrons). **Crystal data for 6-*t*BuCN:** $C_{26}H_{42}B_{11}N$, $M_r$ = 487.51, colorless block, 0.250 x 0.100 x 0.020 $mm^3$, triclinic space group *P*-1, *a* = 15.8288(10) Å, *b* = 16.0876(2) Å, *c* = 21.5724(10) Å, α= 94.1380(10) °, β= 92.6840(10) °, γ= 106.6770(10) °, *V* = 5235.45(8) $Å^3$, *Z* = 6, $\rho_{calcd}$ = 0.928 $g/cm^3$, μ = 0.343 $mm^{-1}$, *F*(000) = 1560, *T* = 100(2) K, $R_1$ = 0.0519, $wR^2$ = 0.1308, 20327 independent reflections [2θ ≤ 144.250°] and 1186 parameters. CCDC: 2172391.

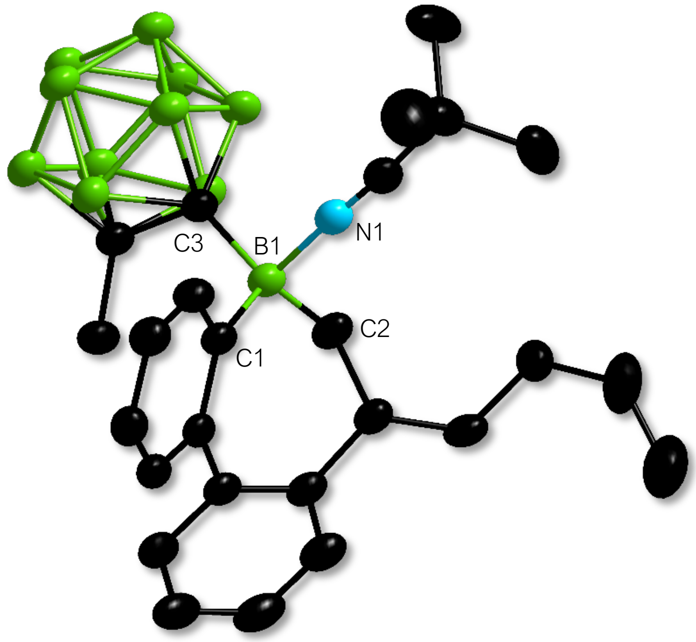


**Figure S66.** Crystallographically-derived molecular structure of **6-*t*BuCN**. Atomic displacement ellipsoids set at 50% probability. Hydrogen atoms omitted for clarity. Selected bond lengths (Å) and angles (°): B1-C1 1.622(2), B1-C2 (1.602(2), B1-C3 1.683(2), B1-N1 1.608(19), C1-B1-C2 117.42(12).

**Crystal data for 7-*t*BuCN:** $C_{23}H_{29}BBrN$, $M_r$ = 410.19, colorless plate, 0.170 x 0.110 x 0.050 $mm^3$, triclinic space group *P*-1, $a$ = 9.1163(2) Å, $b$ = 9.2445(2) Å, $c$ = 14.8961(2) Å, α= 92.922(10) °, β= 107.409(10) °, γ= 114.918(2) °, $V$ = 1063.39(4) $Å^3$, $Z$ = 2, $\rho_{calcd}$ = 1.281 g/$cm^3$, μ = 2.652 $mm^{-1}$, $F$(000) = 428, $T$ = 100(2) K, $R_1$ = 0.0267, $wR^2$ = 0.0723, 4187 independent reflections [2θ ≤ 144.258°] and 239 parameters. CCDC 2172389.

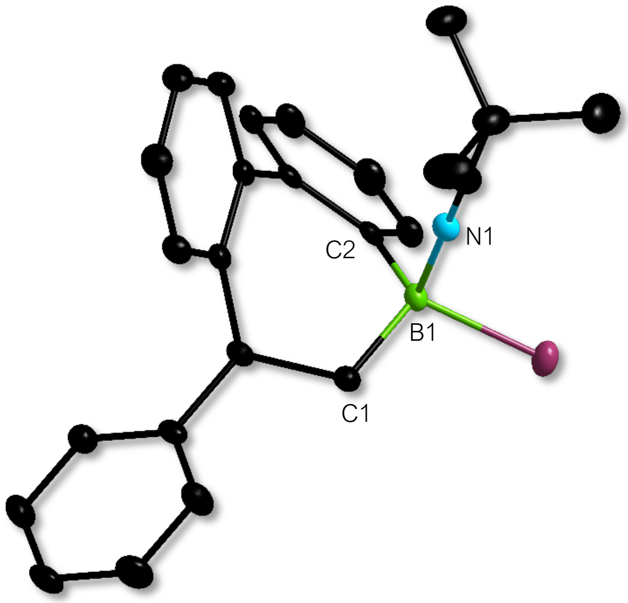


**Figure S67.** Crystallographically-derived molecular structure of **7-*t*BuCN.** Atomic displacement ellipsoids set at 50% probability. Hydrogen atoms omitted for clarity. Selected bond lengths (Å) and angles (°): B1-C1 1.608(2), B1-C14 1.611(2), B1-N1 1.590(2), C1-N1-C14 115.82(13).

**Crystal data for 8-*t*BuCN:** $C_{28}H_{33}BBrN_2$ [$C_{25}H_{30}BBrN_2$, 0.5($C_6H_6$)], $M_r$ = 488.28, colorless plate, 210 x 0.140 x 0.060 mm$^3$, monoclinic space group *C*2/c, *a* = 39.1829(4) Å, *b* = 9.7414(10) Å, *c* = 13.0827(10) Å, β= 93.923(10) °, *V* = 4981.80(8) Å$^3$, *Z* = 8, $\rho_{calcd}$ = 1.302 g/cm$^3$, μ = 2.365 mm$^{-1}$, *F*(000) = 2040, *T* = 100(2) K, $R_1$ = 0.030, $wR^2$ = 0.0876, 4870 independent reflections [2θ ≤ 144.238°] and 294 parameters. CCDC 2172392.

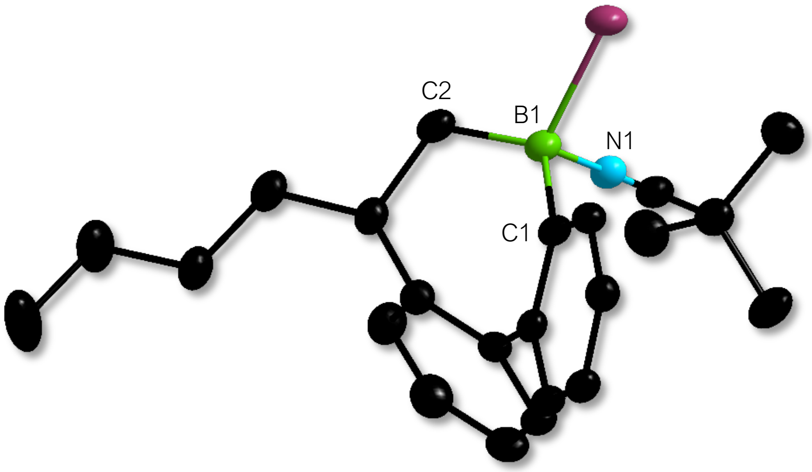


**Figure S68.** Crystallographically-derived molecular structure of **8-*t*BuCN.** Atomic displacement ellipsoids set at 50% probability. Hydrogen atoms omitted for clarity. Selected bond lengths (Å) and angles (°): B1-C1 1.606(2), B1-C2 1.609(2), B1-N1 1.587(2), C1-B1-C2 115.25(12).

**Crystal data for 9-DMAP:** $C_{28}H_{33}BBrN_2$, $M_r$ = 488.28, colorless plate, 0.210 x 0.140 x 0.060 mm$^3$, monoclinic space group *C*2/c, *a* = 39.1820(4) Å, *b* = 9.741(2) Å, *c* = 13.0827(10) Å, β= 93.923(10) °, *V* = 4981.80(8) Å$^3$, *Z* = 8, $\rho_{calcd}$ = 1.302 g/cm$^3$, μ = 2.365 mm$^{-1}$, *F*(000) = 2040, *T* = 100(2) K, $R_1$ = 0.0330, $wR^2$ = 0.0876, 4870 independent reflections [2θ ≤144.238°] and 294 parameters. CCDC 2172394.

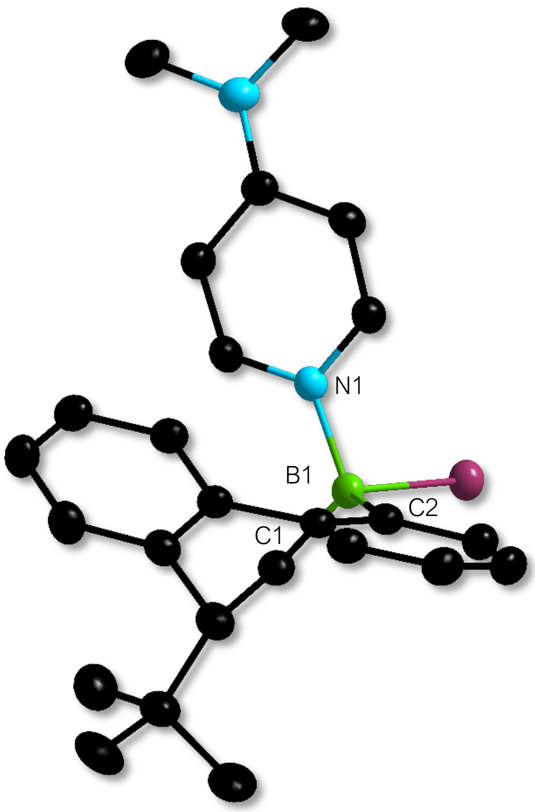


**Figure S69.** Crystallographically-derived molecular structure of **9-DMAP**. Atomic displacement ellipsoids set at 50% probability. Hydrogen atoms omitted for clarity. Selected bond lengths (Å) and angles (°): B1-C11.612(3), B1-C2 1.610(3), B1-N1 1.586(3), C1-B1-C2 113.88 (17).

# 7. Computational details

## 7.1 Electronic properties

All calculations were performed at the M062X/6-31G** level of theory.[7,8]

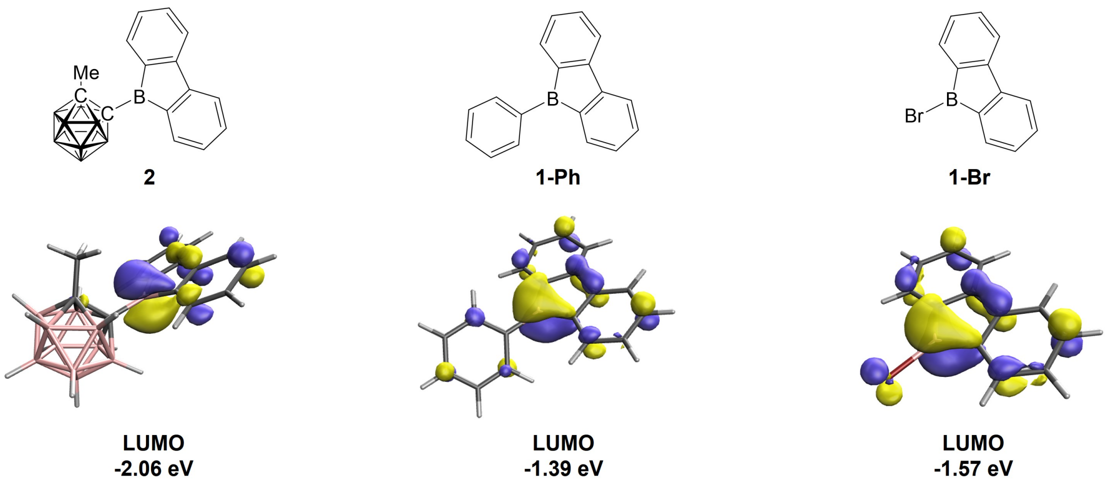


$NICS(1)_{zz}$ = 7.1667 ppm  $NICS(1)_{zz}$ = 6.0149 ppm  $NICS(1)_{zz}$ = 6.0425 ppm

NPA charge of boron:

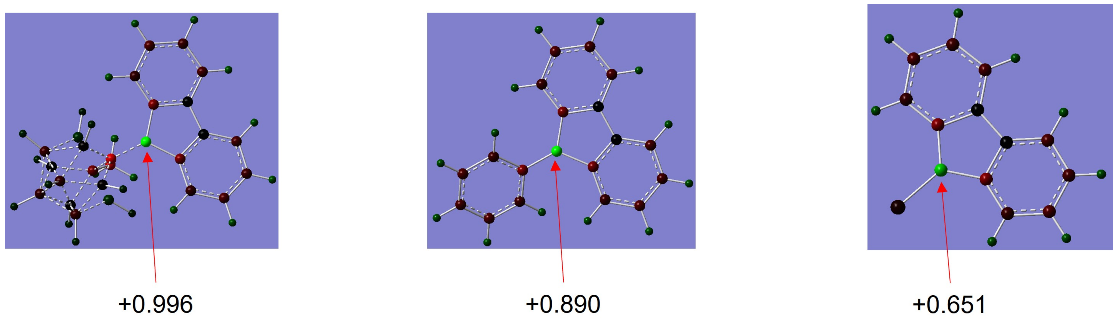


**Figure S70.** Overview of calculated LUMO energies, $NICS(1)_{zz}$ values and NPA charges of **2**, **1Ph** and **1Br**.

The following reaction was used to determine the fluoride ion affinity (FIA) of **2**, **1Ph** and **1Br**:

$$[Me_3BF]^- + LA \longrightarrow Me_3B + [LAF]^-$$

FIA (**2**) = -46.0 kcal/mol

FIA (**1Ph**) = -22.2 kcal/mol

FIA (**1Br**) = -31.8 kcal/mol

## 7.2 Mechanistic studies

We carried out systematic DFT calculations using M062X/6-31G**[7,8] in order to better understand the experimentally observed olefin insertion reactions. We calculated the energy profiles for the insertion reactions of various olefins (substituted by Ph, *t*Bu and *n*Bu) with carboranyl-, phenyl- and bromo-substituted borafluorenes. To examine the regioselectivity, we also calculated and compared the 1,2- and 2,1-insertions for carboranyl-substituted borafluorene.

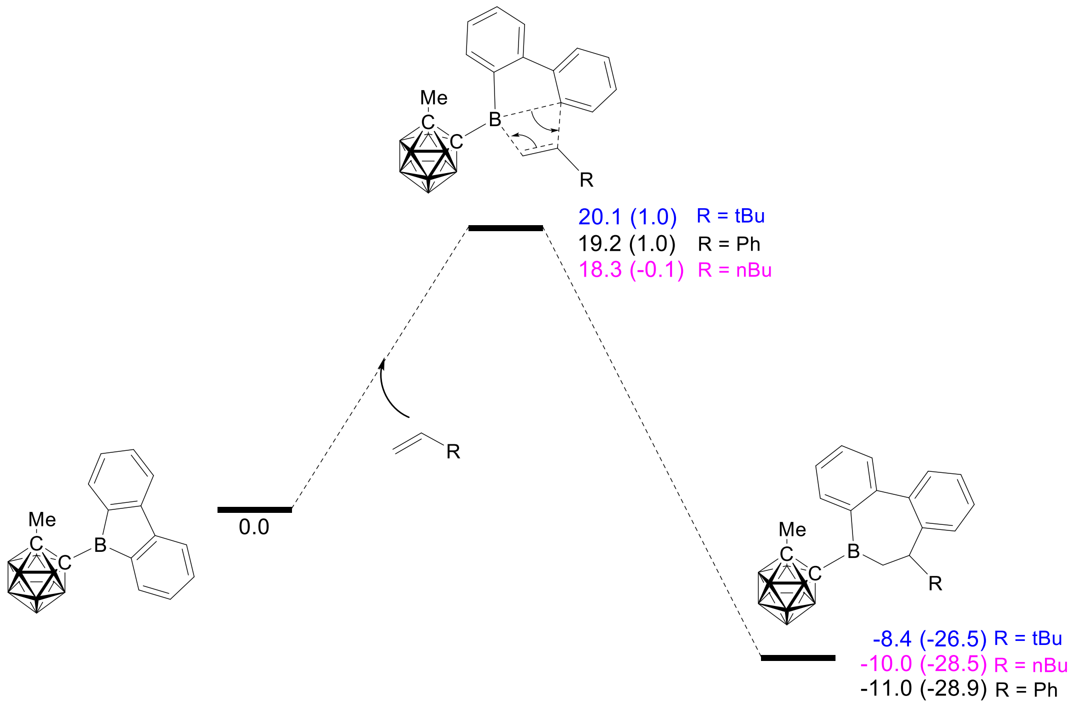


**Figure S71.** Energy profiles calculated for olefin 1,2-insertion into a B-C bond of the carboranyl-substituted borafluorene **2'**. Relative free energies and electronic energies (in parentheses) are given in kcal/mol.

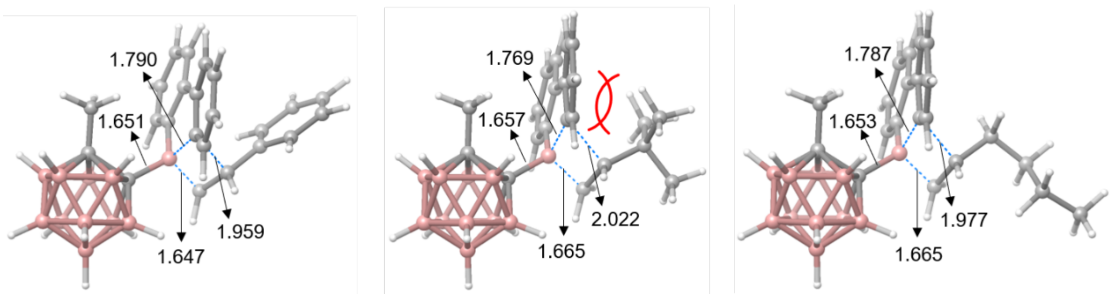


**Figure S72.** Calculated transition state structures for the olefin 1,2-insertion into a B-C bond of the carboranyl-substituted borafluorene **2'**. From left to right: R = Ph, *t*Bu, and *n*Bu.

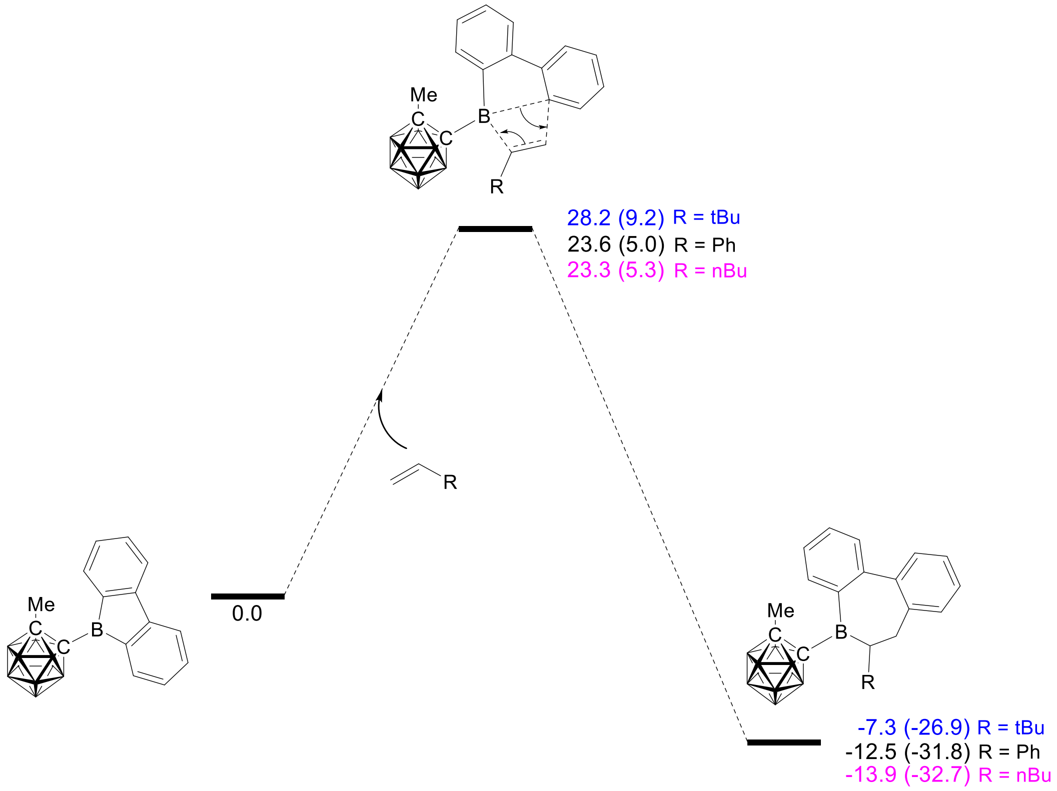


**Figure S73.** Energy profile calculated for olefin 2,1-insertion into a B-C bond of the carboranyl-substituted borafluorene **2'**. Relative free energies and electronic energies (in parentheses) are given in kcal/mol.

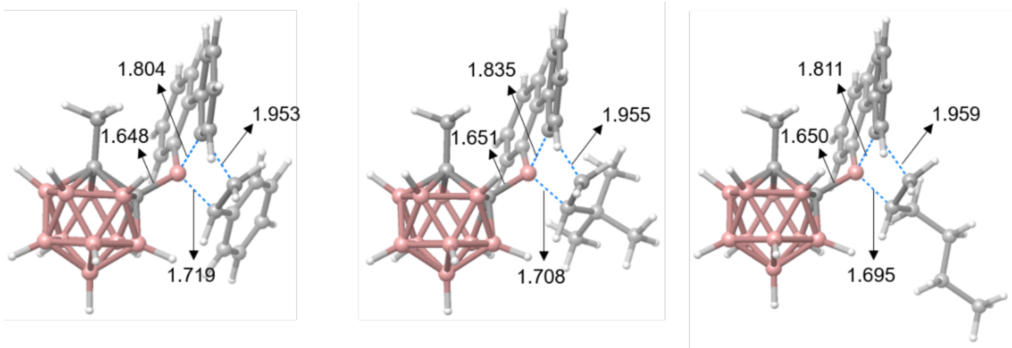


**Figure S74.** Calculated transition state structures for the olefin 2,1-insertion into a B-C bond of the carboranyl-substituted borafluorene **2'**.

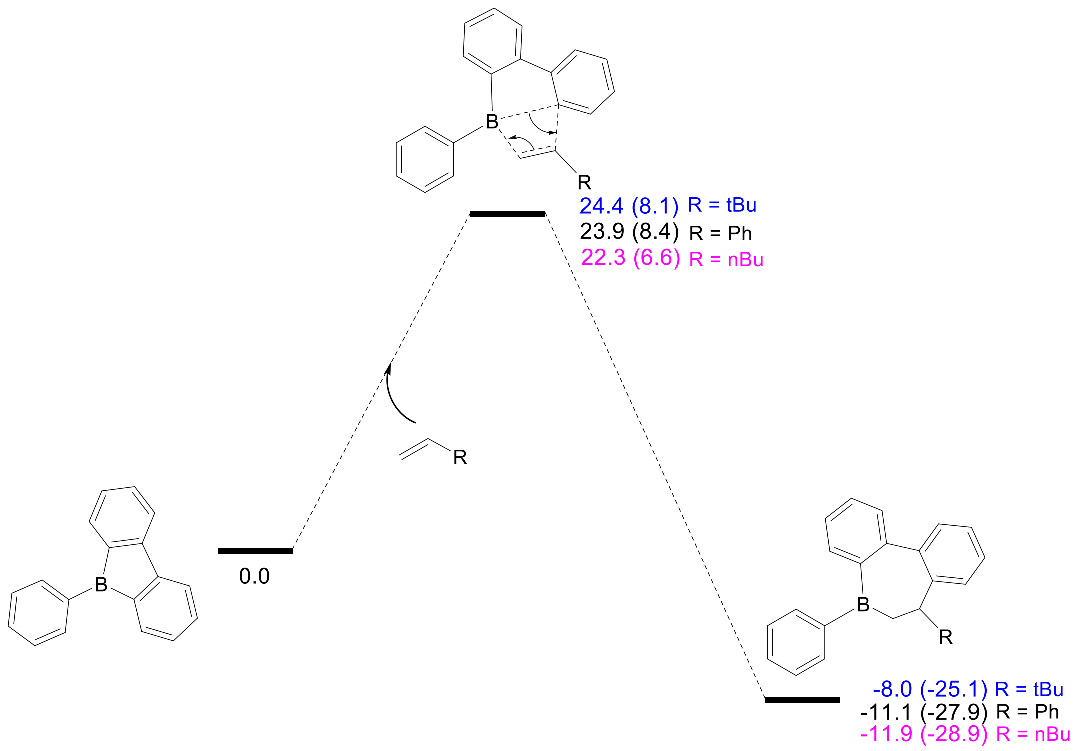


**Figure S75.** Energy profile calculated for olefin 1,2-insertion into a B-C bond of the phenyl-substituted borafluorene **1Ph'**. Relative free energies and electronic energies (in parenthesis) are given in kcal/mol.

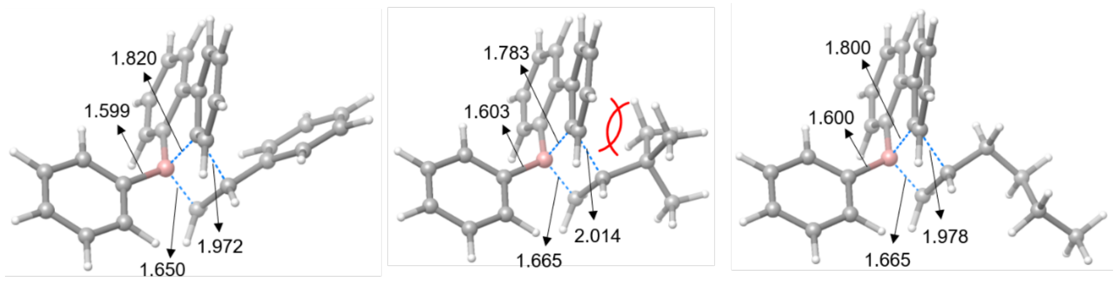


**Figure S76.** Calculated transition state structures for the olefin 1,2-insertion into a B-C bond of the phenyl-substituted borafluorene **1Ph'**.

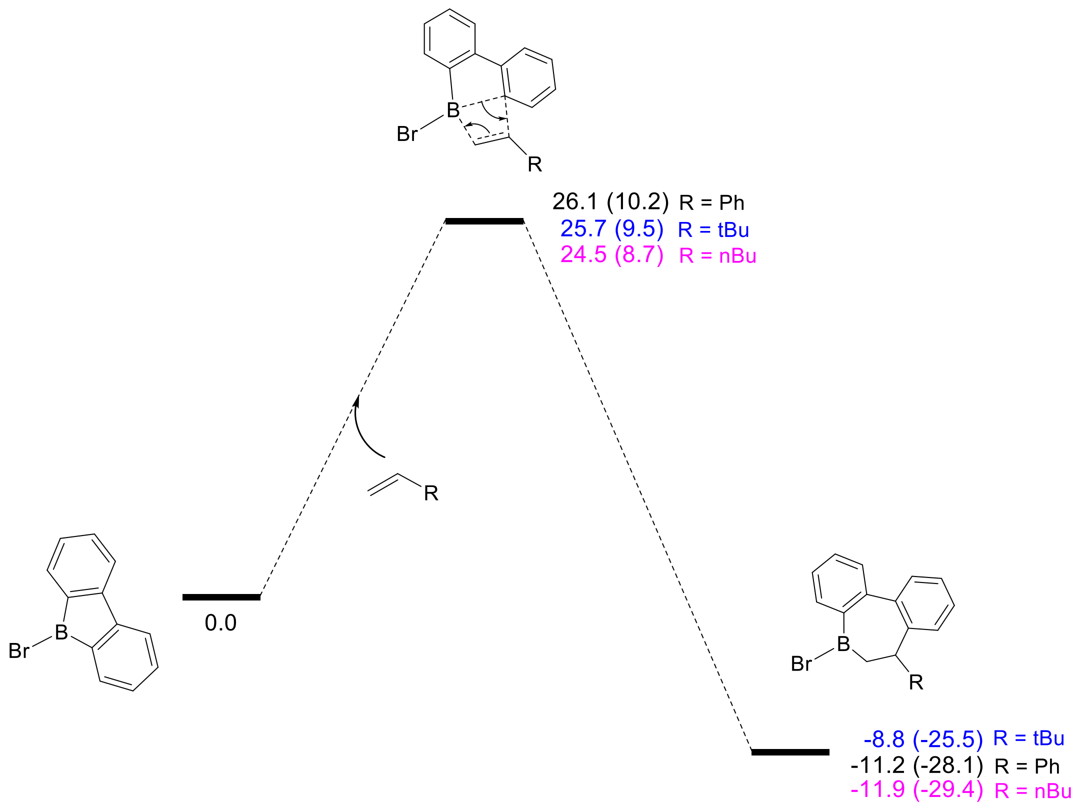


**Figure S77.** Energy profile calculated for olefin 1,2-insertion into a B-C bond of the bromo-substituted borafluorene **1Br'**. Relative free energies and electronic energies (in parenthesis) are given in kcal/mol.

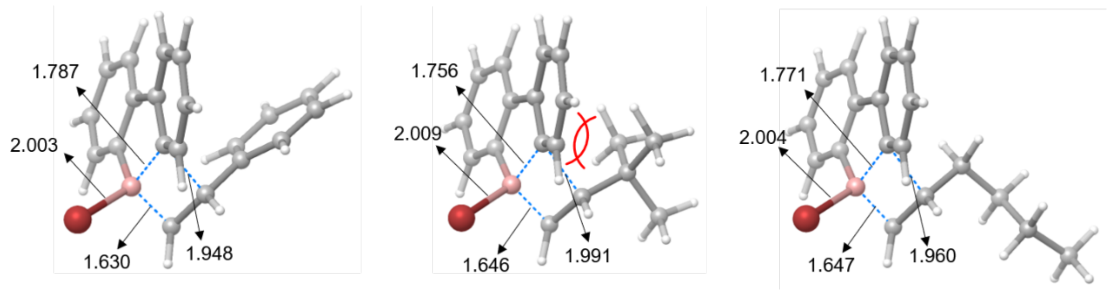


**Figure S78.** Calculated transition state structures for the olefin 1,2-insertion into a B-C bond of carboranyl-substituted borafluorene.

7.3 Cartesian coordinates

47

E (2') = -857.493869997 a.u.

| | | | |
|---|---|---|---|
| H | 1.315185000 | 2.319144000 | 0.708407000 |
| B | 1.953736000 | -1.430846000 | 0.271507000 |
| C | -2.718525000 | -0.743489000 | 0.002005000 |
| H | 1.315136000 | -2.319207000 | 0.708248000 |
| H | 1.310263000 | -1.479881000 | -2.189047000 |
| B | -0.453204000 | -0.000017000 | -0.215920000 |
| C | 1.139232000 | -0.000003000 | -0.208343000 |
| B | 1.966850000 | 0.886689000 | -1.402710000 |
| H | 3.962484000 | -2.470085000 | -0.924453000 |
| C | -1.406133000 | 1.242707000 | -0.186628000 |
| B | 3.427572000 | -0.882523000 | 1.052955000 |
| H | 5.567321000 | -0.000039000 | -0.096245000 |
| H | 3.996184000 | 0.000035000 | -2.734479000 |
| C | 1.978463000 | -0.000044000 | 1.199310000 |
| B | 1.953749000 | 1.430799000 | 0.271575000 |
| C | -2.320961000 | 3.481128000 | -0.226371000 |
| H | -2.177409000 | 4.551017000 | -0.333379000 |
| H | 3.962509000 | 2.470106000 | -0.924300000 |
| B | 3.462531000 | -1.439388000 | -0.626717000 |
| H | 3.791320000 | 1.472918000 | 2.011276000 |
| B | 3.475652000 | 0.000033000 | -1.671315000 |
| C | -3.595070000 | 2.972214000 | -0.002041000 |
| H | -4.439343000 | 3.650345000 | 0.074577000 |
| B | 1.966844000 | -0.886628000 | -1.402743000 |
| C | -3.805923000 | 1.592409000 | 0.108333000 |
| H | -4.808820000 | 1.204675000 | 0.260660000 |
| B | 3.462543000 | 1.439392000 | -0.626641000 |
| H | 3.791325000 | -1.473086000 | 2.011181000 |

| | | | |
|---|---|---|---|
| C | -2.718501000 | 0.743528000 | 0.002011000 |
| B | 3.427573000 | 0.882431000 | 1.053000000 |
| H | 1.310226000 | 1.479993000 | -2.188939000 |
| C | -1.406168000 | -1.242711000 | -0.186608000 |
| B | 4.384442000 | -0.000016000 | -0.146950000 |
| C | -3.805976000 | -1.592335000 | 0.108298000 |
| H | -4.808864000 | -1.204572000 | 0.260606000 |
| C | -3.595166000 | -2.972148000 | -0.002080000 |
| H | -4.439465000 | -3.650249000 | 0.074518000 |
| C | -2.321070000 | -3.481102000 | -0.226387000 |
| H | -2.177552000 | -4.550995000 | -0.333398000 |
| C | -1.223961000 | -2.617660000 | -0.317061000 |
| H | -0.239295000 | -3.029832000 | -0.508824000 |
| H | -0.239205000 | 3.029790000 | -0.508855000 |
| C | -1.223883000 | 2.617651000 | -0.317076000 |
| C | 1.175632000 | -0.000066000 | 2.488798000 |
| H | 0.545254000 | 0.889607000 | 2.549742000 |
| H | 0.545907000 | -0.890170000 | 2.550123000 |
| H | 1.863456000 | 0.000377000 | 3.335421000 |

32

E (1Ph’) = -718.324781043 a.u.

| | | | |
|---|---|---|---|
| C | -1.943623000 | -0.743532000 | -0.028678000 |
| B | 0.352331000 | -0.000224000 | -0.000090000 |
| C | -0.617194000 | 1.237343000 | 0.038730000 |
| C | -1.498058000 | 3.483012000 | 0.180459000 |
| H | -1.336111000 | 4.553934000 | 0.247489000 |
| C | -2.794726000 | 2.980338000 | 0.152031000 |
| H | -3.636773000 | 3.664559000 | 0.193678000 |
| C | -3.029555000 | 1.601811000 | 0.081577000 |
| H | -4.047568000 | 1.222713000 | 0.074589000 |
| C | -1.943409000 | 0.743948000 | 0.028427000 |
| C | -0.617583000 | -1.237392000 | -0.038719000 |

| | | | |
|---|---|---|---|
| C | -3.030080000 | -1.601007000 | -0.081778000 |
| H | -4.047922000 | -1.221448000 | -0.074945000 |
| C | -2.795781000 | -2.979640000 | -0.151971000 |
| H | -3.638070000 | -3.663558000 | -0.193635000 |
| C | -1.499286000 | -3.482783000 | -0.180108000 |
| H | -1.337658000 | -4.553765000 | -0.246939000 |
| C | -0.407179000 | -2.608800000 | -0.130024000 |
| H | 0.603233000 | -3.007133000 | -0.174627000 |
| H | 0.604302000 | 3.006559000 | 0.175209000 |
| C | -0.406256000 | 2.608648000 | 0.130398000 |
| C | 1.901864000 | -0.000227000 | -0.000164000 |
| C | 2.628223000 | 1.013195000 | -0.648491000 |
| C | 2.628043000 | -1.013723000 | 0.648230000 |
| C | 4.018975000 | 1.008904000 | -0.662120000 |
| H | 2.090448000 | 1.801562000 | -1.168771000 |
| C | 4.018799000 | -1.009585000 | 0.662106000 |
| H | 2.090086000 | -1.802049000 | 1.168397000 |
| C | 4.715280000 | -0.000369000 | 0.000052000 |
| H | 4.561525000 | 1.792562000 | -1.181398000 |
| H | 4.561187000 | -1.793299000 | 1.181469000 |
| H | 5.800939000 | -0.000372000 | 0.000184000 |

22

E (1Br’) = -3058.28282040 a.u.

| | | | |
|---|---|---|---|
| C | 1.442223000 | -0.745676000 | -0.000009000 |
| B | -0.817300000 | 0.000024000 | -0.000015000 |
| C | 0.117286000 | 1.241906000 | 0.000016000 |
| C | 0.976483000 | 3.490252000 | 0.000006000 |
| H | 0.811327000 | 4.562527000 | -0.000054000 |
| C | 2.275777000 | 2.991127000 | -0.000011000 |
| H | 3.113694000 | 3.681536000 | -0.000082000 |
| C | 2.522290000 | 1.612232000 | 0.000005000 |
| H | 3.543100000 | 1.241339000 | -0.000011000 |

| | | | |
|---|---|---|---|
| C | 1.442266000 | 0.745595000 | -0.000008000 |
| C | 0.117215000 | -1.241912000 | 0.000014000 |
| C | 2.522199000 | -1.612374000 | 0.000006000 |
| H | 3.543029000 | -1.241538000 | -0.000009000 |
| C | 2.275608000 | -2.991255000 | -0.000010000 |
| H | 3.113485000 | -3.681712000 | -0.000080000 |
| C | 0.976285000 | -3.490306000 | 0.000006000 |
| H | 0.811068000 | -4.562572000 | -0.000053000 |
| C | -0.111510000 | -2.610801000 | 0.000036000 |
| H | -1.129205000 | -2.992392000 | 0.000104000 |
| H | -1.129035000 | 2.992456000 | 0.000106000 |
| C | -0.111362000 | 2.610807000 | 0.000038000 |
| Br | -2.721701000 | 0.000076000 | -0.000011000 |

63

E (2'-Ph_1,2-insert-ts) = -1166.99821311 a.u.

| | | | |
|---|---|---|---|
| H | 1.916185000 | 1.994229000 | 1.089449000 |
| B | 2.859421000 | -1.400003000 | -0.407563000 |
| C | -1.328221000 | -1.072126000 | 1.113825000 |
| H | 2.304470000 | -2.435309000 | -0.308079000 |
| H | 2.334900000 | -0.786148000 | -2.809401000 |
| B | 0.284155000 | -0.193751000 | -0.597535000 |
| C | 1.915846000 | 0.033248000 | -0.485216000 |
| B | 2.705312000 | 1.284928000 | -1.317372000 |
| H | 4.982080000 | -1.880967000 | -1.762277000 |
| C | -0.610591000 | 1.067340000 | 0.303266000 |
| B | 4.273537000 | -0.999481000 | 0.566997000 |
| H | 6.351958000 | 0.351889000 | -0.167808000 |
| H | 4.840224000 | 1.013193000 | -2.760357000 |
| C | 2.750485000 | -0.320160000 | 0.903703000 |
| B | 2.613122000 | 1.303880000 | 0.439397000 |
| C | -0.910607000 | 3.123308000 | 1.568534000 |
| H | -0.745472000 | 4.190131000 | 1.674273000 |

| | | | |
|---|---|---|---|
| H | 4.534242000 | 2.820511000 | -0.308890000 |
| B | 4.391989000 | -1.025206000 | -1.194823000 |
| H | 4.412528000 | 0.985998000 | 2.187478000 |
| B | 4.303493000 | 0.654002000 | -1.767238000 |
| C | -1.637495000 | 2.426160000 | 2.539292000 |
| H | -2.040249000 | 2.958125000 | 3.395430000 |
| B | 2.880375000 | -0.387517000 | -1.839631000 |
| C | -1.827330000 | 1.054328000 | 2.426881000 |
| H | -2.353755000 | 0.510044000 | 3.205202000 |
| B | 4.130052000 | 1.707979000 | -0.352300000 |
| H | 4.671284000 | -1.819088000 | 1.322482000 |
| C | -1.309512000 | 0.373358000 | 1.325015000 |
| B | 4.121398000 | 0.676318000 | 1.083070000 |
| H | 2.021791000 | 2.040651000 | -1.928962000 |
| C | -0.435880000 | -1.446201000 | 0.094175000 |
| B | 5.174822000 | 0.272363000 | -0.273623000 |
| C | -2.125511000 | -2.011513000 | 1.774602000 |
| H | -2.827622000 | -1.702022000 | 2.543550000 |
| C | -2.029361000 | -3.347577000 | 1.410537000 |
| H | -2.639558000 | -4.091645000 | 1.912299000 |
| C | -1.161781000 | -3.736970000 | 0.384187000 |
| H | -1.100433000 | -4.783479000 | 0.102091000 |
| C | -0.385571000 | -2.792045000 | -0.281671000 |
| H | 0.263848000 | -3.106690000 | -1.094739000 |
| H | 0.140132000 | 3.005679000 | -0.292434000 |
| C | -0.407051000 | 2.449083000 | 0.466026000 |
| C | 1.976143000 | -0.766044000 | 2.131538000 |
| H | 1.169665000 | -0.065023000 | 2.356711000 |
| H | 1.551066000 | -1.760151000 | 1.985549000 |
| H | 2.662776000 | -0.793043000 | 2.979095000 |
| C | -0.247508000 | -0.098851000 | -2.153460000 |
| H | -0.676224000 | -1.019853000 | -2.536750000 |

| | | | |
|---|---|---|---|
| H | 0.463588000 | 0.350971000 | -2.840155000 |
| C | -1.165925000 | 0.865206000 | -1.564819000 |
| H | -0.929404000 | 1.899322000 | -1.793861000 |
| C | -2.619657000 | 0.620813000 | -1.385500000 |
| C | -3.181815000 | -0.653671000 | -1.495586000 |
| C | -3.448088000 | 1.714261000 | -1.099477000 |
| C | -4.553110000 | -0.827012000 | -1.325548000 |
| H | -2.553621000 | -1.514667000 | -1.698101000 |
| C | -4.815272000 | 1.541015000 | -0.944616000 |
| H | -3.004311000 | 2.700351000 | -0.984709000 |
| C | -5.370484000 | 0.265443000 | -1.055078000 |
| H | -4.980391000 | -1.820823000 | -1.406645000 |
| H | -5.449036000 | 2.395513000 | -0.732312000 |
| H | -6.439267000 | 0.126393000 | -0.928692000 |

63

E (2’-Ph_1,2-insert-pdt) = -1167.04585159 a.u.

| | | | |
|---|---|---|---|
| H | -2.222253000 | 1.855791000 | 1.377472000 |
| B | -2.901937000 | -1.071635000 | -1.002508000 |
| C | 1.379157000 | 0.138008000 | -1.255614000 |
| H | -2.248057000 | -1.427145000 | -1.915262000 |
| H | -2.355019000 | -2.861015000 | 0.735153000 |
| B | -0.502843000 | -0.384729000 | 0.429381000 |
| C | -2.106361000 | -0.419502000 | 0.377178000 |
| B | -2.992545000 | -0.614364000 | 1.823193000 |
| H | -4.950937000 | -2.598803000 | -0.940771000 |
| C | 1.631008000 | 1.704663000 | 0.718949000 |
| B | -4.347271000 | -0.092617000 | -1.204871000 |
| H | -6.527367000 | -0.219194000 | 0.169128000 |
| H | -5.064181000 | -2.125561000 | 2.080448000 |
| C | -2.893156000 | 0.593484000 | -0.650914000 |
| B | -2.894905000 | 0.949566000 | 1.025803000 |
| C | 1.741121000 | 4.105118000 | 0.342942000 |

| | | | |
|---|---|---|---|
| H | 1.825747000 | 5.109069000 | 0.745647000 |
| H | -4.959263000 | 0.902273000 | 2.545926000 |
| B | -4.440817000 | -1.671434000 | -0.410878000 |
| H | -4.666284000 | 2.261423000 | -0.228239000 |
| B | -4.503073000 | -1.387584000 | 1.344161000 |
| C | 1.639124000 | 3.903865000 | -1.028991000 |
| H | 1.641448000 | 4.748370000 | -1.710171000 |
| B | -2.976745000 | -1.864941000 | 0.573639000 |
| C | 1.528847000 | 2.608694000 | -1.526342000 |
| H | 1.434133000 | 2.441780000 | -2.596044000 |
| B | -4.447114000 | 0.367046000 | 1.622833000 |
| H | -4.678127000 | 0.175452000 | -2.308471000 |
| C | 1.513952000 | 1.502078000 | -0.671254000 |
| B | -4.340665000 | 1.156885000 | 0.042429000 |
| H | -2.377759000 | -0.768853000 | 2.821612000 |
| C | 0.374349000 | -0.745316000 | -0.807511000 |
| B | -5.347467000 | -0.287004000 | 0.240563000 |
| C | 2.237728000 | -0.254726000 | -2.283512000 |
| H | 3.008343000 | 0.433268000 | -2.618864000 |
| C | 2.143684000 | -1.526450000 | -2.840815000 |
| H | 2.837852000 | -1.827238000 | -3.618891000 |
| C | 1.166444000 | -2.411117000 | -2.395505000 |
| H | 1.090892000 | -3.405054000 | -2.823736000 |
| C | 0.278207000 | -2.011471000 | -1.401885000 |
| H | -0.487137000 | -2.705917000 | -1.064770000 |
| H | 1.834098000 | 3.163914000 | 2.273566000 |
| C | 1.739945000 | 3.008710000 | 1.201829000 |
| C | -2.057224000 | 1.494565000 | -1.544966000 |
| H | -1.354167000 | 2.090675000 | -0.956773000 |
| H | -1.495426000 | 0.905676000 | -2.272451000 |
| H | -2.725631000 | 2.173491000 | -2.076427000 |
| C | 0.183621000 | -0.011666000 | 1.794601000 |

| | | | |
|---|---|---|---|
| H | 0.148232000 | -0.909029000 | 2.433445000 |
| H | -0.429836000 | 0.721860000 | 2.330172000 |
| C | 1.621283000 | 0.533848000 | 1.691814000 |
| H | 1.878341000 | 0.947413000 | 2.675646000 |
| C | 2.714360000 | -0.484408000 | 1.371092000 |
| C | 4.009774000 | -0.010121000 | 1.135005000 |
| C | 2.498550000 | -1.861372000 | 1.330403000 |
| C | 5.054515000 | -0.882864000 | 0.856923000 |
| H | 4.193271000 | 1.061240000 | 1.160772000 |
| C | 3.542351000 | -2.740212000 | 1.045721000 |
| H | 1.506897000 | -2.267922000 | 1.500270000 |
| C | 4.823192000 | -2.256018000 | 0.806721000 |
| H | 6.050476000 | -0.490764000 | 0.676308000 |
| H | 3.346892000 | -3.807190000 | 1.007668000 |
| H | 5.634907000 | -2.940712000 | 0.583703000 |

65

E (2’-nBu_1,2-insert-ts) = -1093.21819506 a.u.

| | | | |
|---|---|---|---|
| H | -1.498631000 | -1.064793000 | 2.229498000 |
| B | -2.659820000 | 0.290175000 | -1.163509000 |
| C | 1.041176000 | 2.230052000 | -0.269971000 |
| H | -2.311434000 | 1.254696000 | -1.742703000 |
| H | -1.558147000 | -1.540792000 | -2.540656000 |
| B | 0.063342000 | -0.073749000 | -0.444803000 |
| C | -1.495111000 | -0.586319000 | -0.242970000 |
| B | -1.906329000 | -2.233296000 | -0.084684000 |
| H | -4.518867000 | -0.731652000 | -2.596916000 |
| C | 0.854921000 | 0.274310000 | 1.118518000 |
| B | -4.150136000 | 0.249740000 | -0.229445000 |
| H | -5.802814000 | -1.720376000 | 0.010447000 |
| H | -3.730466000 | -3.460169000 | -1.448680000 |
| C | -2.628210000 | 0.337449000 | 0.536656000 |
| B | -2.148248000 | -1.124196000 | 1.251927000 |

| | | | |
|---|---|---|---|
| C | 1.126621000 | 0.369363000 | 3.536897000 |
| H | 1.102745000 | -0.133152000 | 4.497868000 |
| H | -3.624555000 | -3.182441000 | 1.599239000 |
| B | -3.923007000 | -0.863253000 | -1.581961000 |
| H | -4.278045000 | -0.230835000 | 2.288825000 |
| B | -3.461453000 | -2.439481000 | -0.910998000 |
| C | 1.462721000 | 1.722785000 | 3.460367000 |
| H | 1.694358000 | 2.275750000 | 4.365001000 |
| B | -2.228781000 | -1.362517000 | -1.581316000 |
| C | 1.492517000 | 2.363029000 | 2.228313000 |
| H | 1.722775000 | 3.422574000 | 2.168721000 |
| B | -3.403347000 | -2.287900000 | 0.855144000 |
| H | -4.811387000 | 1.230899000 | -0.212679000 |
| C | 1.184437000 | 1.653444000 | 1.066621000 |
| B | -3.832699000 | -0.622102000 | 1.264497000 |
| H | -1.019612000 | -3.017443000 | -0.007180000 |
| C | 0.391101000 | 1.334269000 | -1.132591000 |
| B | -4.655596000 | -1.436907000 | -0.070716000 |
| C | 1.469737000 | 3.488318000 | -0.705753000 |
| H | 1.987634000 | 4.165224000 | -0.032189000 |
| C | 1.239169000 | 3.854001000 | -2.024718000 |
| H | 1.561262000 | 4.827268000 | -2.380729000 |
| C | 0.605335000 | 2.967476000 | -2.902603000 |
| H | 0.433496000 | 3.263783000 | -3.932543000 |
| C | 0.201305000 | 1.710288000 | -2.465247000 |
| H | -0.279058000 | 1.024094000 | -3.159289000 |
| H | 0.621187000 | -1.403470000 | 2.463892000 |
| C | 0.838966000 | -0.342625000 | 2.379478000 |
| C | -2.183632000 | 1.629183000 | 1.200515000 |
| H | -1.563742000 | 1.424610000 | 2.076320000 |
| H | -1.619693000 | 2.249012000 | 0.500832000 |
| H | -3.072166000 | 2.177651000 | 1.516893000 |

| | | | |
|---|---|---|---|
| C | 0.952644000 | -1.218460000 | -1.263301000 |
| H | 1.403102000 | -0.828895000 | -2.171586000 |
| H | 0.431077000 | -2.160965000 | -1.387332000 |
| C | 1.715943000 | -1.068232000 | -0.050326000 |
| H | 1.566160000 | -1.861234000 | 0.676319000 |
| C | 3.110181000 | -0.482034000 | -0.106565000 |
| H | 3.467761000 | -0.276810000 | 0.907779000 |
| H | 3.073004000 | 0.468119000 | -0.649958000 |
| C | 4.064218000 | -1.448641000 | -0.823625000 |
| H | 5.055243000 | -0.980252000 | -0.850935000 |
| H | 3.748621000 | -1.571811000 | -1.866569000 |
| C | 4.173240000 | -2.821242000 | -0.160041000 |
| H | 3.206787000 | -3.337447000 | -0.217895000 |
| H | 4.394639000 | -2.691829000 | 0.907280000 |
| C | 5.246084000 | -3.689950000 | -0.810806000 |
| H | 6.229766000 | -3.217387000 | -0.732959000 |
| H | 5.306550000 | -4.673127000 | -0.338614000 |
| H | 5.032639000 | -3.840141000 | -1.873281000 |

65

E (2’-nBu_1,2-insert-pdt) = -1093.26336669 a.u.

| | | | |
|---|---|---|---|
| H | -1.641332000 | 0.324405000 | 1.883918000 |
| B | -2.630910000 | -0.585732000 | -1.710954000 |
| C | 0.974928000 | 1.942967000 | -0.277970000 |
| H | -2.229172000 | -0.102776000 | -2.712535000 |
| H | -1.345983000 | -2.787263000 | -1.712631000 |
| B | -0.037165000 | -0.330827000 | -0.711112000 |
| C | -1.549617000 | -0.702526000 | -0.391955000 |
| B | -1.904362000 | -1.995373000 | 0.668656000 |
| H | -4.334845000 | -2.378822000 | -2.387780000 |
| C | 1.444954000 | 0.233007000 | 1.547248000 |
| B | -4.196388000 | -0.196789000 | -0.997159000 |
| H | -5.797716000 | -1.790281000 | 0.246116000 |

| | | | |
|---|---|---|---|
| H | -3.572901000 | -3.897908000 | 0.162127000 |
| C | -2.760413000 | 0.427529000 | -0.344991000 |
| B | -2.262128000 | -0.323605000 | 1.114295000 |
| C | 0.788894000 | 0.756101000 | 3.838902000 |
| H | 0.700519000 | 0.444940000 | 4.874504000 |
| H | -3.721216000 | -1.921855000 | 2.498804000 |
| B | -3.817239000 | -1.864280000 | -1.455825000 |
| H | -4.516014000 | 0.835471000 | 1.329724000 |
| B | -3.374732000 | -2.738131000 | 0.030143000 |
| C | 0.379019000 | 2.026003000 | 3.447541000 |
| H | -0.033743000 | 2.720181000 | 4.171889000 |
| B | -2.117886000 | -2.158184000 | -1.071676000 |
| C | 0.485458000 | 2.393604000 | 2.111475000 |
| H | 0.141458000 | 3.373085000 | 1.789114000 |
| B | -3.459046000 | -1.598192000 | 1.390801000 |
| H | -4.891289000 | 0.565022000 | -1.577040000 |
| C | 0.997738000 | 1.514623000 | 1.149192000 |
| B | -3.969680000 | -0.035654000 | 0.744931000 |
| H | -0.999948000 | -2.519402000 | 1.220767000 |
| C | 0.421541000 | 1.072709000 | -1.236161000 |
| B | -4.656721000 | -1.522317000 | 0.077322000 |
| C | 1.424910000 | 3.206455000 | -0.665383000 |
| H | 1.849738000 | 3.871719000 | 0.081199000 |
| C | 1.354799000 | 3.601544000 | -1.998708000 |
| H | 1.715560000 | 4.582112000 | -2.291634000 |
| C | 0.835073000 | 2.733701000 | -2.954945000 |
| H | 0.789007000 | 3.034438000 | -3.996533000 |
| C | 0.371595000 | 1.475744000 | -2.573071000 |
| H | -0.039364000 | 0.808851000 | -3.326477000 |
| H | 1.658396000 | -1.100496000 | 3.209325000 |
| C | 1.321097000 | -0.116476000 | 2.894860000 |
| C | -2.455931000 | 1.908768000 | -0.499885000 |

| | | | |
|---|---|---|---|
| H | -1.719304000 | 2.233307000 | 0.236946000 |
| H | -2.084546000 | 2.140481000 | -1.498011000 |
| H | -3.384686000 | 2.455376000 | -0.329610000 |
| C | 1.102520000 | -1.371781000 | -0.452434000 |
| H | 1.636205000 | -1.540139000 | -1.397036000 |
| H | 0.735073000 | -2.341595000 | -0.112081000 |
| C | 2.098940000 | -0.769327000 | 0.595238000 |
| H | 2.412462000 | -1.603784000 | 1.230857000 |
| C | 3.384775000 | -0.187656000 | -0.024834000 |
| H | 4.003982000 | 0.177026000 | 0.805087000 |
| H | 3.148355000 | 0.684316000 | -0.642318000 |
| C | 4.204527000 | -1.180313000 | -0.854761000 |
| H | 5.147239000 | -0.691562000 | -1.130797000 |
| H | 3.698369000 | -1.397408000 | -1.804228000 |
| C | 4.517406000 | -2.497514000 | -0.146040000 |
| H | 3.589472000 | -3.060535000 | 0.013928000 |
| H | 4.927201000 | -2.285930000 | 0.850544000 |
| C | 5.497720000 | -3.359169000 | -0.937496000 |
| H | 6.451807000 | -2.840538000 | -1.072500000 |
| H | 5.698459000 | -4.307797000 | -0.433665000 |
| H | 5.100451000 | -3.585173000 | -1.932028000 |

65

E (2'-tBu_1,2-insert-ts) = -1093.22066746 a.u.

| | | | |
|---|---|---|---|
| H | -1.567356000 | -2.093561000 | 1.215695000 |
| B | -2.517438000 | 1.298250000 | -0.297486000 |
| C | 1.598459000 | 1.562209000 | 0.693744000 |
| H | -1.951861000 | 2.329595000 | -0.239610000 |
| H | -2.067188000 | 0.618303000 | -2.705716000 |
| B | 0.040711000 | 0.079443000 | -0.589900000 |
| C | -1.587821000 | -0.151767000 | -0.387851000 |
| B | -2.436394000 | -1.411097000 | -1.164100000 |
| H | -4.677721000 | 1.780376000 | -1.583831000 |

| | | | |
|---|---|---|---|
| C | 0.999056000 | -0.769305000 | 0.630641000 |
| B | -3.894798000 | 0.931647000 | 0.733471000 |
| H | -6.015964000 | -0.402767000 | 0.109287000 |
| H | -4.616215000 | -1.132686000 | -2.528866000 |
| C | -2.362803000 | 0.238564000 | 1.023313000 |
| B | -2.268474000 | -1.396119000 | 0.580626000 |
| C | 1.427490000 | -2.260147000 | 2.514432000 |
| H | 1.316436000 | -3.236868000 | 2.972896000 |
| H | -4.238680000 | -2.897302000 | -0.054177000 |
| B | -4.078265000 | 0.926792000 | -1.022935000 |
| H | -3.988427000 | -1.018513000 | 2.399988000 |
| B | -4.036448000 | -0.762009000 | -1.564594000 |
| C | 2.136168000 | -1.252526000 | 3.171268000 |
| H | 2.572122000 | -1.443256000 | 4.146747000 |
| B | -2.596027000 | 0.255237000 | -1.710280000 |
| C | 2.270260000 | -0.000128000 | 2.583898000 |
| H | 2.785591000 | 0.797118000 | 3.111323000 |
| B | -3.823722000 | -1.790912000 | -0.135464000 |
| H | -4.250730000 | 1.770310000 | 1.488625000 |
| C | 1.692251000 | 0.250684000 | 1.337882000 |
| B | -3.738997000 | -0.732942000 | 1.278831000 |
| H | -1.804551000 | -2.203384000 | -1.781123000 |
| C | 0.682513000 | 1.529089000 | -0.366788000 |
| B | -4.843102000 | -0.341165000 | -0.044682000 |
| C | 2.311713000 | 2.721408000 | 1.013347000 |
| H | 3.030416000 | 2.729103000 | 1.828170000 |
| C | 2.108190000 | 3.863077000 | 0.250082000 |
| H | 2.653008000 | 4.772812000 | 0.480996000 |
| C | 1.213342000 | 3.842840000 | -0.825644000 |
| H | 1.066096000 | 4.740594000 | -1.417686000 |
| C | 0.516249000 | 2.680729000 | -1.140728000 |
| H | -0.171280000 | 2.673672000 | -1.983851000 |

| | | | |
|---|---|---|---|
| H | 0.379343000 | -2.839681000 | 0.752878000 |
| C | 0.883162000 | -2.021038000 | 1.258499000 |
| C | -1.531327000 | 0.700819000 | 2.207616000 |
| H | -0.931248000 | -0.119497000 | 2.608049000 |
| H | -0.869534000 | 1.519545000 | 1.918783000 |
| H | -2.208172000 | 1.055552000 | 2.986361000 |
| C | 0.512580000 | -0.424104000 | -2.105249000 |
| H | 1.008102000 | 0.353194000 | -2.678819000 |
| H | -0.282971000 | -0.927925000 | -2.644037000 |
| C | 1.342465000 | -1.276251000 | -1.295997000 |
| H | 0.916450000 | -2.265556000 | -1.137797000 |
| C | 2.868273000 | -1.316580000 | -1.438633000 |
| C | 3.536296000 | -2.200173000 | -0.382948000 |
| H | 4.581948000 | -2.364537000 | -0.659877000 |
| H | 3.516238000 | -1.734073000 | 0.605243000 |
| H | 3.048621000 | -3.178587000 | -0.313805000 |
| C | 3.040358000 | -1.987010000 | -2.822719000 |
| H | 2.549272000 | -2.964749000 | -2.860952000 |
| H | 2.630370000 | -1.362553000 | -3.620282000 |
| H | 4.106707000 | -2.137130000 | -3.013668000 |
| C | 3.548371000 | 0.054393000 | -1.466603000 |
| H | 3.036940000 | 0.767694000 | -2.119132000 |
| H | 3.608137000 | 0.490146000 | -0.468413000 |
| H | 4.569408000 | -0.069709000 | -1.840201000 |

65

E (2’-tBu_1,2-insert-pdt) = -1093.26445576 a.u.

| | | | |
|---|---|---|---|
| H | 1.437191000 | -2.382151000 | 0.220641000 |
| B | 2.852114000 | 1.127584000 | -0.400760000 |
| C | -1.472010000 | 1.370163000 | -0.776649000 |
| H | 2.304626000 | 2.040236000 | -0.908556000 |
| H | 2.738876000 | 1.807966000 | 2.057946000 |
| B | 0.341246000 | 0.322339000 | 0.725033000 |

| | | | |
|---|---|---|---|
| C | 1.905050000 | 0.034280000 | 0.528753000 |
| B | 2.787846000 | -0.748023000 | 1.763058000 |
| H | 5.213369000 | 1.912459000 | 0.195703000 |
| C | -1.922126000 | -1.135729000 | -0.438172000 |
| B | 4.021608000 | 0.114598000 | -1.237895000 |
| H | 6.145264000 | -0.970083000 | -0.252607000 |
| H | 5.158821000 | -0.080845000 | 2.516761000 |
| C | 2.438299000 | -0.431696000 | -0.951772000 |
| B | 2.323797000 | -1.602567000 | 0.299559000 |
| C | -2.302560000 | -2.500486000 | -2.438044000 |
| H | -2.469353000 | -3.479500000 | -2.874963000 |
| H | 4.318576000 | -2.800177000 | 1.380340000 |
| B | 4.484077000 | 0.980848000 | 0.231899000 |
| H | 3.744580000 | -2.408008000 | -1.645089000 |
| B | 4.447906000 | -0.184566000 | 1.575743000 |
| C | -2.242609000 | -1.366454000 | -3.240381000 |
| H | -2.359573000 | -1.441307000 | -4.316387000 |
| B | 3.097137000 | 0.939402000 | 1.336758000 |
| C | -2.007728000 | -0.132883000 | -2.648783000 |
| H | -1.915531000 | 0.752111000 | -3.271837000 |
| B | 3.963434000 | -1.766894000 | 0.925201000 |
| H | 4.295313000 | 0.396137000 | -2.353735000 |
| C | -1.834459000 | 0.008348000 | -1.263426000 |
| B | 3.690959000 | -1.569516000 | -0.812308000 |
| H | 2.217664000 | -1.003598000 | 2.767028000 |
| C | -0.364843000 | 1.550361000 | 0.072187000 |
| B | 5.016253000 | -0.697396000 | -0.022468000 |
| C | -2.164830000 | 2.488400000 | -1.251562000 |
| H | -3.018013000 | 2.343159000 | -1.908420000 |
| C | -1.796922000 | 3.771875000 | -0.863570000 |
| H | -2.360884000 | 4.626733000 | -1.222546000 |
| C | -0.704971000 | 3.957651000 | -0.019747000 |

| | | | |
|---|---|---|---|
| H | -0.407448000 | 4.957015000 | 0.279789000 |
| C | 0.012826000 | 2.853183000 | 0.427611000 |
| H | 0.878113000 | 3.004945000 | 1.068163000 |
| H | -2.211824000 | -3.258881000 | -0.440236000 |
| C | -2.147560000 | -2.370712000 | -1.063200000 |
| C | 1.430343000 | -0.528784000 | -2.085126000 |
| H | 0.660773000 | -1.274899000 | -1.869123000 |
| H | 0.948881000 | 0.435756000 | -2.258831000 |
| H | 1.957806000 | -0.826447000 | -2.992546000 |
| C | -0.511372000 | -0.572590000 | 1.681273000 |
| H | -0.754197000 | 0.112121000 | 2.509894000 |
| H | 0.071815000 | -1.382056000 | 2.125542000 |
| C | -1.834921000 | -1.153939000 | 1.088272000 |
| H | -1.796434000 | -2.222133000 | 1.331521000 |
| C | -3.137246000 | -0.673993000 | 1.811194000 |
| C | -4.343887000 | -1.408277000 | 1.211036000 |
| H | -5.252632000 | -1.147021000 | 1.762339000 |
| H | -4.495411000 | -1.142788000 | 0.161180000 |
| H | -4.215894000 | -2.495317000 | 1.270533000 |
| C | -3.037987000 | -1.063306000 | 3.294043000 |
| H | -2.845866000 | -2.135835000 | 3.407757000 |
| H | -2.245720000 | -0.520526000 | 3.816874000 |
| H | -3.979773000 | -0.834830000 | 3.802415000 |
| C | -3.381561000 | 0.834052000 | 1.704785000 |
| H | -2.515386000 | 1.422094000 | 2.027269000 |
| H | -3.624132000 | 1.128309000 | 0.681208000 |
| H | -4.229026000 | 1.111529000 | 2.340551000 |

63

E (2’-Ph_2,1-insert-ts) = -1166.99178329 a.u.

| | | | |
|---|---|---|---|
| H | 2.931523000 | 0.901857000 | -1.468617000 |
| B | 1.696461000 | -1.261982000 | 1.446245000 |
| C | -1.299403000 | 1.762524000 | 0.934914000 |

| | | | |
|---|---|---|---|
| H | 0.895166000 | -1.018485000 | 2.273101000 |
| H | 0.422028000 | -2.909865000 | -0.009237000 |
| B | -0.009070000 | 0.072420000 | -0.382094000 |
| C | 1.452702000 | -0.651781000 | -0.150180000 |
| B | 2.219462000 | -1.617235000 | -1.329634000 |
| H | 2.605780000 | -3.578213000 | 2.044446000 |
| C | 0.147134000 | 1.762651000 | -0.993589000 |
| B | 3.422330000 | -1.138308000 | 1.758574000 |
| H | 5.296568000 | -2.676383000 | 0.871514000 |
| H | 3.182062000 | -3.983375000 | -0.937518000 |
| C | 2.604857000 | 0.034819000 | 0.826419000 |
| B | 2.922161000 | -0.088259000 | -0.836778000 |
| C | 1.219347000 | 3.794544000 | -1.798617000 |
| H | 1.906907000 | 4.273547000 | -2.487282000 |
| H | 4.733893000 | -1.531918000 | -1.917818000 |
| B | 2.719218000 | -2.684605000 | 1.275935000 |
| H | 5.025243000 | 0.409224000 | 0.482184000 |
| B | 3.051486000 | -2.911103000 | -0.451606000 |
| C | 0.524282000 | 4.554183000 | -0.855922000 |
| H | 0.676930000 | 5.627176000 | -0.802823000 |
| B | 1.460956000 | -2.347435000 | 0.082588000 |
| C | -0.356626000 | 3.932820000 | 0.018300000 |
| H | -0.876488000 | 4.513279000 | 0.774476000 |
| B | 3.955944000 | -1.496538000 | -1.025677000 |
| H | 3.756832000 | -0.812942000 | 2.845761000 |
| C | -0.543124000 | 2.550033000 | -0.037109000 |
| B | 4.179207000 | -0.407545000 | 0.349946000 |
| H | 1.719060000 | -1.681631000 | -2.403161000 |
| C | -0.976146000 | 0.402626000 | 0.840184000 |
| B | 4.269628000 | -2.157675000 | 0.590504000 |
| C | -2.224314000 | 2.233454000 | 1.873129000 |
| H | -2.482151000 | 3.287552000 | 1.921573000 |

| | | | |
|---|---|---|---|
| C | -2.824305000 | 1.325165000 | 2.733137000 |
| H | -3.545074000 | 1.670360000 | 3.467439000 |
| C | -2.510970000 | -0.036745000 | 2.653441000 |
| H | -2.988751000 | -0.738127000 | 3.329770000 |
| C | -1.607064000 | -0.497736000 | 1.704342000 |
| H | -1.401138000 | -1.562289000 | 1.626251000 |
| H | 1.530531000 | 1.870683000 | -2.655433000 |
| C | 1.017615000 | 2.422673000 | -1.874904000 |
| C | 2.340242000 | 1.414148000 | 1.405479000 |
| H | 2.422430000 | 2.185212000 | 0.636587000 |
| H | 1.344653000 | 1.459405000 | 1.851631000 |
| H | 3.080760000 | 1.608411000 | 2.182786000 |
| C | -0.698203000 | 0.413716000 | -2.125443000 |
| H | -1.540971000 | 1.073028000 | -2.311759000 |
| H | 0.077207000 | 0.435905000 | -2.882633000 |
| C | -0.938561000 | -0.850864000 | -1.495701000 |
| H | -0.307270000 | -1.652996000 | -1.862651000 |
| C | -2.317194000 | -1.303682000 | -1.157045000 |
| C | -2.555304000 | -2.677493000 | -1.059247000 |
| C | -3.383676000 | -0.425191000 | -0.936458000 |
| C | -3.821351000 | -3.163301000 | -0.744525000 |
| H | -1.736574000 | -3.371196000 | -1.231419000 |
| C | -4.648882000 | -0.908935000 | -0.626114000 |
| H | -3.223446000 | 0.649662000 | -0.966369000 |
| C | -4.874322000 | -2.280432000 | -0.527293000 |
| H | -3.982560000 | -4.234051000 | -0.671743000 |
| H | -5.460394000 | -0.209890000 | -0.451080000 |
| H | -5.862067000 | -2.655792000 | -0.281201000 |

63

E (2'-Ph_2,1-insert-pdt) = -1167.05046851 a.u.

| | | | |
|---|---|---|---|
| H | 0.218364000 | -1.982323000 | -1.772452000 |
| B | -1.795937000 | -1.521838000 | 1.449921000 |

| | | | |
|---|---|---|---|
| C | 2.368628000 | 0.276444000 | 1.024284000 |
| H | -1.420112000 | -1.121079000 | 2.491835000 |
| H | -3.093216000 | 0.498148000 | 0.577989000 |
| B | -0.022281000 | 0.298409000 | 0.084206000 |
| C | -1.106515000 | -0.878625000 | 0.012669000 |
| B | -2.165734000 | -0.969762000 | -1.328120000 |
| H | -4.265426000 | -2.105832000 | 1.776782000 |
| C | 2.467131000 | 0.806537000 | -1.442273000 |
| B | -2.029573000 | -3.238680000 | 1.133174000 |
| H | -3.874132000 | -4.337028000 | -0.292428000 |
| H | -4.674396000 | -1.517220000 | -1.201129000 |
| C | -0.689985000 | -2.407772000 | 0.509215000 |
| B | -0.797829000 | -2.068187000 | -1.169789000 |
| C | 3.847571000 | -0.540391000 | -2.920112000 |
| H | 4.217790000 | -0.748889000 | -3.918487000 |
| H | -2.548601000 | -3.083765000 | -2.754896000 |
| B | -3.384407000 | -2.112673000 | 0.986229000 |
| H | -0.783186000 | -4.563291000 | -0.680419000 |
| B | -3.614004000 | -1.776237000 | -0.742545000 |
| C | 4.253409000 | -1.322620000 | -1.843796000 |
| H | 4.941521000 | -2.147914000 | -1.993444000 |
| B | -2.766409000 | -0.602440000 | 0.288700000 |
| C | 3.767810000 | -1.044154000 | -0.570255000 |
| H | 4.072988000 | -1.656422000 | 0.274550000 |
| B | -2.385716000 | -2.684531000 | -1.652503000 |
| H | -1.817978000 | -3.992619000 | 2.019937000 |
| C | 2.870267000 | 0.007254000 | -0.353581000 |
| B | -1.408938000 | -3.578844000 | -0.483960000 |
| H | -2.101754000 | -0.126925000 | -2.150209000 |
| C | 0.986675000 | 0.425782000 | 1.278219000 |
| B | -3.151308000 | -3.401880000 | -0.220301000 |
| C | 3.275700000 | 0.332471000 | 2.086278000 |

| | | | |
|---|---|---|---|
| H | 4.334960000 | 0.207866000 | 1.880671000 |
| C | 2.840712000 | 0.577034000 | 3.384845000 |
| H | 3.560526000 | 0.630880000 | 4.195171000 |
| C | 1.485749000 | 0.771794000 | 3.637056000 |
| H | 1.140577000 | 0.983681000 | 4.643596000 |
| C | 0.570527000 | 0.684065000 | 2.591905000 |
| H | -0.485325000 | 0.833688000 | 2.798068000 |
| H | 2.653105000 | 1.133167000 | -3.550555000 |
| C | 2.963390000 | 0.514708000 | -2.712517000 |
| C | 0.691232000 | -2.679799000 | 1.082676000 |
| H | 1.473975000 | -2.357882000 | 0.392704000 |
| H | 0.829305000 | -2.175935000 | 2.040209000 |
| H | 0.782405000 | -3.756856000 | 1.230892000 |
| C | 1.479724000 | 1.929797000 | -1.241435000 |
| H | 1.749536000 | 2.505204000 | -0.349348000 |
| H | 1.509817000 | 2.617235000 | -2.092608000 |
| C | 0.053416000 | 1.365725000 | -1.081436000 |
| H | -0.245212000 | 0.914490000 | -2.033243000 |
| C | -0.975075000 | 2.428201000 | -0.713313000 |
| C | -2.081587000 | 2.672388000 | -1.533923000 |
| C | -0.847596000 | 3.167967000 | 0.469906000 |
| C | -3.037060000 | 3.619106000 | -1.180027000 |
| H | -2.191174000 | 2.116276000 | -2.459835000 |
| C | -1.804666000 | 4.114824000 | 0.825427000 |
| H | 0.009287000 | 3.006194000 | 1.118124000 |
| C | -2.905316000 | 4.340058000 | 0.004578000 |
| H | -3.886628000 | 3.793999000 | -1.832216000 |
| H | -1.687869000 | 4.676492000 | 1.746757000 |
| H | -3.653311000 | 5.075209000 | 0.282575000 |

65

E (2’-nBu_2,1-insert-ts) = -1093.20951957 a.u.

| | | | |
|---|---|---|---|
| H | -2.228212000 | 0.297074000 | -2.102299000 |

| | | | |
|---|---|---|---|
| B | -1.663418000 | 1.050427000 | 1.613829000 |
| C | 0.690425000 | -2.450790000 | 0.790518000 |
| H | -1.260000000 | 0.348954000 | 2.468244000 |
| H | 0.327044000 | 2.593308000 | 1.239209000 |
| B | 0.279784000 | -0.136218000 | -0.079811000 |
| C | -1.012472000 | 0.884782000 | 0.024500000 |
| B | -1.129712000 | 2.334329000 | -0.869324000 |
| H | -2.254540000 | 3.254195000 | 2.773567000 |
| C | 0.013361000 | -1.451082000 | -1.295619000 |
| B | -3.381192000 | 1.363177000 | 1.411254000 |
| H | -4.505373000 | 3.578814000 | 0.710123000 |
| H | -1.690806000 | 4.659813000 | 0.109574000 |
| C | -2.538378000 | 0.322680000 | 0.352068000 |
| B | -2.240401000 | 1.011439000 | -1.169990000 |
| C | -1.089944000 | -2.741544000 | -3.040843000 |
| H | -1.582735000 | -2.769095000 | -4.006729000 |
| H | -3.259435000 | 3.178833000 | -2.063774000 |
| B | -2.270015000 | 2.698989000 | 1.727727000 |
| H | -4.721007000 | 0.804933000 | -0.706297000 |
| B | -1.943435000 | 3.504854000 | 0.182034000 |
| C | -0.912058000 | -3.916350000 | -2.307999000 |
| H | -1.274854000 | -4.861468000 | -2.698632000 |
| B | -0.770554000 | 2.362558000 | 0.855140000 |
| C | -0.276917000 | -3.871087000 | -1.075101000 |
| H | -0.162907000 | -4.776343000 | -0.486476000 |
| B | -2.854441000 | 2.655396000 | -1.081659000 |
| H | -4.122075000 | 0.852049000 | 2.179219000 |
| C | 0.175944000 | -2.653325000 | -0.562442000 |
| B | -3.739383000 | 1.333493000 | -0.309439000 |
| H | -0.291929000 | 2.556171000 | -1.679015000 |
| C | 0.681202000 | -1.091629000 | 1.134954000 |
| B | -3.565280000 | 2.881842000 | 0.527724000 |

| | | | |
|---|---|---|---|
| C | 1.107547000 | -3.434445000 | 1.693793000 |
| H | 1.126400000 | -4.481846000 | 1.406691000 |
| C | 1.509845000 | -3.050783000 | 2.964657000 |
| H | 1.834534000 | -3.800008000 | 3.679443000 |
| C | 1.498154000 | -1.699794000 | 3.329441000 |
| H | 1.803755000 | -1.410992000 | 4.330019000 |
| C | 1.100980000 | -0.727205000 | 2.419107000 |
| H | 1.102740000 | 0.318872000 | 2.714387000 |
| H | -0.720943000 | -0.648391000 | -3.166657000 |
| C | -0.616721000 | -1.532801000 | -2.547010000 |
| C | -2.758789000 | -1.178551000 | 0.427343000 |
| H | -2.728921000 | -1.629457000 | -0.566779000 |
| H | -1.998890000 | -1.648316000 | 1.055213000 |
| H | -3.739235000 | -1.360827000 | 0.869937000 |
| C | 1.448012000 | -0.150065000 | -1.592288000 |
| H | 2.157758000 | -0.958018000 | -1.754318000 |
| H | 1.001788000 | 0.258775000 | -2.491812000 |
| C | 1.671826000 | 0.739150000 | -0.491030000 |
| H | 1.379208000 | 1.765131000 | -0.687443000 |
| C | 2.961098000 | 0.601151000 | 0.298701000 |
| C | 4.155825000 | 1.232881000 | -0.425290000 |
| H | 5.065072000 | 1.023385000 | 0.152020000 |
| H | 4.293488000 | 0.745450000 | -1.401309000 |
| H | 2.844848000 | 1.074898000 | 1.280584000 |
| H | 3.164440000 | -0.457501000 | 0.491703000 |
| C | 4.038834000 | 2.743403000 | -0.628513000 |
| H | 3.185688000 | 2.969411000 | -1.278755000 |
| H | 3.823564000 | 3.219527000 | 0.336580000 |
| C | 5.305420000 | 3.344663000 | -1.232070000 |
| H | 5.206989000 | 4.422727000 | -1.380220000 |
| H | 6.168097000 | 3.171571000 | -0.581345000 |
| H | 5.527525000 | 2.891393000 | -2.203350000 |

65

E (2'-nBu_2,1-insert-pdt) = -1093.27013147 a.u.

| | | | |
|---|---|---|---|
| H | 0.884441000 | -0.271392000 | -2.250541000 |
| B | 2.640671000 | 1.037882000 | 0.891737000 |
| C | -1.776191000 | 1.752479000 | 0.786635000 |
| H | 2.217928000 | 1.786908000 | 1.696779000 |
| H | 2.468461000 | -1.178482000 | 2.142670000 |
| B | 0.007606000 | -0.098992000 | 0.561651000 |
| C | 1.551432000 | -0.054441000 | 0.128595000 |
| B | 2.311098000 | -1.487919000 | -0.414121000 |
| H | 5.029112000 | 0.478488000 | 1.617242000 |
| C | -2.517890000 | 0.080280000 | -0.951242000 |
| B | 3.756069000 | 1.539732000 | -0.372745000 |
| H | 5.722956000 | 0.193464000 | -1.355436000 |
| H | 4.691460000 | -2.226787000 | 0.226501000 |
| C | 2.118621000 | 1.247733000 | -0.712385000 |
| B | 1.832774000 | -0.227182000 | -1.543874000 |
| C | -3.119818000 | 0.833457000 | -3.182258000 |
| H | -3.446419000 | 0.598952000 | -4.190057000 |
| H | 3.657826000 | -1.687585000 | -2.608466000 |
| B | 4.220141000 | 0.278700000 | 0.776567000 |
| H | 3.270893000 | 1.377430000 | -2.889972000 |
| B | 4.019377000 | -1.288744000 | -0.038329000 |
| C | -2.884388000 | 2.153604000 | -2.810155000 |
| H | -3.024257000 | 2.958413000 | -3.524196000 |
| B | 2.783632000 | -0.711337000 | 1.099970000 |
| C | -2.460152000 | 2.437506000 | -1.516340000 |
| H | -2.255255000 | 3.463836000 | -1.222861000 |
| B | 3.422445000 | -0.984940000 | -1.685525000 |
| H | 4.106826000 | 2.669427000 | -0.377518000 |
| C | -2.260162000 | 1.415436000 | -0.581519000 |
| B | 3.253759000 | 0.764996000 | -1.878138000 |

| | | | |
|---|---|---|---|
| H | 1.680951000 | -2.488186000 | -0.400573000 |
| C | -0.673251000 | 1.070416000 | 1.347853000 |
| B | 4.613364000 | 0.107422000 | -0.951247000 |
| C | -2.384699000 | 2.786601000 | 1.502344000 |
| H | -3.226388000 | 3.310679000 | 1.058681000 |
| C | -1.946952000 | 3.123207000 | 2.779842000 |
| H | -2.442413000 | 3.917553000 | 3.328727000 |
| C | -0.884299000 | 2.433018000 | 3.354739000 |
| H | -0.545912000 | 2.682440000 | 4.354903000 |
| C | -0.246281000 | 1.426852000 | 2.634306000 |
| H | 0.594563000 | 0.904609000 | 3.083758000 |
| H | -3.141062000 | -1.217671000 | -2.537107000 |
| C | -2.940587000 | -0.187636000 | -2.253798000 |
| C | 1.178265000 | 2.404381000 | -1.005154000 |
| H | 0.283725000 | 2.061846000 | -1.530402000 |
| H | 0.878586000 | 2.907415000 | -0.084156000 |
| H | 1.702999000 | 3.116046000 | -1.644245000 |
| C | -2.328293000 | -1.040596000 | 0.041156000 |
| H | -2.763406000 | -0.752583000 | 1.006811000 |
| H | -2.873359000 | -1.925333000 | -0.304731000 |
| C | -0.841230000 | -1.383222000 | 0.246906000 |
| H | -0.463117000 | -1.880933000 | -0.654630000 |
| C | -0.643377000 | -2.333867000 | 1.458340000 |
| C | -1.454325000 | -3.631945000 | 1.373354000 |
| H | -1.230278000 | -4.233650000 | 2.262968000 |
| H | -2.528098000 | -3.410288000 | 1.422017000 |
| H | 0.419101000 | -2.589904000 | 1.550807000 |
| H | -0.920242000 | -1.799637000 | 2.377977000 |
| C | -1.161312000 | -4.461705000 | 0.125058000 |
| H | -1.462353000 | -3.904462000 | -0.770785000 |
| H | -0.077803000 | -4.615318000 | 0.039911000 |
| C | -1.878113000 | -5.809314000 | 0.146527000 |

| | | | |
|---|---|---|---|
| H | -1.671716000 | -6.388750000 | -0.756580000 |
| H | -1.562545000 | -6.405474000 | 1.008103000 |
| H | -2.961866000 | -5.672641000 | 0.217847000 |

65

E (2'-tBu_2,1-insert-ts) = -1093.20762181 a.u.

| | | | |
|---|---|---|---|
| H | 2.047600000 | 1.595031000 | -1.688428000 |
| B | 1.990469000 | -0.508194000 | 1.514086000 |
| C | -2.086707000 | 0.837109000 | 1.033930000 |
| H | 1.214879000 | -0.505934000 | 2.398542000 |
| H | 1.430508000 | -2.695976000 | 0.334143000 |
| B | -0.218424000 | -0.208085000 | -0.262857000 |
| C | 1.423484000 | -0.247989000 | -0.095819000 |
| B | 2.460411000 | -0.952486000 | -1.256998000 |
| H | 3.808420000 | -2.167518000 | 2.217817000 |
| C | -0.817712000 | 1.384044000 | -0.950002000 |
| B | 3.524403000 | 0.339477000 | 1.641103000 |
| H | 5.814004000 | -0.397616000 | 0.708271000 |
| H | 4.335302000 | -2.656715000 | -0.761605000 |
| C | 2.242195000 | 0.958837000 | 0.699595000 |
| B | 2.486961000 | 0.775546000 | -0.970418000 |
| C | -0.829382000 | 3.641246000 | -1.873434000 |
| H | -0.443031000 | 4.349201000 | -2.598802000 |
| H | 4.673038000 | 0.077603000 | -2.098997000 |
| B | 3.498297000 | -1.404899000 | 1.366689000 |
| H | 4.264254000 | 2.241984000 | 0.083311000 |
| B | 3.798613000 | -1.680884000 | -0.358375000 |
| C | -1.807958000 | 4.033502000 | -0.958510000 |
| H | -2.181719000 | 5.052114000 | -0.961561000 |
| B | 2.147127000 | -1.753067000 | 0.280087000 |
| C | -2.292051000 | 3.116108000 | -0.039456000 |
| H | -3.027824000 | 3.420575000 | 0.698618000 |
| B | 4.002148000 | -0.099560000 | -1.139329000 |

| | | | |
|---|---|---|---|
| H | 3.752656000 | 0.899471000 | 2.657946000 |
| C | -1.799972000 | 1.807431000 | -0.019431000 |
| B | 3.828171000 | 1.142089000 | 0.107246000 |
| H | 1.982985000 | -1.343444000 | -2.268880000 |
| C | -1.168867000 | -0.221146000 | 1.014666000 |
| B | 4.650606000 | -0.378508000 | 0.487246000 |
| C | -3.096418000 | 0.913897000 | 1.998892000 |
| H | -3.823255000 | 1.720702000 | 1.981213000 |
| C | -3.164520000 | -0.064504000 | 2.979999000 |
| H | -3.941325000 | -0.020318000 | 3.736462000 |
| C | -2.223698000 | -1.100566000 | 3.007286000 |
| H | -2.264834000 | -1.844312000 | 3.796597000 |
| C | -1.242186000 | -1.185356000 | 2.026505000 |
| H | -0.521529000 | -1.997186000 | 2.057663000 |
| H | 0.346098000 | 2.053728000 | -2.650645000 |
| C | -0.360829000 | 2.334612000 | -1.878413000 |
| C | 1.463194000 | 2.166097000 | 1.193152000 |
| H | 1.250742000 | 2.858168000 | 0.376528000 |
| H | 0.521389000 | 1.855335000 | 1.650458000 |
| H | 2.063968000 | 2.677859000 | 1.946484000 |
| C | -0.874131000 | -0.237514000 | -2.041441000 |
| H | -1.862779000 | 0.051069000 | -2.386434000 |
| H | -0.059087000 | 0.051840000 | -2.698019000 |
| C | -0.731258000 | -1.458239000 | -1.307480000 |
| H | 0.162048000 | -2.017621000 | -1.574128000 |
| C | -1.951471000 | -2.399264000 | -1.212206000 |
| C | -1.610542000 | -3.615564000 | -0.345703000 |
| H | -2.425516000 | -4.345584000 | -0.387038000 |
| H | -0.699215000 | -4.106543000 | -0.703294000 |
| H | -1.464039000 | -3.337041000 | 0.698618000 |
| C | -2.192287000 | -2.899478000 | -2.650787000 |
| H | -2.988516000 | -3.650367000 | -2.665188000 |

| | | | |
|---|---|---|---|
| H | -2.494269000 | -2.079849000 | -3.312515000 |
| H | -1.287853000 | -3.355101000 | -3.066707000 |
| C | -3.251203000 | -1.753427000 | -0.711830000 |
| H | -3.457606000 | -0.794691000 | -1.202027000 |
| H | -4.088440000 | -2.419599000 | -0.943970000 |
| H | -3.247042000 | -1.585433000 | 0.366081000 |

65

E (2’-tBu_2,1-insert-pdt) = -1093.26512112 a.u.

| | | | |
|---|---|---|---|
| H | 1.226743000 | 1.065958000 | -2.047259000 |
| B | 2.163104000 | 0.368534000 | 1.598867000 |
| C | -2.168686000 | 0.487556000 | 1.072799000 |
| H | 1.459754000 | 0.408364000 | 2.544916000 |
| H | 2.389057000 | -2.149164000 | 1.282780000 |
| B | -0.060092000 | -0.638615000 | 0.061217000 |
| C | 1.479225000 | -0.150413000 | 0.103062000 |
| B | 2.634074000 | -0.853662000 | -0.945728000 |
| H | 4.496808000 | -0.254325000 | 2.452456000 |
| C | -2.044707000 | 0.786264000 | -1.434446000 |
| B | 3.294303000 | 1.652134000 | 1.182386000 |
| H | 5.641438000 | 1.411789000 | 0.143383000 |
| H | 5.010903000 | -1.567173000 | -0.262897000 |
| C | 1.837385000 | 1.453237000 | 0.334067000 |
| B | 2.026269000 | 0.767658000 | -1.227470000 |
| C | -2.199009000 | 3.018567000 | -2.384652000 |
| H | -2.151164000 | 3.669579000 | -3.251317000 |
| H | 4.276118000 | 0.472003000 | -2.430989000 |
| B | 3.883446000 | 0.016506000 | 1.476941000 |
| H | 3.204209000 | 3.005953000 | -1.000655000 |
| B | 4.177946000 | -0.740631000 | -0.104960000 |
| C | -2.490927000 | 3.541540000 | -1.127846000 |
| H | -2.673106000 | 4.604036000 | -1.004800000 |
| B | 2.699784000 | -1.114478000 | 0.801916000 |

| | | | |
|---|---|---|---|
| C | -2.524872000 | 2.697394000 | -0.022785000 |
| H | -2.701447000 | 3.107155000 | 0.968121000 |
| B | 3.756692000 | 0.436400000 | -1.367958000 |
| H | 3.359769000 | 2.581193000 | 1.911558000 |
| C | -2.276247000 | 1.327254000 | -0.153646000 |
| B | 3.201277000 | 1.906927000 | -0.562736000 |
| H | 2.273482000 | -1.703308000 | -1.682941000 |
| C | -1.049575000 | -0.358812000 | 1.246008000 |
| B | 4.540426000 | 0.976315000 | 0.129686000 |
| C | -3.098590000 | 0.621350000 | 2.107272000 |
| H | -3.955200000 | 1.274215000 | 1.965355000 |
| C | -2.947647000 | -0.082298000 | 3.298142000 |
| H | -3.689047000 | 0.019483000 | 4.083936000 |
| C | -1.841560000 | -0.906713000 | 3.482100000 |
| H | -1.709419000 | -1.445920000 | 4.414229000 |
| C | -0.895884000 | -1.028921000 | 2.467422000 |
| H | -0.024386000 | -1.660538000 | 2.627344000 |
| H | -1.810762000 | 1.231531000 | -3.517255000 |
| C | -1.992778000 | 1.649153000 | -2.530408000 |
| C | 0.715597000 | 2.457199000 | 0.516993000 |
| H | 0.147336000 | 2.579428000 | -0.406953000 |
| H | 0.040873000 | 2.151614000 | 1.318232000 |
| H | 1.160789000 | 3.417547000 | 0.781624000 |
| C | -1.966557000 | -0.712582000 | -1.616931000 |
| H | -2.769776000 | -1.133143000 | -1.007941000 |
| H | -2.196577000 | -0.955018000 | -2.659637000 |
| C | -0.628655000 | -1.388695000 | -1.212952000 |
| H | 0.082497000 | -1.247163000 | -2.037036000 |
| C | -0.808567000 | -2.948441000 | -1.049032000 |
| C | 0.358977000 | -3.547953000 | -0.255858000 |
| H | 0.267971000 | -4.638553000 | -0.219974000 |
| H | 1.327504000 | -3.306424000 | -0.697610000 |

| | | | |
|---|---|---|---|
| H | 0.354680000 | -3.189940000 | 0.781278000 |
| C | -0.828918000 | -3.585279000 | -2.444182000 |
| H | -0.992998000 | -4.665502000 | -2.368155000 |
| H | -1.631207000 | -3.171025000 | -3.063142000 |
| H | 0.122311000 | -3.422427000 | -2.961508000 |
| C | -2.101604000 | -3.333193000 | -0.311644000 |
| H | -2.993625000 | -3.164273000 | -0.920446000 |
| H | -2.071752000 | -4.401980000 | -0.076753000 |
| H | -2.216354000 | -2.786694000 | 0.630346000 |

48

E (1Ph'-Ph_1,2-insert-ts) = -1027.817342952 a.u.

| | | | |
|---|---|---|---|
| C | 0.621393000 | 1.285392000 | 1.014821000 |
| B | -0.912918000 | 0.343905000 | -0.738846000 |
| C | -0.203733000 | -0.884512000 | 0.402157000 |
| C | -0.264678000 | -2.751184000 | 1.945511000 |
| H | -0.575289000 | -3.764808000 | 2.177100000 |
| C | 0.474963000 | -2.015560000 | 2.876090000 |
| H | 0.747108000 | -2.462988000 | 3.826623000 |
| C | 0.826727000 | -0.700776000 | 2.602343000 |
| H | 1.344846000 | -0.106988000 | 3.349913000 |
| C | 0.474073000 | -0.123655000 | 1.380632000 |
| C | -0.167980000 | 1.600866000 | -0.104341000 |
| C | 1.416251000 | 2.249219000 | 1.641800000 |
| H | 2.043313000 | 1.986908000 | 2.489341000 |
| C | 1.409198000 | 3.548749000 | 1.151858000 |
| H | 2.020512000 | 4.309345000 | 1.627385000 |
| C | 0.623244000 | 3.882531000 | 0.043618000 |
| H | 0.621237000 | 4.903911000 | -0.324750000 |
| C | -0.147977000 | 2.911298000 | -0.589191000 |
| H | -0.743172000 | 3.175390000 | -1.460512000 |
| H | -1.173056000 | -2.771624000 | 0.004004000 |
| C | -0.601232000 | -2.187557000 | 0.722273000 |

| | | | |
|---|---|---|---|
| C | -0.322798000 | 0.073125000 | -2.255666000 |
| H | 0.264271000 | 0.890915000 | -2.658695000 |
| H | -1.065950000 | -0.313537000 | -2.946039000 |
| C | 0.385972000 | -0.924017000 | -1.479288000 |
| H | 0.013912000 | -1.937062000 | -1.599325000 |
| C | 1.855354000 | -0.859838000 | -1.234671000 |
| C | 2.587256000 | 0.320460000 | -1.386223000 |
| C | 2.516363000 | -2.028746000 | -0.840133000 |
| C | 3.959365000 | 0.324866000 | -1.149278000 |
| H | 2.089965000 | 1.242701000 | -1.668496000 |
| C | 3.886764000 | -2.025691000 | -0.620419000 |
| H | 1.940660000 | -2.938819000 | -0.690692000 |
| C | 4.612190000 | -0.844555000 | -0.772608000 |
| H | 4.517692000 | 1.248314000 | -1.261890000 |
| H | 4.388800000 | -2.940420000 | -0.323028000 |
| H | 5.682743000 | -0.837270000 | -0.595131000 |
| C | -2.482326000 | 0.076845000 | -0.586881000 |
| C | -3.154805000 | -0.928051000 | -1.293351000 |
| C | -3.232602000 | 0.850290000 | 0.307633000 |
| C | -4.516508000 | -1.157355000 | -1.117918000 |
| H | -2.608156000 | -1.553393000 | -1.998911000 |
| C | -4.597938000 | 0.636019000 | 0.481958000 |
| H | -2.736366000 | 1.635143000 | 0.872719000 |
| C | -5.245202000 | -0.370665000 | -0.229027000 |
| H | -5.010720000 | -1.945113000 | -1.678823000 |
| H | -5.157124000 | 1.255739000 | 1.176678000 |
| H | -6.308632000 | -0.540851000 | -0.093451000 |

48

E (1Ph’-Ph_1,2-insert-pdt) = -1027.87515080 a.u.

| | | | |
|---|---|---|---|
| C | 0.700846000 | -0.281508000 | 1.328252000 |
| B | -1.297172000 | -0.056197000 | -0.366160000 |
| C | 1.409934000 | -1.592477000 | -0.736817000 |

| | | | |
|---|---|---|---|
| C | 2.333485000 | -3.829020000 | -0.467866000 |
| H | 2.756223000 | -4.723242000 | -0.914323000 |
| C | 2.163046000 | -3.740238000 | 0.908613000 |
| H | 2.445192000 | -4.566306000 | 1.553192000 |
| C | 1.621916000 | -2.583160000 | 1.458519000 |
| H | 1.473547000 | -2.513645000 | 2.532320000 |
| C | 1.238285000 | -1.500671000 | 0.658447000 |
| C | -0.500605000 | 0.342121000 | 0.921715000 |
| C | 1.403619000 | 0.221541000 | 2.426824000 |
| H | 2.331838000 | -0.258751000 | 2.722286000 |
| C | 0.954320000 | 1.342665000 | 3.117176000 |
| H | 1.530205000 | 1.729136000 | 3.952269000 |
| C | -0.227411000 | 1.965208000 | 2.730880000 |
| H | -0.587561000 | 2.839840000 | 3.262895000 |
| C | -0.946746000 | 1.453648000 | 1.655533000 |
| H | -1.873774000 | 1.938350000 | 1.359932000 |
| H | 2.094130000 | -2.822005000 | -2.350454000 |
| C | 1.956772000 | -2.758692000 | -1.273926000 |
| C | -0.543927000 | -0.400566000 | -1.715973000 |
| H | -0.864345000 | 0.312291000 | -2.490217000 |
| H | -0.920685000 | -1.370635000 | -2.070832000 |
| C | 0.990601000 | -0.470903000 | -1.671119000 |
| H | 1.336719000 | -0.763209000 | -2.672006000 |
| C | 1.696260000 | 0.847373000 | -1.359969000 |
| C | 1.042073000 | 2.079572000 | -1.372029000 |
| C | 3.069935000 | 0.831440000 | -1.094235000 |
| C | 1.735697000 | 3.261079000 | -1.117047000 |
| H | -0.024526000 | 2.131226000 | -1.567899000 |
| C | 3.766460000 | 2.007435000 | -0.842788000 |
| H | 3.592904000 | -0.121399000 | -1.075255000 |
| C | 3.099753000 | 3.230839000 | -0.850843000 |
| H | 1.202512000 | 4.206605000 | -1.122555000 |

| | | | |
|---|---|---|---|
| H | 4.831595000 | 1.968825000 | -0.636557000 |
| H | 3.639356000 | 4.150572000 | -0.649481000 |
| C | -2.865377000 | -0.058217000 | -0.355710000 |
| C | -3.608109000 | 0.017784000 | -1.547213000 |
| C | -3.585948000 | -0.189786000 | 0.844583000 |
| C | -4.997808000 | -0.014163000 | -1.543798000 |
| H | -3.088303000 | 0.108902000 | -2.497269000 |
| C | -4.976509000 | -0.240489000 | 0.857524000 |
| H | -3.040806000 | -0.264816000 | 1.781380000 |
| C | -5.684417000 | -0.145600000 | -0.337905000 |
| H | -5.547222000 | 0.057950000 | -2.477149000 |
| H | -5.508341000 | -0.352217000 | 1.797117000 |
| H | -6.769681000 | -0.177768000 | -0.331576000 |

50

E (1Ph’-nBu_1,2-insert-ts) = -954.038366617 a.u.

| | | | |
|---|---|---|---|
| C | -0.346208000 | 2.150876000 | 0.226246000 |
| B | -0.466467000 | -0.185981000 | -0.683368000 |
| C | 0.012603000 | -0.078974000 | 1.048159000 |
| C | -0.066654000 | -0.629858000 | 3.408103000 |
| H | -0.071976000 | -1.378530000 | 4.193478000 |
| C | -0.121825000 | 0.727788000 | 3.734462000 |
| H | -0.162185000 | 1.034447000 | 4.774677000 |
| C | -0.163825000 | 1.681594000 | 2.727133000 |
| H | -0.271643000 | 2.733581000 | 2.975511000 |
| C | -0.118230000 | 1.286905000 | 1.387279000 |
| C | -0.591679000 | 1.384310000 | -0.925786000 |
| C | -0.365078000 | 3.548003000 | 0.190410000 |
| H | -0.154664000 | 4.131585000 | 1.082446000 |
| C | -0.652894000 | 4.185302000 | -1.010041000 |
| H | -0.676887000 | 5.269652000 | -1.053948000 |
| C | -0.908019000 | 3.437787000 | -2.164070000 |
| H | -1.138999000 | 3.948474000 | -3.093912000 |

| | | | |
|---|---|---|---|
| C | -0.860691000 | 2.046873000 | -2.125554000 |
| H | -1.047109000 | 1.473083000 | -3.030547000 |
| H | 0.042507000 | -2.085707000 | 1.840831000 |
| C | -0.002326000 | -1.025153000 | 2.078701000 |
| C | 0.788034000 | -0.774086000 | -1.606179000 |
| H | 1.178955000 | -0.040412000 | -2.304723000 |
| H | 0.568098000 | -1.746137000 | -2.034953000 |
| C | 1.327461000 | -0.743639000 | -0.271511000 |
| H | 1.375669000 | -1.708434000 | 0.225369000 |
| C | 2.464488000 | 0.207381000 | 0.045345000 |
| H | 2.639632000 | 0.226469000 | 1.125946000 |
| H | 2.172423000 | 1.217294000 | -0.262102000 |
| C | 3.743862000 | -0.208346000 | -0.692347000 |
| H | 4.530130000 | 0.512485000 | -0.437666000 |
| H | 3.587267000 | -0.129887000 | -1.775001000 |
| C | 4.225410000 | -1.618385000 | -0.352219000 |
| H | 3.482003000 | -2.351646000 | -0.689105000 |
| H | 4.296140000 | -1.724598000 | 0.738025000 |
| C | 5.573236000 | -1.937230000 | -0.993189000 |
| H | 6.343869000 | -1.242470000 | -0.645530000 |
| H | 5.902414000 | -2.951443000 | -0.754504000 |
| H | 5.515535000 | -1.850185000 | -2.082510000 |
| C | -1.725566000 | -1.172050000 | -0.643071000 |
| C | -1.596150000 | -2.566385000 | -0.608730000 |
| C | -3.021684000 | -0.643406000 | -0.609410000 |
| C | -2.707416000 | -3.401691000 | -0.540753000 |
| H | -0.605213000 | -3.020028000 | -0.635238000 |
| C | -4.140782000 | -1.471298000 | -0.552333000 |
| H | -3.153264000 | 0.435365000 | -0.631996000 |
| C | -3.988040000 | -2.854366000 | -0.515573000 |
| H | -2.575272000 | -4.479309000 | -0.513961000 |
| H | -5.134806000 | -1.034228000 | -0.534729000 |

| | | | |
|---|---|---|---|
| H | -4.858453000 | -3.501235000 | -0.469446000 |

50

E (1Ph'-nBu_1,2-insert-pdt) = -954.095005666 a.u.

| | | | |
|---|---|---|---|
| C | 0.923835000 | 1.520985000 | -0.820457000 |
| B | -0.972849000 | -0.006650000 | 0.168122000 |
| C | 2.050522000 | 0.162648000 | 1.036649000 |
| C | 3.516756000 | 1.322700000 | 2.605313000 |
| H | 4.183858000 | 1.281782000 | 3.460361000 |
| C | 3.245651000 | 2.532697000 | 1.977694000 |
| H | 3.691372000 | 3.454326000 | 2.337364000 |
| C | 2.390155000 | 2.555198000 | 0.883086000 |
| H | 2.163308000 | 3.500485000 | 0.398662000 |
| C | 1.785397000 | 1.389961000 | 0.392484000 |
| C | -0.360265000 | 0.936332000 | -0.921756000 |
| C | 1.413742000 | 2.288091000 | -1.883526000 |
| H | 2.404672000 | 2.725061000 | -1.804352000 |
| C | 0.674254000 | 2.465718000 | -3.048954000 |
| H | 1.088390000 | 3.047763000 | -3.866419000 |
| C | -0.586590000 | 1.891434000 | -3.163431000 |
| H | -1.170956000 | 2.021202000 | -4.068570000 |
| C | -1.094314000 | 1.151640000 | -2.099888000 |
| H | -2.086221000 | 0.715335000 | -2.183938000 |
| H | 3.127211000 | -0.786432000 | 2.623685000 |
| C | 2.918230000 | 0.160146000 | 2.132045000 |
| C | -0.100250000 | -1.103977000 | 0.893472000 |
| H | -0.547266000 | -2.084778000 | 0.666334000 |
| H | -0.254909000 | -0.984955000 | 1.976372000 |
| C | 1.412748000 | -1.148703000 | 0.615789000 |
| H | 1.837026000 | -1.913458000 | 1.279267000 |
| C | 1.800900000 | -1.568663000 | -0.817103000 |
| H | 2.891535000 | -1.692475000 | -0.835137000 |

| | | | |
|---|---|---|---|
| H | 1.570197000 | -0.773611000 | -1.530014000 |
| C | 1.143017000 | -2.864713000 | -1.297409000 |
| H | 1.522011000 | -3.093319000 | -2.301451000 |
| H | 0.061343000 | -2.713365000 | -1.416633000 |
| C | 1.388163000 | -4.073027000 | -0.394217000 |
| H | 0.943221000 | -3.898980000 | 0.593451000 |
| H | 2.467717000 | -4.183459000 | -0.227706000 |
| C | 0.819392000 | -5.361175000 | -0.983493000 |
| H | 1.276268000 | -5.581315000 | -1.953280000 |
| H | 0.993667000 | -6.217801000 | -0.327414000 |
| H | -0.260401000 | -5.272705000 | -1.140307000 |
| C | -2.498723000 | 0.099372000 | 0.521516000 |
| C | -3.186167000 | -0.963656000 | 1.133416000 |
| C | -3.221539000 | 1.282639000 | 0.288468000 |
| C | -4.529765000 | -0.863169000 | 1.475430000 |
| H | -2.660285000 | -1.891631000 | 1.341406000 |
| C | -4.561967000 | 1.400147000 | 0.643939000 |
| H | -2.716671000 | 2.128627000 | -0.169632000 |
| C | -5.219879000 | 0.323071000 | 1.231918000 |
| H | -5.039364000 | -1.703849000 | 1.935653000 |
| H | -5.093698000 | 2.328749000 | 0.461850000 |
| H | -6.267593000 | 0.407866000 | 1.503917000 |

50

E (1Ph’-tBu_1,2-insert-ts) = -954.040193711 a.u.

| | | | |
|---|---|---|---|
| C | 1.032626000 | 1.555691000 | 0.689894000 |
| B | -0.538358000 | 0.194333000 | -0.710924000 |
| C | 0.155006000 | -0.684340000 | 0.676449000 |
| C | 0.094838000 | -2.062033000 | 2.676228000 |
| H | -0.246740000 | -2.958579000 | 3.182986000 |
| C | 0.916719000 | -1.150317000 | 3.343565000 |
| H | 1.222367000 | -1.342894000 | 4.366975000 |
| C | 1.301725000 | 0.026039000 | 2.713619000 |

| | | | |
|---|---|---|---|
| H | 1.875850000 | 0.772068000 | 3.255760000 |
| C | 0.900033000 | 0.279226000 | 1.398675000 |
| C | 0.235351000 | 1.565925000 | -0.465686000 |
| C | 1.813608000 | 2.657004000 | 1.048471000 |
| H | 2.441383000 | 2.632351000 | 1.935181000 |
| C | 1.786861000 | 3.789114000 | 0.242875000 |
| H | 2.385716000 | 4.654933000 | 0.507565000 |
| C | 0.995853000 | 3.818202000 | -0.909825000 |
| H | 0.979707000 | 4.711407000 | -1.526847000 |
| C | 0.235453000 | 2.708180000 | -1.268855000 |
| H | -0.369551000 | 2.735306000 | -2.172371000 |
| H | -0.916901000 | -2.549932000 | 0.855188000 |
| C | -0.280381000 | -1.826478000 | 1.360454000 |
| C | -0.027708000 | -0.468545000 | -2.150201000 |
| H | 0.635716000 | 0.178902000 | -2.714658000 |
| H | -0.858812000 | -0.869183000 | -2.721937000 |
| C | 0.571487000 | -1.343713000 | -1.180588000 |
| H | -0.000042000 | -2.252513000 | -0.999874000 |
| C | 2.085475000 | -1.610681000 | -1.156607000 |
| C | 2.507855000 | -2.493176000 | 0.022083000 |
| H | 3.539312000 | -2.826097000 | -0.129886000 |
| H | 2.459473000 | -1.956227000 | 0.972186000 |
| H | 1.874065000 | -3.383398000 | 0.098984000 |
| C | 2.308712000 | -2.405537000 | -2.462724000 |
| H | 1.687876000 | -3.307181000 | -2.493674000 |
| H | 2.077362000 | -1.797834000 | -3.341124000 |
| H | 3.356197000 | -2.715243000 | -2.521332000 |
| C | 2.955123000 | -0.351228000 | -1.192017000 |
| H | 2.625425000 | 0.367400000 | -1.947807000 |
| H | 2.965512000 | 0.154552000 | -0.225804000 |
| H | 3.982901000 | -0.641847000 | -1.431406000 |
| C | -2.119157000 | 0.050352000 | -0.490747000 |

| | | | |
|---|---|---|---|
| C | -2.849243000 | -1.066793000 | -0.915911000 |
| C | -2.819281000 | 1.056506000 | 0.187161000 |
| C | -4.216117000 | -1.181843000 | -0.676933000 |
| H | -2.346672000 | -1.873506000 | -1.449762000 |
| C | -4.188439000 | 0.956048000 | 0.422120000 |
| H | -2.279685000 | 1.934932000 | 0.531960000 |
| C | -4.892765000 | -0.165429000 | -0.007508000 |
| H | -4.754312000 | -2.061082000 | -1.018662000 |
| H | -4.706575000 | 1.755593000 | 0.943403000 |
| H | -5.959571000 | -0.246633000 | 0.175617000 |

50

E (1Ph'-tBu_1,2-insert-pdt) = -954.093099545 a.u.

| | | | |
|---|---|---|---|
| C | 0.764013000 | 1.488184000 | -0.096128000 |
| B | -1.134843000 | -0.320357000 | -0.026315000 |
| C | 1.826379000 | -0.581483000 | 0.972779000 |
| C | 3.193086000 | -0.268363000 | 2.979183000 |
| H | 3.818227000 | -0.700914000 | 3.753657000 |
| C | 2.927392000 | 1.095298000 | 2.962876000 |
| H | 3.334691000 | 1.749314000 | 3.726913000 |
| C | 2.123809000 | 1.615127000 | 1.956789000 |
| H | 1.897725000 | 2.677361000 | 1.945001000 |
| C | 1.568499000 | 0.804942000 | 0.956007000 |
| C | -0.513913000 | 1.037575000 | -0.491005000 |
| C | 1.296549000 | 2.646218000 | -0.674163000 |
| H | 2.284417000 | 2.980491000 | -0.370778000 |
| C | 0.602644000 | 3.344949000 | -1.657551000 |
| H | 1.048744000 | 4.224464000 | -2.111359000 |
| C | -0.657540000 | 2.911274000 | -2.055644000 |
| H | -1.208041000 | 3.449511000 | -2.820455000 |
| C | -1.208594000 | 1.781763000 | -1.458387000 |
| H | -2.197320000 | 1.449428000 | -1.764116000 |
| H | 2.850619000 | -2.147608000 | 2.013657000 |

| | | | |
|---|---|---|---|
| C | 2.642160000 | -1.080961000 | 1.995170000 |
| C | -0.275278000 | -1.642855000 | 0.059452000 |
| H | -0.662389000 | -2.314820000 | -0.724270000 |
| H | -0.553319000 | -2.147146000 | 0.996078000 |
| C | 1.264017000 | -1.592376000 | -0.020691000 |
| H | 1.609071000 | -2.554959000 | 0.378005000 |
| C | 1.878373000 | -1.597558000 | -1.468008000 |
| C | 3.366548000 | -1.239475000 | -1.387246000 |
| H | 3.841472000 | -1.371830000 | -2.364992000 |
| H | 3.506232000 | -0.199732000 | -1.075911000 |
| H | 3.888875000 | -1.877511000 | -0.665438000 |
| C | 1.758840000 | -3.036574000 | -2.000489000 |
| H | 2.298333000 | -3.741193000 | -1.358951000 |
| H | 0.715183000 | -3.362723000 | -2.059329000 |
| H | 2.182009000 | -3.105964000 | -3.007529000 |
| C | 1.203669000 | -0.675413000 | -2.492563000 |
| H | 0.124114000 | -0.849353000 | -2.550381000 |
| H | 1.366852000 | 0.382400000 | -2.281490000 |
| H | 1.621651000 | -0.885003000 | -3.483454000 |
| C | -2.671448000 | -0.407172000 | 0.284427000 |
| C | -3.358669000 | -1.633632000 | 0.264424000 |
| C | -3.406317000 | 0.736364000 | 0.642839000 |
| C | -4.713180000 | -1.715498000 | 0.565558000 |
| H | -2.823380000 | -2.542449000 | 0.002026000 |
| C | -4.758294000 | 0.662556000 | 0.964887000 |
| H | -2.901855000 | 1.698002000 | 0.679580000 |
| C | -5.415052000 | -0.564310000 | 0.919619000 |
| H | -5.222500000 | -2.673408000 | 0.530636000 |
| H | -5.299915000 | 1.559387000 | 1.248717000 |
| H | -6.471423000 | -0.625422000 | 1.162956000 |

38

E (1Br’-Ph_1,2-insert-ts) = -3367.77257197 a.u.

| | | | |
|---|---|---|---|
| C | 0.115353000 | 1.092694000 | 1.187769000 |
| B | -1.296848000 | 0.168991000 | -0.655586000 |
| C | -0.404964000 | -1.065727000 | 0.278784000 |
| C | -0.164326000 | -3.118784000 | 1.548091000 |
| H | -0.325324000 | -4.187494000 | 1.642319000 |
| C | 0.496907000 | -2.417887000 | 2.562856000 |
| H | 0.856928000 | -2.949318000 | 3.438129000 |
| C | 0.655747000 | -1.040695000 | 2.476988000 |
| H | 1.104960000 | -0.489701000 | 3.297915000 |
| C | 0.188618000 | -0.359001000 | 1.353458000 |
| C | -0.758927000 | 1.434390000 | 0.142755000 |
| C | 0.787129000 | 2.071707000 | 1.924401000 |
| H | 1.482329000 | 1.796034000 | 2.712390000 |
| C | 0.569014000 | 3.408755000 | 1.617117000 |
| H | 1.081225000 | 4.183170000 | 2.179283000 |
| C | -0.303105000 | 3.764222000 | 0.582591000 |
| H | -0.471425000 | 4.813578000 | 0.360697000 |
| C | -0.950495000 | 2.782168000 | -0.162989000 |
| H | -1.617840000 | 3.064833000 | -0.973574000 |
| H | -1.121709000 | -2.997413000 | -0.365937000 |
| C | -0.612341000 | -2.446356000 | 0.421575000 |
| C | -0.708311000 | 0.160612000 | -2.175933000 |
| H | -0.275712000 | 1.101292000 | -2.501527000 |
| H | -1.382402000 | -0.276888000 | -2.905554000 |
| C | 0.201433000 | -0.797303000 | -1.552854000 |
| H | 0.010583000 | -1.832285000 | -1.816117000 |
| C | 1.638495000 | -0.508111000 | -1.302600000 |
| C | 2.150766000 | 0.792075000 | -1.311843000 |
| C | 2.498856000 | -1.583241000 | -1.047137000 |
| C | 3.505888000 | 1.007448000 | -1.075247000 |
| H | 1.494644000 | 1.639375000 | -1.482794000 |
| C | 3.851380000 | -1.366840000 | -0.826991000 |

| | | | |
|---|---|---|---|
| H | 2.091589000 | -2.590640000 | -1.007592000 |
| C | 4.357543000 | -0.066907000 | -0.838553000 |
| H | 3.894419000 | 2.020361000 | -1.077488000 |
| H | 4.510672000 | -2.207728000 | -0.639174000 |
| H | 5.413962000 | 0.105870000 | -0.660448000 |
| Br | -3.227160000 | -0.357066000 | -0.563887000 |

38

E (1Br'-Ph_1,2-insert-pdt) = -3367.83359116 a.u.

| | | | |
|---|---|---|---|
| C | 0.446229000 | -0.365562000 | 1.340018000 |
| B | -1.447275000 | -0.632735000 | -0.429242000 |
| C | 1.648362000 | -1.056238000 | -0.814764000 |
| C | 3.406041000 | -2.737682000 | -0.692458000 |
| H | 4.167100000 | -3.328846000 | -1.191187000 |
| C | 3.183367000 | -2.877288000 | 0.672050000 |
| H | 3.761957000 | -3.585438000 | 1.255974000 |
| C | 2.206865000 | -2.103874000 | 1.288928000 |
| H | 2.020964000 | -2.222751000 | 2.352110000 |
| C | 1.430960000 | -1.183813000 | 0.571263000 |
| C | -0.905293000 | -0.187569000 | 0.954453000 |
| C | 0.886666000 | 0.190029000 | 2.546299000 |
| H | 1.927886000 | 0.070457000 | 2.829133000 |
| C | 0.028731000 | 0.910512000 | 3.369379000 |
| H | 0.405172000 | 1.345878000 | 4.289772000 |
| C | -1.305802000 | 1.068446000 | 3.011645000 |
| H | -1.986476000 | 1.620199000 | 3.651311000 |
| C | -1.759117000 | 0.513008000 | 1.822363000 |
| H | -2.801392000 | 0.632168000 | 1.543903000 |
| H | 2.805859000 | -1.723094000 | -2.487131000 |
| C | 2.637367000 | -1.834112000 | -1.419153000 |
| C | -0.620282000 | -0.703515000 | -1.760390000 |
| H | -1.194403000 | -0.223360000 | -2.562085000 |
| H | -0.564428000 | -1.760557000 | -2.058520000 |

| | | | |
|---|---|---|---|
| C | 0.807691000 | -0.139195000 | -1.682436000 |
| H | 1.235769000 | -0.192798000 | -2.692258000 |
| C | 0.887578000 | 1.327255000 | -1.265589000 |
| C | -0.213474000 | 2.183275000 | -1.329364000 |
| C | 2.113567000 | 1.855166000 | -0.848175000 |
| C | -0.096063000 | 3.526850000 | -0.978929000 |
| H | -1.181914000 | 1.809944000 | -1.650371000 |
| C | 2.234925000 | 3.195506000 | -0.499839000 |
| H | 2.978960000 | 1.200536000 | -0.787508000 |
| C | 1.127607000 | 4.038486000 | -0.561954000 |
| H | -0.967735000 | 4.171580000 | -1.029828000 |
| H | 3.195989000 | 3.582073000 | -0.175329000 |
| H | 1.218614000 | 5.083965000 | -0.285972000 |
| Br | -3.333174000 | -1.058125000 | -0.605597000 |

40

E (1Br'-nBu_1,2-insert-ts) = -3293.99309429 a.u.

| | | | |
|---|---|---|---|
| C | -1.474269000 | 1.276009000 | 0.650550000 |
| B | -0.477355000 | -0.456553000 | -0.844728000 |
| C | -0.106579000 | -0.686129000 | 0.871748000 |
| C | 0.091193000 | -1.911843000 | 2.956008000 |
| H | 0.432455000 | -2.787304000 | 3.498144000 |
| C | -0.576459000 | -0.885750000 | 3.631467000 |
| H | -0.744908000 | -0.962907000 | 4.700873000 |
| C | -1.062806000 | 0.210473000 | 2.931520000 |
| H | -1.644271000 | 0.971503000 | 3.443578000 |
| C | -0.852433000 | 0.304933000 | 1.554662000 |
| C | -1.360511000 | 0.855745000 | -0.684918000 |
| C | -2.141565000 | 2.456565000 | 0.986627000 |
| H | -2.209204000 | 2.784503000 | 2.020211000 |
| C | -2.716006000 | 3.213203000 | -0.027402000 |
| H | -3.244142000 | 4.129704000 | 0.215823000 |
| C | -2.615925000 | 2.802953000 | -1.360593000 |

| | | | |
|---|---|---|---|
| H | -3.078955000 | 3.399089000 | -2.140787000 |
| C | -1.924848000 | 1.640151000 | -1.690524000 |
| H | -1.842287000 | 1.330969000 | -2.729581000 |
| H | 0.837471000 | -2.615623000 | 1.075135000 |
| C | 0.318088000 | -1.811621000 | 1.591167000 |
| C | 0.899723000 | -0.105516000 | -1.678187000 |
| H | 0.905757000 | 0.899403000 | -2.091067000 |
| H | 1.184491000 | -0.886583000 | -2.374399000 |
| C | 1.384079000 | -0.243289000 | -0.320853000 |
| H | 1.887176000 | -1.181616000 | -0.110623000 |
| C | 1.933544000 | 0.969726000 | 0.399769000 |
| H | 2.081035000 | 0.735937000 | 1.459162000 |
| H | 1.200502000 | 1.780824000 | 0.333803000 |
| C | 3.256573000 | 1.417165000 | -0.237828000 |
| H | 3.608717000 | 2.301299000 | 0.306885000 |
| H | 3.077531000 | 1.738164000 | -1.271055000 |
| C | 4.347929000 | 0.347312000 | -0.220806000 |
| H | 4.041798000 | -0.501820000 | -0.844530000 |
| H | 4.460522000 | -0.040000000 | 0.800028000 |
| C | 5.685741000 | 0.883134000 | -0.723102000 |
| H | 6.035434000 | 1.708086000 | -0.094969000 |
| H | 6.455635000 | 0.107936000 | -0.719716000 |
| H | 5.593598000 | 1.260796000 | -1.745993000 |
| Br | -1.290412000 | -2.179684000 | -1.465232000 |

40

E (1Br’-nBu_1,2-insert-pdt) = -3294.05381085 a.u.

| | | | |
|---|---|---|---|
| C | -1.487956000 | 0.823711000 | 0.692442000 |
| B | 0.729735000 | 0.832434000 | -0.670805000 |
| C | -1.432948000 | -1.470300000 | -0.479722000 |
| C | -3.503420000 | -2.316356000 | -1.456344000 |
| H | -4.030662000 | -3.106945000 | -1.980331000 |
| C | -4.159813000 | -1.145724000 | -1.096182000 |

| | | | |
|---|---|---|---|
| H | -5.206776000 | -1.000292000 | -1.340900000 |
| C | -3.461000000 | -0.153516000 | -0.420588000 |
| H | -3.968654000 | 0.768342000 | -0.153570000 |
| C | -2.104334000 | -0.291694000 | -0.091252000 |
| C | -0.235988000 | 1.418118000 | 0.392695000 |
| C | -2.241643000 | 1.340064000 | 1.754120000 |
| H | -3.188960000 | 0.871510000 | 2.001064000 |
| C | -1.791625000 | 2.415426000 | 2.512197000 |
| H | -2.393224000 | 2.778665000 | 3.339631000 |
| C | -0.576599000 | 3.019456000 | 2.208181000 |
| H | -0.219750000 | 3.864530000 | 2.787257000 |
| C | 0.178290000 | 2.524948000 | 1.152456000 |
| H | 1.124024000 | 2.997960000 | 0.908113000 |
| H | -1.636600000 | -3.371180000 | -1.440000000 |
| C | -2.155261000 | -2.461254000 | -1.150055000 |
| C | 0.852399000 | -0.672865000 | -1.078251000 |
| H | 1.918400000 | -0.935415000 | -1.094427000 |
| H | 0.540121000 | -0.743421000 | -2.131568000 |
| C | 0.049033000 | -1.692154000 | -0.251727000 |
| H | 0.275715000 | -2.682541000 | -0.665812000 |
| C | 0.426803000 | -1.747095000 | 1.242106000 |
| H | -0.089578000 | -2.610999000 | 1.679556000 |
| H | 0.046345000 | -0.865764000 | 1.765532000 |
| C | 1.929029000 | -1.870685000 | 1.507431000 |
| H | 2.087280000 | -1.920239000 | 2.591905000 |
| H | 2.444953000 | -0.959219000 | 1.173529000 |
| C | 2.586063000 | -3.088887000 | 0.859110000 |
| H | 2.505602000 | -3.022344000 | -0.233036000 |
| H | 2.034227000 | -3.991698000 | 1.151821000 |
| C | 4.055417000 | -3.229135000 | 1.247898000 |
| H | 4.163178000 | -3.337668000 | 2.331519000 |
| H | 4.516908000 | -4.099680000 | 0.775089000 |

| | | | |
|---|---|---|---|
| H | 4.622857000 | -2.343096000 | 0.946327000 |
| Br | 1.978492000 | 2.032428000 | -1.552136000 |

40

E (1Br’-tBu_1,2-insert-ts) = -3293.99614125 a.u.

| | | | |
|---|---|---|---|
| C | -1.039211000 | 1.321745000 | 0.733268000 |
| B | -0.585985000 | -0.807579000 | -0.477867000 |
| C | 0.673514000 | -0.365856000 | 0.663705000 |
| C | 2.206394000 | -0.722374000 | 2.517063000 |
| H | 2.963175000 | -1.330643000 | 3.000776000 |
| C | 1.796387000 | 0.480312000 | 3.099654000 |
| H | 2.243458000 | 0.810037000 | 4.032144000 |
| C | 0.780911000 | 1.228091000 | 2.516582000 |
| H | 0.404034000 | 2.117356000 | 3.013297000 |
| C | 0.194818000 | 0.794857000 | 1.324873000 |
| C | -1.545879000 | 0.434042000 | -0.227769000 |
| C | -1.695092000 | 2.515843000 | 1.039337000 |
| H | -1.286734000 | 3.206630000 | 1.772033000 |
| C | -2.880431000 | 2.816147000 | 0.378921000 |
| H | -3.406961000 | 3.737603000 | 0.606447000 |
| C | -3.398373000 | 1.939888000 | -0.580010000 |
| H | -4.330160000 | 2.183737000 | -1.080841000 |
| C | -2.725864000 | 0.761983000 | -0.895038000 |
| H | -3.129325000 | 0.087722000 | -1.646498000 |
| H | 1.976608000 | -2.074366000 | 0.874754000 |
| C | 1.647980000 | -1.136629000 | 1.316719000 |
| C | 0.005199000 | -0.761896000 | -2.013430000 |
| H | -0.413141000 | 0.036285000 | -2.619414000 |
| H | -0.008574000 | -1.736410000 | -2.489707000 |
| C | 1.197718000 | -0.453757000 | -1.254946000 |
| H | 1.827838000 | -1.321982000 | -1.074633000 |
| C | 2.021862000 | 0.814571000 | -1.508852000 |
| C | 3.166576000 | 0.975786000 | -0.505550000 |

| | | | |
|---|---|---|---|
| H | 3.841168000 | 1.765280000 | -0.850619000 |
| H | 2.799067000 | 1.250910000 | 0.485882000 |
| H | 3.747612000 | 0.051814000 | -0.412720000 |
| C | 2.625347000 | 0.540939000 | -2.905932000 |
| H | 3.203606000 | -0.388734000 | -2.918611000 |
| H | 1.846079000 | 0.473769000 | -3.669163000 |
| H | 3.299127000 | 1.361059000 | -3.169997000 |
| C | 1.202532000 | 2.105907000 | -1.569023000 |
| H | 0.304640000 | 2.007032000 | -2.185655000 |
| H | 0.896501000 | 2.430831000 | -0.573649000 |
| H | 1.822889000 | 2.895398000 | -2.004367000 |
| Br | -1.173637000 | -2.664658000 | 0.014927000 |

40

E (1Br'-tBu_1,2-insert-pdt) = -3294.05185123 a.u.

| | | | |
|---|---|---|---|
| C | -0.254630000 | -1.507110000 | -0.263282000 |
| B | 1.358410000 | 0.377183000 | 0.504562000 |
| C | -1.798141000 | 0.341178000 | 0.670082000 |
| C | -3.711060000 | -0.363101000 | 2.028403000 |
| H | -4.596806000 | -0.093905000 | 2.594487000 |
| C | -3.309773000 | -1.689126000 | 1.925066000 |
| H | -3.868500000 | -2.479130000 | 2.415812000 |
| C | -2.174017000 | -1.995061000 | 1.189001000 |
| H | -1.846836000 | -3.027917000 | 1.120997000 |
| C | -1.409292000 | -1.009333000 | 0.543609000 |
| C | 1.050936000 | -0.965676000 | -0.203943000 |
| C | -0.487101000 | -2.623359000 | -1.077727000 |
| H | -1.493311000 | -3.025137000 | -1.143048000 |
| C | 0.531762000 | -3.201920000 | -1.826175000 |
| H | 0.313678000 | -4.051826000 | -2.465358000 |
| C | 1.823674000 | -2.692065000 | -1.750511000 |
| H | 2.628825000 | -3.142166000 | -2.321473000 |
| C | 2.071312000 | -1.594747000 | -0.936160000 |

| | | | |
|---|---|---|---|
| H | 3.078580000 | -1.195319000 | -0.874475000 |
| H | -3.261298000 | 1.661286000 | 1.506447000 |
| C | -2.953112000 | 0.623486000 | 1.411154000 |
| C | 0.387094000 | 1.588204000 | 0.697553000 |
| H | 0.912970000 | 2.491068000 | 0.360047000 |
| H | 0.306999000 | 1.728641000 | 1.786826000 |
| C | -1.037175000 | 1.539231000 | 0.115627000 |
| H | -1.555655000 | 2.399890000 | 0.557432000 |
| C | -1.165236000 | 1.825904000 | -1.419872000 |
| C | -2.613554000 | 1.564921000 | -1.850935000 |
| H | -2.760410000 | 1.873739000 | -2.890916000 |
| H | -2.866867000 | 0.503844000 | -1.768735000 |
| H | -3.318967000 | 2.126384000 | -1.227743000 |
| C | -0.858977000 | 3.316753000 | -1.641942000 |
| H | -1.525529000 | 3.951613000 | -1.048691000 |
| H | 0.173429000 | 3.566098000 | -1.378327000 |
| H | -0.998250000 | 3.576225000 | -2.696093000 |
| C | -0.227431000 | 1.022686000 | -2.328438000 |
| H | 0.824373000 | 1.159576000 | -2.053798000 |
| H | -0.445860000 | -0.046728000 | -2.318947000 |
| H | -0.342105000 | 1.378136000 | -3.358279000 |
| Br | 3.161651000 | 0.676415000 | 1.171199000 |

# 8. References


1 G. P.M. van Klink, H. J. R. de Boer, G. Schat, O. S. Akkerman, F. Bickelhaupt and A. L. Spek, *Organometallics*, 2002, **21**, 2119-2135.

2 C. J. Berger, G. He, C. Merten, R. McDonald, M. J. Ferguson and E. Rivard, *Inorg. Chem.*, 2014, **53**, 1475–1486.

3 D. S. Wilbur, M-K. Chyan, D. K. Hamlin, B. B. Kegley, R. Risler, P. M. Pathare, J. Quinn, R. L. Vessella, C. Foulon, M. Zalutsky, T. J. Wedge and M. F. Hawthorne, *Bioconjugate Chem.*, 2004, **15**, 203-223.

4 H. Wang, J. Zhang, Z. Lin and Z. Xie, *Chem. Commun.*, 2015, **51**, 16817-16820.

5 G. Sheldrick, *Acta Cryst.*, 2015, **A71**, 3–8.

6 G. Sheldrick, *Acta Cryst.*, 2008, **A64**, 112–122.

7 (*a*) Y. Zhao and D. G. Truhlar, *Theor. Chem. Acc.*, 2008, **120**, 215-241; (*b*) Y. Zhao and D. G. Truhlar, *J. Chem. Theory Comput.*, 2008, **4**, 1849-1868; (c) N. Mardirossian and M. Head-Gordon, *J. Chem. Theory Comput.*, 2016, **12**, 4303-4325.

8 (*a*) W. J. Hehre, R. Ditchfield and J. A. Pople, *J. Chem. Phys.*, 1972, **56**, 2257-2261; (*b*) P. C. Hariharan and J. A. Pople, *Theor. Chim. Acta*, 1973, **28**, 213-222.
